# Learning a Prior on Regulatory Potential from eQTL Data

**DOI:** 10.1371/journal.pgen.1000358

**Published:** 2009-01-30

**Authors:** Su-In Lee, Aimée M. Dudley, David Drubin, Pamela A. Silver, Nevan J. Krogan, Dana Pe'er, Daphne Koller

**Affiliations:** 1 Computer Science Department, Stanford University, Stanford, California, United States of America; 2 Institute for Systems Biology, Seattle, Washington, United States of America; 3 Department of Systems Biology, Harvard Medical School, Boston, Massachusetts, United States of America; 4 Department of Cellular and Molecular Pharmacology, University of California San Francisco, San Francisco, California, United States of America; 5 Department of Biological Sciences, Columbia University, New York, New York, United States of America; University of Toronto, Canada

## Abstract

Genome-wide RNA expression data provide a detailed view of an organism's biological state; hence, a dataset measuring expression variation between genetically diverse individuals (eQTL data) may provide important insights into the genetics of complex traits. However, with data from a relatively small number of individuals, it is difficult to distinguish true causal polymorphisms from the large number of possibilities. The problem is particularly challenging in populations with significant linkage disequilibrium, where traits are often linked to large chromosomal regions containing many genes. Here, we present a novel method, Lirnet, that automatically learns a *regulatory potential* for each sequence polymorphism, estimating how likely it is to have a significant effect on gene expression. This regulatory potential is defined in terms of “regulatory features”—including the function of the gene and the conservation, type, and position of genetic polymorphisms—that are available for any organism. The extent to which the different features influence the regulatory potential is learned automatically, making Lirnet readily applicable to different datasets, organisms, and feature sets. We apply Lirnet both to the human HapMap eQTL dataset and to a yeast eQTL dataset and provide statistical and biological results demonstrating that Lirnet produces significantly better regulatory programs than other recent approaches. We demonstrate in the yeast data that Lirnet can correctly suggest a specific causal sequence variation within a large, linked chromosomal region. In one example, Lirnet uncovered a novel, experimentally validated connection between Puf3—a sequence-specific RNA binding protein—and P-bodies—cytoplasmic structures that regulate translation and RNA stability—as well as the particular causative polymorphism, a SNP in *Mkt1*, that induces the variation in the pathway.

## Author Summary

Gene expression data of genetically diverse individuals (eQTL data) provide a unique perspective on the effect of genetic variation on cellular pathways. However, the burden of multiple hypotheses, combined with the challenges of linkage disequilibrium, makes it difficult to correctly identify causal polymorphisms. Researchers traditionally apply heuristics for selecting among plausible hypotheses, favoring polymorphisms that are more conserved, that lead to significant amino acid change, or that reside in genes whose function is related to that of the targets. But how do we know how much weight to attribute to different regulatory features? We describe Lirnet, which learns from eQTL data how to weight regulatory features and induce a *regulatory potential* for sequence variations. Lirnet assesses these weights simultaneously to learning a regulatory network, finding weights that lead to a more predictive network. We show that Lirnet constructs high-accuracy regulatory programs and demonstrate its ability to correctly identify causative polymorphisms. Lirnet can flexibly use any regulatory features, including sequence features that are available for any sequenced organism, and automatically learn their weights in a dataset-specific way. This feature makes it especially advantageous for mammalian systems, where many forms of prior knowledge used in simple model organisms are incomplete or unavailable.

## Introduction

The potential for using comprehensive data sets, such as RNA expression data, as a means for uncovering complex genetic traits has led to the production of eQTL data – gene expression data across a population of genetically diverse individuals – in a variety of different organisms [1]–[6]. One application of this data type is the use of subtle perturbations in the regulatory network induced by natural genetic variations to reveal the regulatory interactions and influences. These data thus provide a unique opportunity for uncovering the cell's regulatory structure, and for revealing the genetic basis for phenotypic traits. Many approaches have been developed that attempt to identify one or more genetic regions containing polymorphism(s) that cause a change in gene expression [1]–[3],[7],[8]. Some approaches [7],[8] expand these ideas by searching for a more integrated regulatory network, where targets are viewed as affected not only by changes in genotype, but also by changes in the activity level of regulatory proteins, estimated by their mRNA levels. These methods have been used successfully to identify important regulatory relationships, including some that underlie key phenotypic traits.

A key challenge in the application of these methods is that the number of candidate *regulators* is enormous relative to the amount of available data, making it difficult to robustly identify the correct regulator. This problem is exacerbated when multiple regulators are correlated, and therefore many regulators have similar potential to explain the expression of their targets. Unfortunately, correlated regulators are the rule, rather than the exception, both for sequence polymorphisms (due to linkage disequilibrium) and for regulatory genes identified by gene expression signature (due to co-expression). In these cases, methods that attempt to recognize regulatory relationships are often forced to make choices that are arbitrary, misleading, or non-specific. For example, most linkage-based approaches identify only a chromosomal region, leaving a human to predict the true causal polymorphism(s) within the region. This approach results in a large number of hypotheses for experimental testing, especially in higher organisms, such as humans where chromosomal regions are often very large and methods of experimental validation are time and labor intensive.

**Figure 1:**
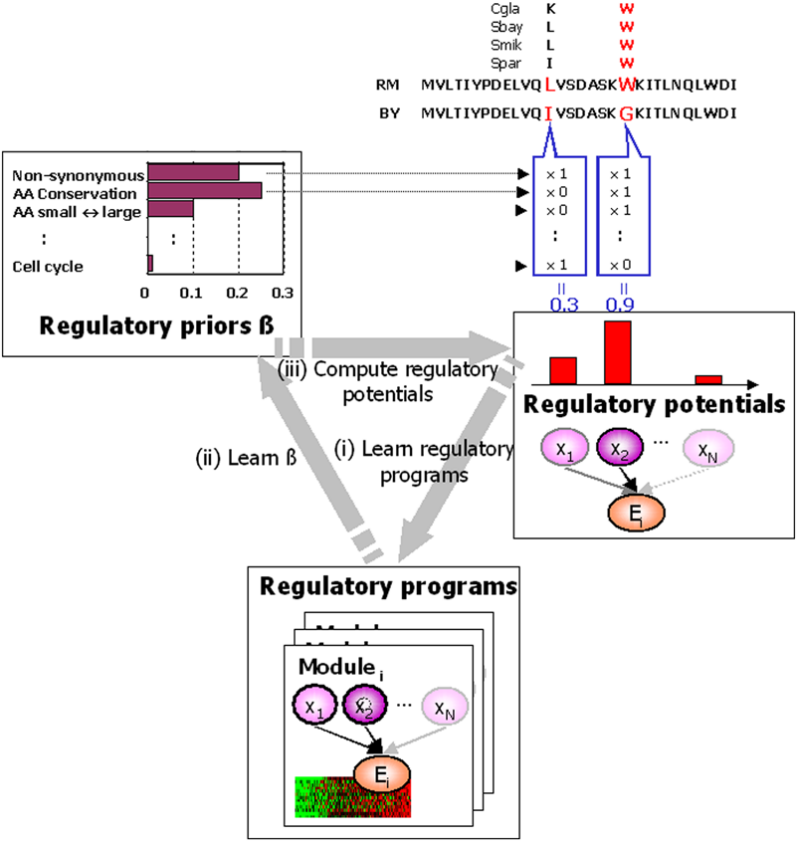
Outline of our approach. Our algorithm, called Lirnet, aims to learn the regulatory potential of an individual SNP, simultaneously with the regulatory network from an eQTL data set. The regulatory potential of a regulator is defined as a function of its *regulatory features*, such as the conservation of a SNP or the function of a gene (Figure 2, Tables S1, S2, S3). The weight of each regulatory feature is called the *regulatory prior*. All three components – the regulatory programs, the regulatory potentials, and the regulatory priors – are learned from data, in an unbiased way, by iterating the following three steps: (i) Lirnet takes as input the regulatory potentials for each regulator, and constructs a set of regulatory programs for the genes in the data, using the regulatory potentials to bias the choice of active regulators used. In the first iteration, the regulatory potentials are taken to be uniform. (ii) Lirnet takes as input the regulatory programs, and learns which types of regulators are more predictive of their putative targets (which ones occur more often in the learned regulatory programs), and adjusts the regulatory prior to match the observed trends. (iii) Lirnet takes as input the regulatory priors, and computes the regulatory potential of each SNP by computing the total contribution of its regulatory features, weighted by the learned regulatory priors. The regulatory potential of each chromosomal region (genotype regulator) is then computed by aggregating the contributions of the individual SNPs in the region.

**Figure 2:**
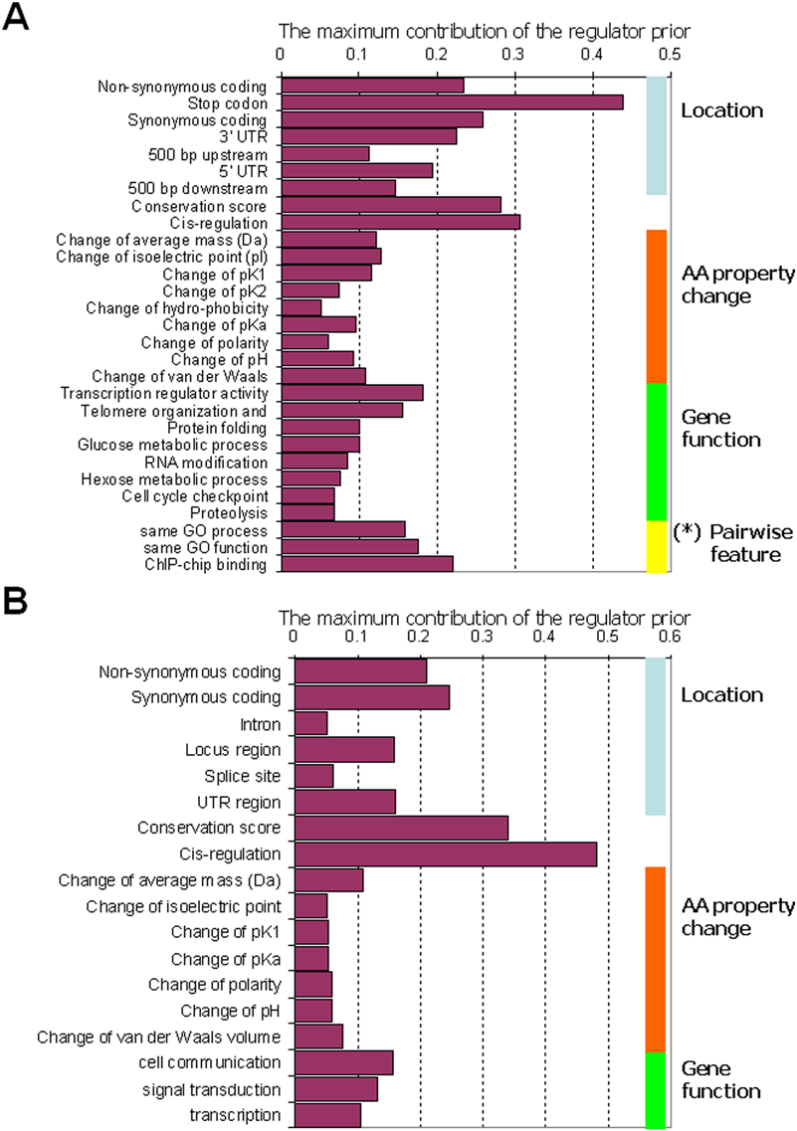
Learned regulatory priors in yeast and human. Shown are the maximum effect of the different regulatory features, given the learned regulatory priors for the different regulatory features, in (A) yeast and (B) human (CEU) data sets. Each bar lists the maximum contribution that a given regulatory feature can make to the regulatory potential: the feature's regulatory prior, multiplied by the difference between its maximal and minimal value. For clarity, only the regulatory features whose regulatory priors are greater than 0.05 are shown in this graph. The full list of regulatory priors, including that for human YRI dataset, can be found in Tables S2, S3. (*) As described in Methods, the pairwise features are constructed based on -log(p-value), indicating the enrichment of the corresponding regulator's putative targets in the module. Since these values have much higher variation than others, for a more clear and intuitive presentation, we report the amount of contribution made by an increase of the −log_10_(p-value) by 3.

In this study, we propose a novel approach based on the observation that not all candidate regulators are equally likely to play a causative role in gene expression. Indeed, researchers often manually select among candidate polymorphisms, favoring those that are in conserved regions, those that produce significant changes in protein sequence, or those that lie in functionally relevant genes (such as transcription factors or signaling proteins). However, it is not clear how to weight these different features, and, indeed, their relevance might vary across organisms, tissues, or even conditions. We propose a novel Bayesian method, called Lirnet, that automatically learns three model components (Figure 1): a regulatory network; a set of *regulatory potentials* for all candidate regulators, evaluating the likelihood that they play a causal role; and a set of *regulatory priors*, which define a regulator's regulatory potential in terms of its *regulatory features*, such as the conservation of a SNP or the annotated function of a gene (see Methods; Figure 2, Tables S1, S2, S3). All three components are learned from the data, in an unbiased way, using an iterative approach: As Lirnet constructs a set of regulatory programs for the genes in the data, it learns which types of regulators are more predictive of their putative targets; it adjusts the regulatory prior to match the observed trends, and it then relearns the regulatory programs using the adjusted prior (Figure 1). Thus, the method automatically tailors itself to the regulatory interactions in a particular data set. Moreover, Lirnet can use any set of features that are likely to indicate regulatory potential, including sequence features (such as conservation or significance of amino acid change) that are available for many organisms. This feature, combined with Lirnet's ability to learn the importance of these features automatically, makes it especially advantageous for mammalian systems, where many forms of prior knowledge used in simple model organisms are incomplete or unavailable.

Recently, there have been several approaches for identifying a causal gene in eQTL data [9],[10]. Zhu et al [9] learned a Bayesian network from the eQTL data. They show that incorporating various other genomic data such as transcription factor binding sites (TFBS) and protein-protein interaction (PPI) data improves the quality of the learned network. They also use the network for identifying the most likely causal regulator in a genomic region. For a group of genes linked to a given region and a candidate regulator in the region, they test the overlap between the linked genes and the genes regulated by the candidate in the learned network. Suthram et al. [10], building on earlier work of Tu et al. [11], propose an alternative method called eQED, which also aims to select a particular regulation within a linked region. These methods define an electric-circuit model for the flow of influence in a separate PPI network and use it to select he most relevant regulator in the region.

These methods, like ours, utilize domain knowledge encoded in TFBS or PPI data for identifying causal regulators. However, these methods do not incorporate any information on properties of individual SNPs, such as their conservation score (a feature that was indeed chosen to be important in our automated analysis). Moreover, both methods are biased towards discovering regulatory relationships involving transcription factors. In comparison, Lirnet uses a broad range of regulatory features, enabling the identification of novel regulatory relations, as well as those involving other mechanisms such as chromatin and mRNA degradation, as is demonstrated by Lirnet's experimentally validated hypothesis of a relationship between two post-transcriptional regulatory pathways.

An alternative approach is proposed by Jiang et al. [12], whose method prioritizes non-synonymous SNPs as being disease-related, based on various features such as weight and biochemical properties. Their predictor was trained on a database of over 20,000 non-synonymous SNPs, annotated with a disease level for each SNP, and achieved a high prediction performance. Although this method takes the SNP-specific features as input and prioritizes individual SNPs, it does not incorporate the gene-based and network-based features that are used in our analysis, as well as the ones of Zhu and Suthram. It is also restricted to the analysis of non-synonymous coding SNPs, and focuses on the relevance to a single phenotype (i.e. a disease).

We test our approach using two eQTL data sets, selected to assess method's versatility. The first is the HapMap data set of Stranger et al. [4], which contains expression profiles for lymphoblastoid cell lines generated from participants in the human HapMap study. The second is the yeast data set of Brem and Kruglyak [3], which measures the mRNA expression and genotype of 112 recombinant progeny generated by mating of two genetically diverse strains of *S. cerevisiae*, a laboratory strain (BY) and a wild vineyard strain (RM). We show statistically that the learned regulatory potential significantly improves the quality of the learned regulatory programs, as evaluated by the percent of the variance explained. We also evaluate the biological validity of our learned regulatory programs by comparing them to other biological data, not used within the algorithm. Our results clearly demonstrate that Lirnet produces more accurate regulatory programs than previous approaches, including Geronemo [7] and the recent methods of Suthram et al [10] and Zhu et al. [9].

We also provide a detailed analysis of some of the inferred yeast regulatory programs, and demonstrate that Lirnet can correctly identify the causative polymorphism within a large, linked region, even in regions containing several biologically plausible candidates. We study in greater depth one of the pathways produced by Lirnet, involving two modules related to post-transcriptional gene regulation. In this case, Lirnet suggested a three-tiered regulatory cascade: at the lowest level, a module comprising a set of genes that are bound by the sequence-specific RNA binding protein, Puf3; the module's predicted regulatory program, which utilizes factors involved in several distinct post-transcriptional regulatory processes, including members of the P-body complex, an RNA storage and degradation complex that can also modulate mRNA translation; and at the highest level, a chromosomal region containing the causal variation, and, using its learned regulatory prior, even a particular gene in the region – Mkt1, whose protein product binds (indirectly) to the PolyA-binding protein at the 3′ region of mRNA transcripts [13]. We provide multiple forms of experimental data supporting Lirnet's computational prediction, including the causal role of Mkt1.

The resulting regulatory network for the yeast data and the software are freely available on our website http://dags.stanford.edu/lirnet/; the learned network can be effectively explored using our visualization tool, downloadable from the same website.

## Results

### Method Overview

We briefly review the Lirnet method, referring to the Methods for a full description. Lirnet uses genotype and expression data of genetically diverse individuals (eQTL data) and aims to learn a regulatory prior concurrently with reconstructing a regulatory network. Building on earlier work [7],[14], Lirnet clusters genes into *modules* with the assumption that expression of the target genes in each module is governed by the same regulatory program. As with several other methods for the reconstruction of regulatory networks, Lirnet can accommodate two types of regulators: values of genotype markers (genotype regulators), representing genetic polymorphisms on chromosomal regions [1]–[3]; and expression levels of genes that are known to have regulatory roles (expression regulators), representing activity levels of genes that might regulate that module [7],[9],[14],[15].

Lirnet's regulatory programs are based on linear regression, a choice designed to allow for the incorporation and learning of regulatory potentials. For each module *m*, the expression levels of genes in the module (denoted by *y_m_,_j_*) are modeled as a linear regression of candidate regulators (denoted by *x_1_*,…,*x_n_*): *y_m_,_j_∼w_m,1_x_1_+w_m,2_x_2_+…+w_m,n_x_n_*, where all genes in the module share the same weights *w_m,k_* A regulator *r* that has a zero weight *w_m_,_r_* has no effect on the expression of the targets in module *m*. A biologically plausible regulatory program should have a small number of regulators with a non-zero weight. To achieve this goal, we use the LASSO method [16] to select only the most significant regulators. In its simplest form, LASSO adds a fixed penalty term to the objective function that introduces a uniform bias towards sparsity in the weights *w*.

Lirnet, inspired by our recent work on feature selection [17], incorporates regulatory potential by allowing different regulators to have different sparsity biases; a regulator *r* whose regulatory potential is low for a module *m* will have a stronger bias towards *w_m,r_* = 0, and thus a lower probability of being selected as an active regulator for that module. The regulatory potential *C_r_* of regulator *r* is defined to be a function of its *regulatory features f_r_*. The method flexibly accommodates any property or combinations of properties of a regulator (Figure 2, Tables S1, S2, S3) that might be indicative of its likelihood of having a causal effect on its targets. These features can include features of the regulator alone, such as the location and significance of sequence polymorphisms, the function of the gene (transcription factor, signaling protein, etc.), and conservation of the polymorphic site. They can also include features that involve both the regulator and the targets, such as the enrichment of the module with genes having known relationships to regulator (e.g. transcription factor targets). The *regulatory prior β* encodes the importance given to each regulatory feature (see Methods), and is automatically learned from the data, allowing less relevant regulatory features to be ignored and others to manifest their significance.

The learning algorithm of Lirnet jointly estimates *w_m,r_*'s and *β* by maximizing a joint objective that involves both. The algorithm iterates three steps until convergence (Figure 1): (1) learning the regulatory program for each module by estimating the weights *w_m,r_*'s; (2) learning the regulatory priors *β* that reflect the importance of each regulatory feature; (3) computing the regulatory potential for each candidate regulator, module pair, based on the current *β*, thereby biasing their selection in the next iteration's regulatory programs. The output of Lirnet is thus threefold. First, it constructs a set of learned regulatory programs for the modules used in the analysis; for module *m*, these are all the regulators *r* with a non-zero weight *w_m,r_*. Second, it constructs a quantitative regulatory potential both for genes and for specific sequence polymorphisms within them, allowing us to rank candidates for the causal sequence variation and to prioritize hypotheses for further testing. Third, it produces a set of regulatory priors, which may provide insight on the properties of a polymorphism that tends to induce an effect on its downstream targets.

### Statistical Evaluation on Yeast and Human Data

To test the versatility and generality of Lirnet, we applied it to two very different eQTL data sets. The first is the eQTL data set of Brem and Kruglyak [3], which measured the mRNA expression and genotype of 112 *S. cerevisiae* strains derived as the F2 progeny of a BY/RM cross. The second is the expression data measured in the lymphoblastoid cell lines of the 60 unrelated HapMap individuals in the CEU data set, using only the genotypes of a subset of markers (the 500 K tag SNPs on the Affymetrix chips) as regulators (see Methods).

Figure 2 shows the regulatory priors of the most significant regulatory features in each of these data sets, as identified by the Lirnet algorithm. Several aspects of the features automatically chosen as important by the algorithm are revealing. First the top learned regulatory potentials between two organisms as different as human and yeast are remarkably consistent, supporting the robustness of our approach. For example, aside from the feature indicating the stop codon, which affects only 43 genes, the strongest positive weight on the regulatory potential in both datasets is given to a feature denoting whether the sequence variation correlates with changes in the expression level of the closest gene (cis-eQTL). This gene-level feature indicates that the polymorphism is already causal towards a change in the cell (the expression level of the gene) and hence may have additional downstream effects. The second most significant regulatory feature, also in both data sets, is the conservation score, consistent with the hypothesis that changes in residues conserved across millions of evolutionary years are more likely to have a causative influence. Also with a significant weight are features that evaluate the functional relevance of the position of the polymorphism, and the significance of the actual change. These features include, for example, the presence and type of amino acid changes, and polymorphisms in the 5′ or 3′ UTR. Surprisingly, in both data sets, synonymous SNPs weigh more heavily than non-synonymous ones. This phenomenon might arise from the fact that synonymous SNPs can have an effect on translational efficiency or mRNA destabilization [18], consistent with recent findings that such SNPs are under significant purifying selection [19],[20]. The selection of this regulatory feature by our automated method lends support to this hypothesis, and is worthy of further investigation.

Aside from sequence-based features, we also provided the method a rough categorization of gene function, allowing it to learn which types of genes are likely to play a regulatory role. Despite receiving no prior knowledge about the relative importance of the various functional categories, the method automatically assigns high weight to functional categories with a regulatory role: In the human data, the highest weights among those features are given to genes involved in cell death, transport, cell growth, signal transduction, transcription, and cell communication. In the yeast data, ‘transcription regulator activity’, ‘telomere organization and biogenetics’, ‘protein folding’, ‘glucose metabolic processes’, and ‘RNA modification’ are chosen to be important. Notably, other work supports the differences between BY and RM in many of these processes, including glucose processing (BY and RM demonstrate dramatically different growth rates on a number of carbon sources including glucose; D. Pe'er, unpublished data) and telomere organization [7], showing the value of allowing the regulatory priors to be tailored to particular data sets and organisms.

Lirnet can also take advantage of other functional data, when available. For example, in the yeast data, the pairwise feature derived from ChIP-chip binding between the regulatory gene and targets in the module received a relatively high weight. We note, however, that the method is also effective when such data are not available for a given transcription factor and set of target genes (see Oaf1 example below) or when the features themselves are not available, as in the case of the human data.

**Figure 3:**
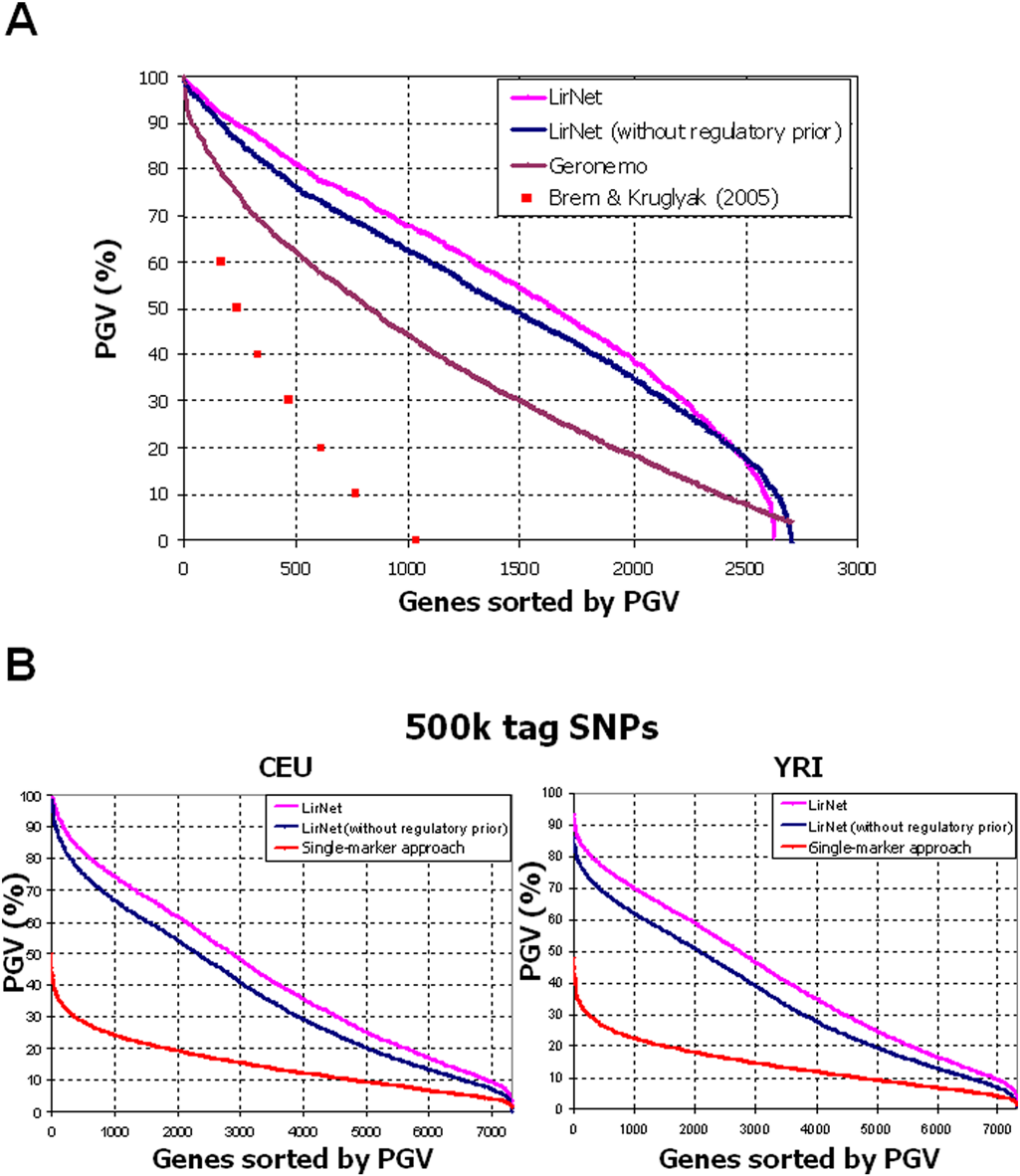
Statistical evaluation of learned regulatory programs. Proportion of genetic variation in gene expression explained by different methods. The percentage of genetic variation (PGV) explained by detected regulation programs for Lirnet with learned regulatory potential (pink), Lirnet with uniform regulatory potential (blue). (A) The PGV curves for the yeast data with additional comparison to Geronemo (brown) and the eQTL analysis of Brem & Kruglyak (red points) applied to the same dataset. The graph shows the PGV_g_ values (y-axis) of 3152 genes (x-axis). The genes (x-axis) are sorted by their PGV_g_, shown on the y-axis. A more refined PGV analysis, with an independent test set is shown in Figure S1A. (B) The PGV results for the human HapMap data with 500 k tag SNPs (Affymetrix), for both the CEU and YRI individuals. Similarly, we compare Lirnet (pink) to the variant with a uniform regulatory potential (blue) and to a classical single-marker approach (red; see Methods). Results for 100 k tag SNPs are shown in Figure S1D, and the results with an independent test set is shown in Figure S1B & C for 500 k and 100 k tag SNPs, respectively.

We next tested whether the learned regulatory potentials improved the quality of the learned regulatory program, by computing the proportion of genetic variance (PGV) explained by the learned program. We compared Lirnet with a uniform regulatory potential (hereafter “flat” Lirnet), Lirnet with a learned regulatory potential, a standard single-marker linkage (as in [1]–[3]) and Geronemo [7]. The results (Figure 3) demonstrate that Lirnet explains a dramatically larger fraction of the variance for a much larger set of genes than all the other methods. For example, in the yeast data, Lirnet with learned regulatory potentials explains over 50% of the PGV for 1,644 genes, compared to 1,457 genes for flat Lirnet, 828 genes for Geronemo and 230 genes for the method of Brem & Kruglyak. The same advantage translates across the spectrum of PGV values, and is arguably even greater at the tail, where many genes that are very poorly explained by other methods have a considerable fraction of PGV explained by Lirnet. A more refined PGV analysis, with an independent test set, shows that this dramatic improvement does not arise from overfitting to the test data (Figure S1). In fact, the Lirnet model has a comparable number of parameters to Geronemo and fewer parameters than the method of Brem and Kruglyak, due to the use of modules.

Notably, even flat Lirnet considerably outperformed both the single-marker linkage and Geronemo methods, suggesting that Lirnet would still perform well in cases where the learned regulatory potential was less informed. The gap between Lirnet and flat Lirnet appears to increase when using more markers (see Figure S1D for human results on 100 K markers), and is larger in the YRI than in the CEU data. These results are consistent with a hypothesis that the benefits of a non-uniform regulatory prior are more pronounced when regulatory potentials are aggregated over smaller regions of linkage disequilibrium. Thus, one can expect the benefits of Lirnet's learned regulatory priors to grow as we move to denser genotyping.

### Recovery of Known Regulatory Pathways

We evaluated how well the learned regulatory program recovers known regulatory interactions. We begin with a comprehensive analysis of the quality of the learned regulatory programs, demonstrating that they are consistent with other sources of data indicating regulatory interactions that were not provided to the Lirnet algorithm. We then provide a comparison to the state-of-the-art method of [9], which was applied to the same data.

As a gold-standard set of regulator-target relationships is not available, we constructed a comparison test set from various datasets: deletion and over-expression microarrays [21],[22]; chromatin immune-precipitation (ChIP-chip) binding experiments [23]; mRNA binding pull-down experiments [31]; transcription factor binding sites [65]; and a literature-curated set of signaling interactions from the Proteome database (http://www.proteome.com/). Although each of these data sets has its own limitations in terms both of false negatives and of false positives, agreement with these orthogonal data sources is a reasonable metric for evaluating the quality of a method's predictions. For a prediction that a regulator *r* regulates a module *m*, we defined it to be validated if there was significant overlap (hypergeometric p<0.01) between the members of *m* and the putative targets of *r*, suggested by one of the above datasets. We note that none of these datasets was used for constructing the regulatory features for Lirnet: Lirnet used only the ChIP-chip data set of [24], and all regulator-target pairs that appeared in these data were removed from the evaluation data [23].

Most of these data sets focus on regulatory relationships where *r* is a transcription factor, whereas Lirnet and the other methods we evaluate are also capable of identifying cases where *r* plays a different regulatory role, such as signaling, chromatin modification, or RNA degradation. Therefore, to increase the coverage of our validation effort, we also considered indirect regulatory relationships (Two-Step Cascade in Table 1), where a method predicted a regulator *r* that has some close relationship with a transcription factor *t*, and *t* is confirmed in the above data sets to regulate *m*. We considered cases where *t* and *r* have a reliable protein-protein interaction (PPI) (Xenarios et al. 2000); and cases where *r* phosphorylates *t* in the Proteome data set.

**Table 1:** Biological evaluation of the learned regulatory program.

	Direct		Two-Step Cascade	
	# interactions	# modules	# interactions	# modules
Lirnet	32/123 (26.02%)	25/49 (51.02%)	101/173 (58.38%)	45/53 (84.91%)
Lirnet without regulatory prior	28/122 (22.95%)	21/50 (42.00%)	88/162 (54.32%)	44/52 (84.62%)
Geronemo	16/105 (15.24%)	13/57 (22.81%)	81/158 (51.27%)	43/62 (69.35%)
Random Model	9/110 (8.18%)	9/58 (15.52%)	39/151 (25.83%)	29/62 (46.77%)

We evaluated our learned regulatory programs relative to a reference set of regulatory interactions collected from various datasets that were not used by the Lirnet method (see text for more details). A prediction that a regulator *r* regulates a module *m* was considered as validated if there was significant overlap (hypergeometric p<0.01) between the members *of m* and the putative targets of *r* in the reference set above. For each method, we counted the number of validated interactions (column named # interactions) for module *m* containing ≥10 genes, where each entry shows: *a/b* (*c*%), where *a* is the number of significant regulators, *b* is the total number of predicted regulators that appear at least once in the reference dataset, and *c* is the proportion (*a/b*×100). We similarly counted the number of modules that have at least one validated regulator (column named #modules), relative to the total number of modules having a predicted regulator in the reference set. We also considered two-step regulatory cascades, as described in the main text. Table S4 shows this analysis for expression regulator and genetic marker regulators separately.

Table 1 summarizes the number of validated regulators for various models, applied to the same set of modules: Lirnet with a uniform regulator potential, Lirnet with the learned regulatory potential, Geronemo, and a random model. (See also Table S5 for a full list of Lirnet predictions and their support.) Overall, Lirnet recovers a larger fraction of the known regulatory interactions than the other methods. We note that the reference set supports only a subset of the predicted regulatory interactions. This fact is not surprising, as the data sources used for constructing the reference set focus on transcriptional regulation, whereas Lirnet and Geronemo cover a much larger set of regulatory relationships. Although we have made some attempt to expand our reference set to cover signaling interactions, the data set of literature-curated signaling interactions is only a small fraction of the total set of signaling interactions that presumably hold in yeast. Moreover, as shown in our previous study [7], a large part of the regulatory interactions in these data sets represent chromatin modification and post-transcriptional regulation, which are not represented in our reference set.

The BY/RM cross also exhibits a large amount of cis-regulation, which we did not explicitly model in the Lirnet analysis. Nevertheless, of 492 cis-regulated genes – those whose nearby marker is significantly predictive of its expression level (t-test p-value<1e-5; 12.8% of the 3152 genes used in our analysis), 307 genes (76.4%) are assigned to modules with cis-regulatory programs. More specifically, 149 genes are assigned to modules that have the genes' nearby markers as genetic marker regulators; 158 other genes are assigned to modules that have their nearby expression regulators having their markers in the regulators' regulatory programs, suggesting an indirect cis-effect from a locus to an expression level of a regulator in that region to a target.

**Figure 4:**
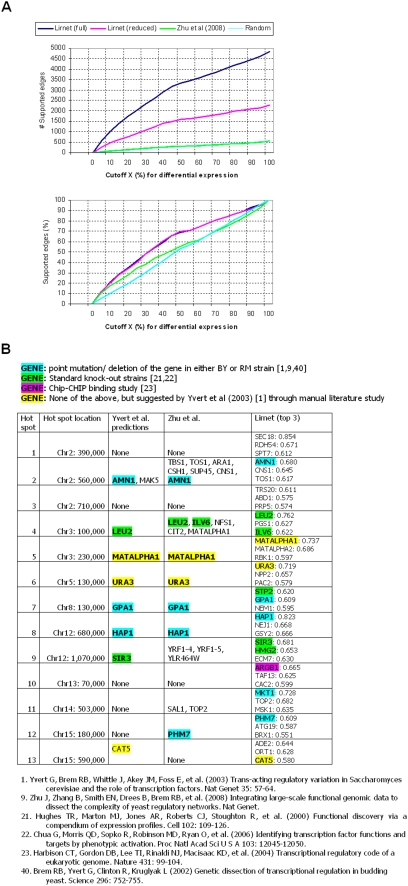
Evaluation of the learned network in comparison to results of Zhu et al. [9]. We compared two versions of Lirnet results with the learned network of Zhu et al: all 10,565 regulator-target pairs from the regulatory network (‘full’ in the graph legend); 3,645 top-ranked pairs, in terms of the magnitude of the weight, to provide a comparable number of predictions to the network of Zhu et al (‘reduced’ in the graph legend). We evaluated support for these sets of edges in the gene expression data of [21],[22]. Here, a pair *r-t* for a regulator *r* and target *t* is considered supported if *t* is in the top X% of differentially expressed genes in response to a knockout or over-expression of R. (A) Shows the cumulative distribution of the number of computational predictions that receive support for different values of X (top). As a baseline, we also show the number of validated predictions expected in a random regulatory network. Not all regulators were tested in the microarray data. To avoid possible biases, we also compare the fraction of validated predictions among all predictions that were tested (bottom). We see that Lirnet selects many more tested predictions than the method of Zhu et al., but also has a much higher fraction of validated predictions, even when we focus only on tested predictions. (B) Candidate causal regulators for 13 chromosomal regions identified in a previous study. For the 13 hot spots previously suggested [1], we applied our approach to compute the regulatory potential to prioritize the candidate genes in each region. The first four columns are from the paper by Zhu et al [9]. For each hot spot, we present the causal regulators suggested by: the original paper of [1]; the method of Zhu et al, and the top 3 Lirnet regulators, ranked by their regulatory potentials (see Methods). The causal regulators that have some support (see Methods) are colored accordingly (see legend). Of the top Lirnet regulators, 14 regulators, spanning 11 hot spots, have experimental support, in comparison to 8 regulators (7 hot spots) in the analysis of Zhu et al. Even if we consider only Lirnet's top regulator for each region, there is experimental support for 10 regulators (in 10 hot spots). The results of the previous method (first four columns) are from Table 3 of Zhu et al [9], except for the indication of the supported regulators.

We also compared our results with those of the recent Bayesian network method of [9]. As this method infers regulatory interactions for individual genes rather than modules, we used the number of regulator-target pairs validated in the reference set as our evaluation metric. For a fair comparison, we removed from the reference set any data sets that are used for learning either model, leaving only the deletion and over-expression microarray data [21],[22]. The results (Figure 4A) show that, for various levels of expression change in the deletion or over-expression data sets, the regulatory interactions inferred by Lirnet are more consistent with previously known regulatory relationships. In Figure 4A, we see that Lirnet recovers many more supported regulators than the Zhu et al. method. This large discrepancy is partially due to the fact that their method largely focuses on transcription factors, and hence is incapable of picking up many of the regulatory relationships that are uncovered by Lirnet. However, even if we focus attention only on the relationships that could be compared to the deletion/over-expression data (Figure 4B), and evaluate the fraction that were validated in these data, we see that Lirnet significantly outperforms the Zhu et al. method.

### Predicting the Causative Sequence Polymorphism in a Large Chromosomal Region

One of Lirnet's key features is its ability to identify a specific causative regulator in a linked chromosomal region, an ability also presented in several other recent methods [9],[10]. Since other methods for comparison focused on identifying a causal gene not a specific SNP, we also prioritized genes for a direct comparison. We first compute the regulatory potential of all SNPs in the region of interest, relative to each module (to account for pairwise regulatory features). We then rank each gene based on the highest-scoring SNP associated with it. Figure S2 shows the overall distribution of the regulatory potentials for both SNPs and genes. We can see that the vast majority of SNPs and of genes have a fairly low regulatory potential. The distribution begins to tail off at a regulatory potential of about 0.694; only 0.22% of SNPs and 2% of genes have a regulatory potential that exceeds this value.

We first compare to the recent work of Zhu et al., who focus on 13 “hot spots” – chromosomal regions identified to regulate expression levels of a number of genes in a previous study [1]. Previous work has identified regulators for several of these hot spots, and Zhu et al. provide new experimental validation for a number of others. To compare to these results, we considered the genes linked to each hot spot as a “module”, and applied the learned regulatory prior (Figure 2) to individual SNPs in each region to compute each gene's regulatory potential (see Methods). We sorted the genes in each hot spot based on their regulatory potential, and listed the top 3 genes for each hot spot. Figure 4B compares the result of the suggested causative genes in each region between Lirnet and the method of Zhu et al. (2008). We see that, of the top Lirnet regulators, 14 regulators, spanning 11 hot spots, have experimental support (see Methods), in comparison to 8 regulators (7 hot spots) in the analysis of Zhu et al. Even if we consider only Lirnet's top regulator for each region, there is experimental support for 10 regulators.

We also compare to the recent method of Suthram et al. [10], which improves on earlier work of Tu et al. [11]. These methods consider a gene and a chromosomal region to which it is linked, and analyze the flow in a protein-protein interaction network to select a particular causal regulator within the region. Suthram et al. validate their results relative to a pre-defined set of 548 regulatory relationships, extracted from gene knockout or overexpression microarray studies [21],[25], similarly to our analysis above. The predicted network of Suthram et al. was not available, so we evaluated Lirnet using their protocol and the reference set, to allow for a direct comparison. For each target gene and linked region, we selected, as the Lirnet predicted regulator, the gene whose regulatory potential in the region was highest. We then evaluated these predictions using the reference set of Suthram et al. The results, shown in Table S6, show that Lirnet significantly outperforms both the method of Suthram et al. and the previous method of Tu et al. [11], according to this evaluation metric.

### Biological Results

We also performed an in-depth analysis of some of the specific regulatory modules produced by Lirnet for the yeast data set, and evaluated its ability to identify both the correct regulators and the specific polymorphisms that gave rise to the expression change in the targets.

### Transcriptional Regulators

**Figure 5:**
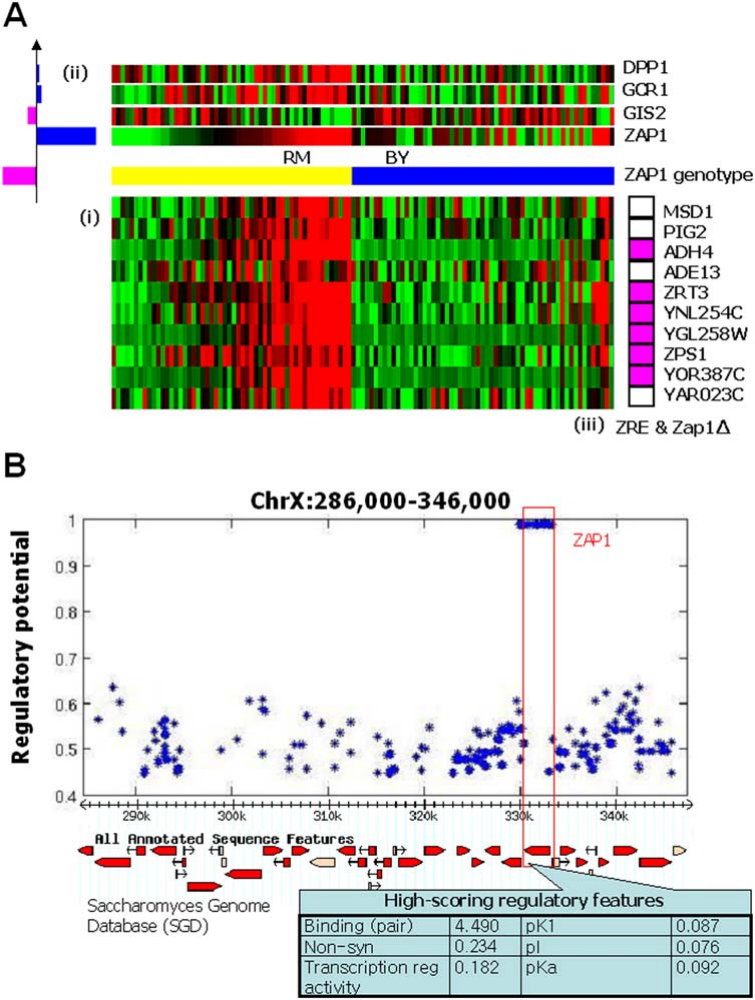
The Zap1 module. (A) (i) The mRNA expression profiles (log_2_ ratios) of the module's 10 target genes, where the rows are genes and the columns are strains. (ii) The module is regulated by five predicted regulators, where the two that have the most significant coefficients are the expression pattern of *ZAP1* and a genetic region on chromosome 10 containing *ZAP1*. The bar on the left of each regulator represents its coefficient in the regulatory program: the length encodes its absolute value, purple represents a negative weight and blue a positive one. (iii) Six of the target genes (ADH4, ZTR3, YNL254C, YGL258W, ZPS1/YOL154W, and YOR387C) were identified as probable Zap1 targets based on the presence of a consensus ZRE element and RNA expression patterns [27]. (B) The genetic region on chromosome 10, with the inferred regulatory potentials for each of the SNPs it contains (Table S7). Also shown are the regulatory features that contributed the most to the selection of a SNP in Zap1 as the causal polymorphism: a known binding relationship between Zap1 and two of the target genes, the presence of non-synonymous coding changes and their effect on various protein properties, and the gene's annotation as having transcriptional regulator activity. All the other minor regulators of this module (Dhh1, Gcr1 and Gis2) are not located in this region; they are in chr 4, 16 and 14, respectively.

One example of the predictive power of assigning regulatory potential to individual SNPs within a large chromosomal region is the Zap1 module (Figure 5A). The module contains ten target genes and two major regulators, the gene expression pattern of *ZAP1*, which encodes a transcription factor (TF) that activates genes in response to Zinc [26],[27], and a genetic region on chromosome 10 that contains *ZAP1*. Of the ten target genes in the module, six were among 40 probable Zap1 targets based on the presence of a consensus ZRE element and RNA expression patterns in zinc and in the absence of Zap1 (p = 5.7×10^−10^) [27]. While the causative role of Zap1 in this data has previously been affirmed a number of times [3], [7]–[9], Lirnet automatically identified polymorphisms within Zap1 as the ones most likely, within the linked region, to play a causal role (Table S7). The regulatory potential of the identified SNP is the highest over all yeast SNPs (Figure S2). The most significant regulatory feature by far in this identification was the known binding relationship between Zap1 and two of its target genes, but other features also played a role (Figure 5B). Thus, the method has identified a TF-target relationship for which there is significant biological support.

**Figure 6:**
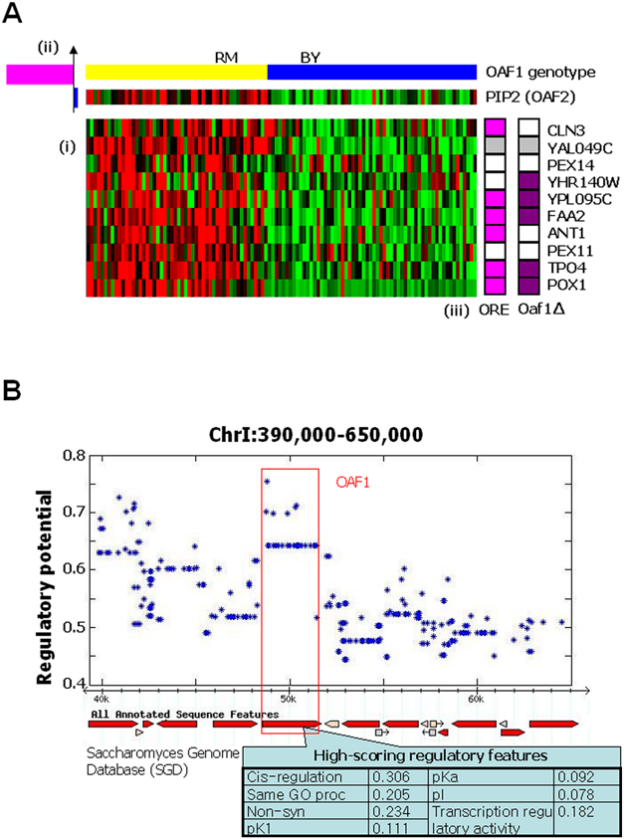
The peroxisome module. (A) The module contains 10 target genes (i), regulated by 2 predicted regulators (ii) – a genetic region on chromosome 1 containing OAF1, and the expression pattern of *PIP2*, the other component in the Oaf1-Pip2 heterodimer. (iii) Six of the target genes (*POX1*, *FAA2*, *TPO4*, *ANT1*, *YPLO95C* and *CLN3*) contain a canonical Oaf1 binding site (ORE) [29]. The two predicted regulators and five of the target genes are among the most significantly down regulated RNA transcripts in an *oaf1Δ* microarray with the following ranks: *POX1* (1^st^), *YPL095* (2^nd^), *FAA2* (5^th^), *YHR140W* (14^th^), *TPO4* (23^rd^), *OAF1* (9^th^), *PIP2* (29^th^). (B) The genetic region on chromosome 1, with the inferred regulatory potentials for each of the SNPs it contains (Table S8). Also shown are the regulatory features that contributed to the selection of a SNP in Oaf1 as the causal polymorphism.

Importantly, however, Lirnet is also able to predict such relationships when relevant functional data such as binding assays are not available. One such example is the peroxisome module (Figure 6A), containing ten genes that are enriched for processes related to fatty acid metabolism (hypergeometric p<4.8×10^−6^) and peroxisome organization and biogenesis (hypergeometric p<5.5×10^−6^), nine of which we considered for further analysis (see Methods). Lirnet suggests two regulators: expression level of *PIP*2 (alias: *OAF2*), a gene that encodes a Zn(2)-Cys(6) TF that heterodimerizes with Oaf1 to regulate genes involved in peroxisomal functions via an ORE element [28], and a genetic region between nucleotides 51,324 and 52,943 on chromosome 1. Of the 11 genes in this region of chromosome 1 (Figure 6B; Table S8), Lirnet selected polymorphisms within *OAF1* as having the highest regulatory potential; the regulatory potential value of *OAF1* is within the top 1% over all genes (Figure S2). Several forms of data support the role of the Oaf1/Pip2 heterodimer in regulating this module. Of the nine target genes analyzed in the module, six contained the canonical ORE motif (p = 1.8×10^−6^). Moreover, five were in the top 1% of most significantly down-regulated genes in a microarray experiment that compared RNA expression levels of an *oaf1Δ* versus a wild-type (BY) strain under inducing conditions [29] (p = 8.0×10^−9^). We note that the *PIP2* promoter itself is Oaf1-dependent and contains an ORE element [29]. Thus, differences in *PIP2* expression patterns across the 112 segregants are also likely to be partly due to polymorphisms in *OAF1*. However, the fact that PIP2 expression was selected in addition to the *OAF1* genotype demonstrates Lirnet's ability to identify multiple relevant regulators, even when they are correlated. We note that *OAF1* was not identified by previous methods analyzing this data set.

The BY allele of *OAF1* contains two non-conservative coding polymorphisms at conserved positions that are likely to alter its function: an R70W polymorphism in the DNA binding domain (the highest-scoring SNP) and a Q447P polymorphism in the ligand binding domain [30]. *OAF1*'s high regulatory score (Figure 6B) is a combination of the correlation between its expression and genotype (cis-regulation), shared GO process annotations with the target genes, the presence of non-synonymous coding mutations and their effects on protein properties (e.g., pKa and pI), and (to a lesser extent) its function as a transcriptional regulator. Importantly, the ChIP-chip data set [24] used in our analysis did not contain Oaf1 binding information and therefore did not influence the choice of the regulator, demonstrating Lirnet's effectiveness even when binding data are not available. Moreover, another gene in the same region of chromosome 1 (*PEX22*) shares common functional annotations with many of the target genes, yet received a significantly lower score. This highlights the fact that functional annotations, although a useful source of prior knowledge, are not the primary cue used by Lirnet. Both of these characteristics are likely to play an important role in the analysis of data from other organisms, where binding data and functional annotations are both limited.

### Post-Translational Network

**Figure 7:**
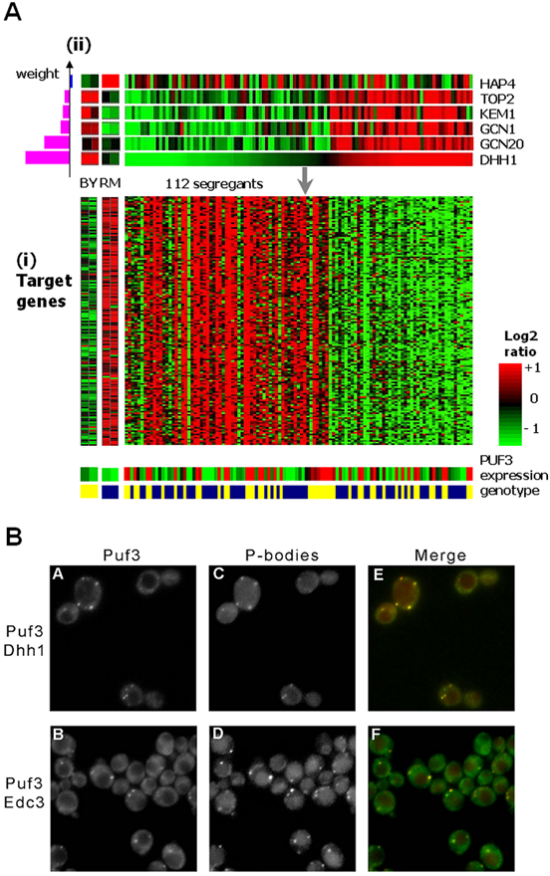
The Puf3 module. (A) A module of 153 target genes (i), which is strongly enriched for targets of the mRNA-binding protein Puf3 (shown on right, p<10^−130^; Figure S3), but neither the expression profile nor the genotype of Puf3 (shown on bottom: BY = blue, RM = yellow) are correlated with the module expression profile. (ii) The Lirnet regulatory program: the most significant predicted regulator is P-body component *DHH1*, but the regulatory program also contains P-body component Kem1, as well as translational regulators Gcn1/Gcn20. (B) Localization of Puf3 to P-bodies. Images of live cells containing a Puf3-GFP fusion and the P-body components Dhh1 or Edc3 fused to the red fluorescent protein tdimer2 (td2) (A) Puf3-GFP; (C) Dhh1-td2; (E) merged image; (B) Puf3-GFP; (D) Edc3-td2; (F) merged image. Strains containing only the Puf3-GFP fusion protein, i.e. no labeled P-body protein, formed similar fluorescent spots under the same environmental conditions (Figure S5). When present in the same cells, punctate spots of Puf3-GFP fluorescence significantly overlap with the punctate pattern formed by known P-body components (Table S10).

Lirnet also suggested an intriguing hypothesis regarding a cascaded pathway involving two modules. The first (hereafter the “Puf3 module”) contains 153 co-expressed genes, highly enriched (p<10^−91^) for nuclear genes with mitochondrial functions, and very highly enriched (p<10^−130^, Figure S3A) for genes whose mRNA transcripts are bound by the sequence-specific RNA-binding protein, Puf3 [31]. This enrichment is specific to Puf3 binding and not just coincident with the preponderance of mitochondrial genes (Figure S3B). Also, the Puf3 targets that are in the module show higher Puf3 motif score than those not (Figure S3C; Text S1). Indeed, RNA expression levels in a *puf3*Δ mutant in rich medium (YPD) showed a significant up-regulation (p<10^−37^) mRNA levels of the module genes (Figure S3D). While these results suggested that the highly coherent expression profile of this module was due, at least in part, to regulation of RNA stability via Puf3, we found that neither *PUF3* mRNA expression nor its genotype is correlated with expression of the module genes (Figures 7A, S4), suggesting that Puf3 itself was not the regulator driving the observed variability across the strains.

The Lirnet analysis identified several genes as being involved in the regulatory program of the Puf3 module (Figure 7A). Towards the top of the list, we find *DHH1* (ranked 1^st^) *and KEM1* (ranked 4^th^), two components of the dynamic cellular structures called cytoplasmic processing bodies (P-bodies) [32]–[35]. P-bodies are sites of RNA storage [32] that can modulate mRNA translation or degradation: RNA transcripts are translationally silenced while stored in the P-body [36] and can be subsequently degraded or released back into the translating pool [37]. P-bodies contain the catalytic subunits of the mRNA de-capping enzyme Dcp1/Dcp2 [38],[39], whose activity is regulated by Dhh1. However, the signals for determining mRNA localization to P-bodies and subsequent degradation or release have not been identified [32]. Thus, Lirnet suggested an intriguing regulatory connection between the Puf3-bound transcripts and a known posttranscriptional regulatory complex (P-bodies).

If Puf3 serves as a regulatory signal in one or more of the processes associated with P-bodies (RNA targeting, degradation, or release back into the translating pool), we would expect Puf3 protein to be localized to P-bodies. We therefore used fluorescence microscopy to test the subcellular localization of Puf3 in wild-type BY cells. Indeed, under certain conditions (see Methods), a Puf3-GFP fusion protein formed bright punctuate spots in the cytoplasm which co-localized with those of known P-body components, Dhh1 and Edc3 (Figure 7B). These results are consistent with those of a previous study [31] that reported punctuate cytoplasmic Puf3-GFP fluorescence in the BY strain background, but did not test for co-localization with P-bodies.

**Figure 8:**
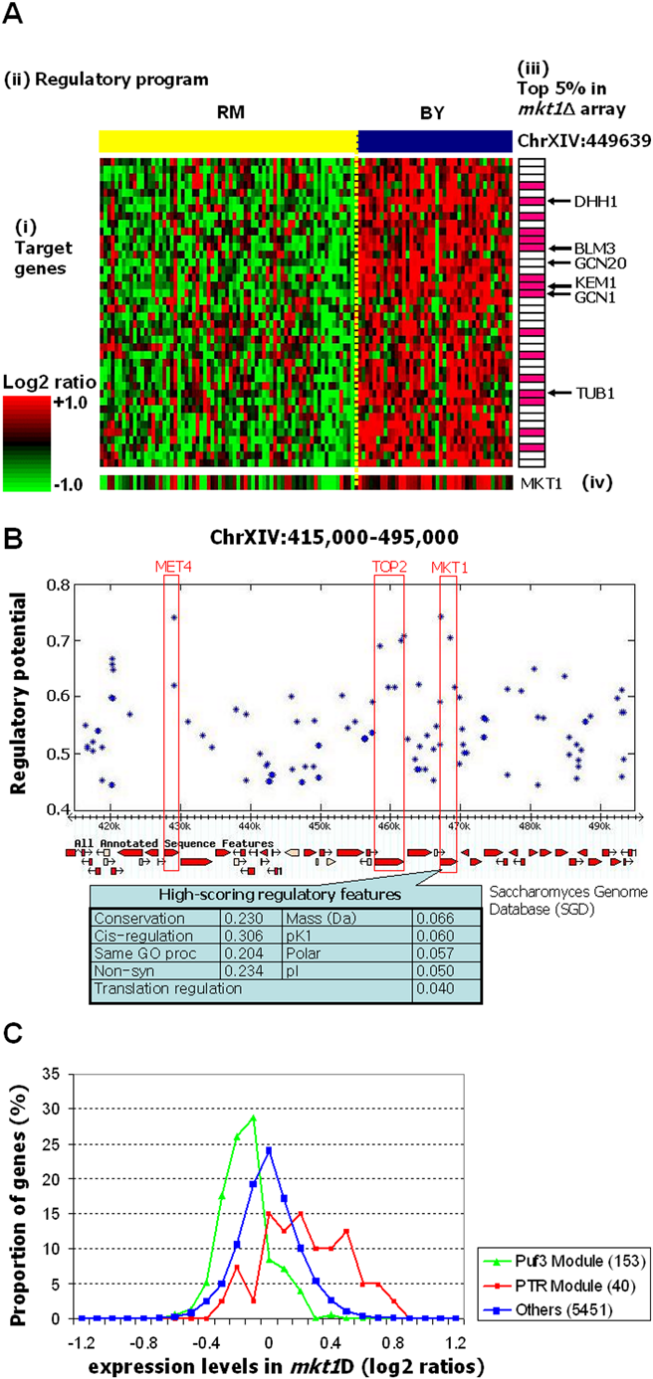
The post-transcriptional regulation (PTR) module. (A) A module of 40 target genes and its regulatory program, consisting of a genotype marker on Chromosome XIV. The module is strongly enriched for genes involved in post-transcriptional regulation processes (Figure S6), and contains many of the regulators of the Puf3 module, including P-body components Dhh1 and Kem1, and both components of the Gcn1/Gcn20 complex that regulates translation under conditions of nutrient starvation. The module's only predicted regulator is at 449,639 on Chromosome XIV. (i) The mRNA expression profiles (log_2_ ratios) of the 40 module target genes, where the rows are genes and the columns are arrays (segregants), sorted by the genotype of the segregants in the linked region on Chr XIV (shown in (ii)). (iii) Annotation of the 16 module members that are in the top 5% of genes up-regulated in the *mkt1*Δ array in an RM background (hypergeometric p<10^−10^). (iv) Expression profile of *MKT1* in the original arrays; *MKT1* was not included in our original analysis, as it did not meet our stringent cutoff for variation in expression values. (B) Of the 30 genes in the chromosome XIV region selected as the module's regulator, the highest regulatory potential is obtained by *MKT1* (Table S9). Also shown are the regulatory features that contributed the most to the selection of a SNP in Mkt1 as the causal polymorphism: conservation, linkage to the adjacent chromosomal marker (cis-regulation), common GO process annotation with target genes, the presence of non-synonymous coding mutations and their effect on properties of the resulting protein, and to a lesser extent being annotated as regulating translation. (C) RNA expression levels of an *mkt1*Δ in an RM background. Expression-value distribution for the Puf3 Module target genes (green), the PTR Module target genes (red), and the remaining genes (dark blue). The results show a modest (average fold change 0.9) but consistent down-regulation of the Puf3 Module (KS p-value<10^−23^) and up-regulation of the PTR Module (KS p-value<10^−6^).

This finding demonstrates the role of p-bodies in the regulation of the Puf3 module genes, but does not elucidate the causal SNP responsible for the difference between strains. To identify this SNP, we explore the Lirnet predictions for the regulatory program determining P-body expression. Dhh1 and Kem1 are themselves members of another module that we call the post-transcriptional regulatory (PTR) module (Figure 8A). This module also contains other regulators of the Puf3 module, including *GCN1* and *GCN20*, two members of a complex that regulates translational repression in response to nutrient starvation. Many other module members are associated, directly or indirectly, with post-transcriptional regulation (Figure S6). The sole regulator of the PTR module is a genotype marker located on Chromosome XIV (Figure 8A), the same region that had previously shown as linked to several of these targets and members of the Puf3 module [1],[40]. The smallest region linked to the PTR module spans more than 30 genes (Figure 8B; Table S9), making a systematic evaluation of the candidates a significant effort, even in a genetically tractable organism like yeast. We therefore used Lirnet to evaluate the genes in the region based on their regulatory potential for the PTR module. As shown in Figure 8B, *MKT1* is ranked as the highest scoring gene in terms of the learned regulatory potential. The regulatory potential of *MKT1* is within the top 1% over all genes (Figure S2).

Mkt1 interacts with the Poly(A)-binding protein associated factor, Pbp1, and is present at the 3′ end of RNA transcripts during translation [13]. Mkt1 contains a highly-conserved nuclease domain, homologous to the human XPG endonuclease [13] and is required for the translational regulation of an Mpt5/Puf5-dependent transcript (HO) [13]. In the BY strain background, *MKT1* harbors two non-synonymous coding mutations (Figure S7). The first variation lies in its putative nuclease domain and makes a non-conservative amino acid change (G30D) in a residue highly conserved in yeast. Both this and the more conservative mutation (R453K) are outside a Pbp1 interacting domain, which maps to the C-terminus of the protein (residues 601–760) [13]. *MKT1* has thus far been implicated in four diverse processes, including propagation of a double stranded RNA virus [41], growth at high temperature [42], efficiency of sporulation [43], and gene-specific translational regulation [13]. In many of these cases the BY variant, and, where examined, the G30D mutation specifically, appears to introduce a loss of function mutation [41],[43],[44]. Both these SNPs contributed significantly to *MKT1*'s high regulatory potential, with the G30D SNP scoring somewhat higher. The properties that contributed the most to this selection are conservation, cis-regulation, the amino-acid properties of the coding SNP, and the common GO process with the targets. These properties are precisely the ones that an expert biologist would look for in a manual scan of the region; but Lirnet automatically learned the significance of these features and their relative importance, allowing it to correctly identify the correct polymorphism using a fully automated approach. Moreover, we note that the region contains a number of other plausible candidate genes, including transcription factors and a number of mitochondrial genes; nevertheless, Lirnet identified Mtk1 as top ranked.

To test the effect of the loss of Mkt1 function on the PTR and Puf3 module genes, we deleted the *MKT1* open reading frame in the RM background and measured genome-wide RNA expression by DNA microarray analysis. Consistent with Lirnet's predictions, we observed a modest but highly significant down-regulation (KS p-value<10^−23^) of the Puf3 module in the RM *mkt1Δ* strain (Figure 8C). While previous work demonstrated Mkt1's role in repressing the translation of an Mpt5/Puf5-dependent transcript [13], our results suggest that Mkt1 plays a role in the RNA stability of Puf3-dependent transcripts. In the PTR module, 16 of 40 genes were among the top 5% most up-regulated genes (hypergeometric p<10^−10^; Figure 8A), a set which includes the Puf3 module regulators *DHH1*, *KEM1*, *GCN1*, and *GCN20*, with expression changes (1.36 fold increase) similar to the difference between RM and BY. A similar response of the Puf3 and PTR module genes was observed in an RM strain harboring the BY allele of *MKT1* (Figure S8), further supporting the role of *MKT1* in the RNA expression differences seen in the original population. Taken together, these results provide strong evidence that *MKT1* contains a causative variation for these modules, and further demonstrate Lirnet's ability to identify the correct causal regulator even in a large linked region

Thus, the Lirnet procedure automatically uncovered a comprehensive 3 tiered regulatory cascade in which *MKT1* regulates P-body abundance, that consequently regulate Puf3 target abundance, providing significant detail and insight into the mechanism through which the Puf3 module is regulated (Figure S9). Other methods [8],[11] recently applied to these data produced no hypothesis regarding this pathway. The analysis of Brem and Kruglyak [3] linked these genes and many others to a region on Chromosome XIV (Figure S10), but no causal mutation was identified. Geronemo picked up Dhh1 as a key regulator but failed to identify the causal SNP or gene involved (Figure S11). The recent analysis of Zhu et al. [9], performed in parallel to our work, also identified the same genetic region, and provided experimental support for its causal role with respect to a large group of genes. However, their identification of *MKT1* as the quantitative trait gene was manual, and based on biological intuition, rather than an automated method. Moreover, their method failed to elucidate the mechanism through which *MKT1* regulates the gene expression, missing the role of both Puf3 and the P-bodies. Thus Lirnet is unique in its ability to automatically generate a comprehensive regulatory pathway from causal SNP via the intermediate regulator through which this SNP acts upon the linked genes (Figure S9).

## Discussion

Advances in technology, most notably the emerging availability of inexpensive sequencing, are likely to give rise to the production of large amounts of data measuring both genotype and expression across large cohorts of individuals, both in human and in model organisms. These data provide a unique potential to elucidate the biological mechanisms underlying complex traits, including both basic biological functions and traits related to human health, such as predisposition to disease or response to treatment. However, our ability to unravel complex traits depends not only on the availability of data, but also on our ability to construct more sophisticated models of the complex pathways underlying these traits, and to identify the polymorphisms that perturb them. The precise identification of specific causal polymorphisms is critical for understanding the mechanisms underlying disease, and for constructing targeted diagnostic tests and treatments.

Lirnet provides a unified method that tackles these two inter-related problems: constructing a regulatory network from eQTL data, and learning the extent to which different regulators and sequence variations are likely to play a causal role in modifying expression data. Like other methods that allow for combinatorial regulation, Lirnet provides the potential for uncovering multiple factors underlying complex traits. The use of carefully regularized linear regression allows Lirnet to construct high-quality, biologically plausible regulation programs. Our results demonstrate that many of the regulatory programs inferred by Lirnet have significant support in data sets not used for constructing the network. The key novel component in the Lirnet method is its ability to learn a model of the regulatory potential of individual SNPs and genes, which estimates how likely they are to play a causal role in gene expression. This capability serves two important roles: it allows us to exploit prior knowledge in constructing better regulatory networks, by selecting regulators that are more likely to play a causal role; and it allows us to select a particular polymorphism within a large linked region as the most likely causal regulator.

Other methods have been proposed that address one or both of these goals. A number of methods make use of prior knowledge in constructing regulatory networks. The pre-determined selection of candidate regulators [7],[14] is a form of prior knowledge on the set of regulators. Other methods prioritize the choice of regulatory program using pairwise relationships between TFs and their targets, based on ChIP-chip data or on binding site data [9],[45]. Various types of prior knowledge has also been used for selecting a causal gene within a linked region, including: correlation of expression between regulator and targets [8], [9], [46]–[48], TF binding data [9], or paths in a protein-protein interaction networks [10],[11].

Several important differences distinguish Lirnet from these previous approaches. First, Lirnet avoids the use of special-purpose methods and hand-selected parameters for utilizing different types of prior knowledge. Rather, it automatically learns the regulatory potential from data, allowing it to utilize any set of regulatory features that appear relevant in a given organism and data set, without additional engineering effort. Our results comparing to two state-of-the-art methods [9],[10] demonstrate that the Lirnet method, with its automatically learned priors, provides significantly better reconstructions of regulatory interactions and better ability to identify the causal polymorphism. At a more qualitative level, Lirnet's ability to flexibly accommodate new types of features will allow it to utilize different types of high-throughput functional data. Second, Lirnet is able to make use of sequence features, such as conservation or significance of the sequence change, in a deeper way than simply eliminating all candidate genes without polymorphisms in the coding sequence [46]; as we saw, this property allows the method to be used in less well-characterized organisms, such as human, where functional data, such as transcription-factor binding or functional characterization, are scarce. The use of sequence-based features allows Lirnet to identify not only the gene that induces the expression change, but also particular sequence polymorphisms within the gene that underlie the functional change. This property is critical in obtaining a mechanistic understanding of the perturbation underlying the phenotype. Lirnet's ability to identify the causal regulator, and even the specific SNP, is likely to be even more valuable in higher-level organisms, where linked regions are long and contain many polymorphisms, and where experiments to test different candidate hypotheses are far more difficult.

There are several limitations to our work that provide directions for further developments. First, we have exploited only a basic set of regulatory features; it is likely that improved results can be obtained with a richer set of regulatory features [49]. In particular, a deeper study of the effect of different sequence features, including, for example, synonymous SNPs, may give rise to insights about the effect of different sequence perturbations. Moreover, additional data sets that indicate regulatory interactions continue to be produced, and can be usefully adopted as regulatory features. In particular, all of the data sets that we used to produce our benchmark set of regulator-target interactions (such as differential expression subject to regulator deletion or over-expression) can also be used as meta-features, as can other high-throughput data such as signaling interactions [50],[51] or genetic interactions [52],[53]. The flexibility of Lirnet allows these features to be easily integrated into the model. More broadly, Lirnet currently utilizes prior knowledge only regarding regulators and regulator-target interactions. We often have data relating to relationships between targets (such as protein-protein interactions), and even between regulators (such as cooperative or competitive binding). It would be interesting to extend the method to exploit such relationships.

One exciting opportunity is the application of the concept of a regulatory potential to the task of identifying the causal polymorphisms underlying phenotypes of interest, such as disease or drug response. In particular, it seems plausible that a sequence variation that is more likely to be causal relative to gene expression traits may also have a higher chance of playing a causal role for other phenotypes. If so, then a regulatory potential learned from eQTL data can help narrow down hypotheses in association or linkage studies. This capability could be of value in several settings: in reducing the burden of multiple hypothesis testing by favoring hypotheses that are more likely to be causal [54],[55]; in identifying plausible regions for resequencing or for follow-up in a larger population; and in prioritizing particular polymorphisms that may be worthy of follow-on experiments. This idea may allow eQTL data from model organisms to be used to increase the power in human disease studies, where expression data from relevant tissues is not readily available.

## Methods

### Dataset and Regulators

We applied our analysis to the expression and the genotype data generated from 112 meiotic recombinant progeny of two yeast strains: BY4716 (BY; a laboratory strain) and RM11-1a (RM; a natural isolate) [3]. Our expression data and genotype data were selected as previously described [7]. Our 305 candidate expression regulators were selected using the process previously described [7], and are listed in our accompanying website (http://dags.stanford.edu/lirnet/).

We applied Lirnet to the eQTL dataset [4],[56] of human HapMap individuals −60 European (CEU) and 60 African (YRI) individuals. Among 47,297 probes in the expression data [4], we picked 7,324 whose standard deviation is greater than 0.03 and used them for our analysis. The phase II HapMap data [56] contain genotypes for nearly 4 million SNPs. To perform our experiment in a setting that is closer to that of a real association study, we selected only the SNPs that are on a commercial genotyping chip, namely Affymetrix GeneChip 100 k & 500 k, and used only their genotypes in our analysis.

### Identifying Single Nucleotide Polymorphisms (SNPs) between BY and RM

We first identified orthologous genes between BY and RM. We downloaded the genome sequences of S288C (isogenic to BY) and RM from the *Saccharomyces* Genome Database (http://www.yeastgenome.org/) and Broad Institute of Fungal Genome Initiative (http://www.broad.mit.edu), respectively (sequences were retrieved on 12 January 2005). In order to define orthologous genes between BY and RM, we used reciprocal best BLAST hit [57] (protein sequences of S288C were downloaded from SGD on 12 January 2005). Out of 6,683 genes in 16 nuclear chromosomes, 6,292 (94.1493%) have reciprocal best matches between the two strains. We also retrieved the genomic sequences, 500 bp upstream/downstream of each orthologous pair. We aligned the DNA sequences of the ortholog pair by using LAGAN [58], and retrieved SNPs between the orthologs.

### Regulatory Features for SNPs

We constructed a set of *regulatory features* that describes each single nucleotide variation (SNP) in terms of various intrinsic characteristics. We identified orthologs between BY and RM, and constructed a list of SNPs, as described above. For human regulatory features, we downloaded data from dbSNP containing a list of human SNPs and their various properties. For each SNP, we defined six kinds of features that can determine its regulatory potential. First, we characterized each SNP in terms of its location relative to genes, resulting in seven regulatory features (1 & 12–17 in Table S1). Each non-synonymous coding SNP can change various properties of the corresponding amino acid (AA), which can affect the regulatory role of its gene. Therefore, we described each non-synonymous coding SNP in 10 ways in terms of changes in various properties caused by the corresponding AA change based on various data sources [59],[60] (2–11 in Table S1). A sequence polymorphism on the genomic site that is strongly conserved is more likely to affect the regulatory network. Thus, we characterized each SNP in terms of the conservation score on its genomic site (18 in Table S1). The conservation score was computed based on comparison of protein sequences across a large group of species. For yeast data, we downloaded the aligned sequences from Wapinski et al. [61]. For human data, we downloaded the conservation scores from the UCSC human genome browser (“Most Conserved” track). We also incorporated the feature indicating whether the SNP is likely to regulate the expression of the gene in which it resides (19 in Table S1).

Because regulatory potential of a SNP is likely to be affected by the function of the gene where it resides, we defined a set of regulatory features that indicate whether the gene belongs to each of 87 Gene Ontology (GO) categories related to regulatory roles (20 in Table S1). For human data, we used 48 GO Slim biological process categories. Finally, a SNP might have different regulatory potential over different modules. We defined three ‘pair-wise features’ that describe how likely a SNP is to regulate a particular module (21–23 in Table S1). For each module, we picked GO categories – biological process and molecular function – that are significantly enriched in the module genes; and transcription factors (TFs) whose putative targets appear significantly in the module, based on the ChIP-chip binding data [24]. More precisely, we picked the GO categories and TFs whose hypergeometric p-value is below 10^−3^ after a false discovery rate (FDR) correction. Then, for a combination of a SNP and a module, we constructed three features based on whether: (1) the gene containing the SNP belongs to the module's GO process; (2) the gene containing the SNP belongs to the module's GO function; and (3) the SNP resides in the module's TF. In all cases, we took -log(p-value) to be the value of the regulatory feature, so that a regulator-module pair where the enrichment is highly significantly will have a higher-valued regulatory feature.

Overall, for each SNP *n*, this process results in *a* set of 22 regulatory features and 87 (for yeast)/ 48 (for human) features based on the gene function, listed in Table S1.

### Regulatory Potentials for Regions (G-Regulators)

Based on the regulatory features of each individual SNP, we modeled the *regulatory potential* of each genetic marker, representing how likely sequence variations on the marker's chromosomal region regulate expression levels of genes. We also defined a regulatory potential for each e-regulator, representing how likely the regulator's expression is to regulate other genes' expression.

These potentials are based on the regulatory potential of individual SNPs. We model the probability that each SNP *n* causes expression variation (regulatory potential of *n*) as:
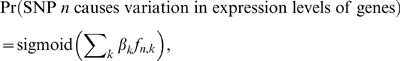
(1)

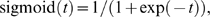
where *β_k_* is the parameter called *regulatory prior* that determines the impact of each regulatory feature on the regulatory potential: higher values of *β_k_* encode the fact that the presence of the feature *f_nk_* increases the probability of having a regulatory effect. The learning algorithm of Lirnet automatically estimates the value of the *β* parameters from data. In our analysis, we focus only on regulatory features that are likely to increase the regulatory potential, and hence restrict *β_k_* to be non-negative; this assumption can easily be relaxed in the context of other feature sets. A SNP with many important regulatory features (with high *β*'s) will have a higher regulatory prior, but the sigmoid function introduces a saturation effect, preventing the regulatory potential from increasing unboundedly and swamping the data.

Due to linkage disequilibrium, each marker *i* can represent genotypes of the chromosomal region where it resides. We therefore define the regulatory potential of each genetic marker as an aggregate of the regulatory potentials of the individual SNPs in the corresponding chromosomal region. We assigned each SNP to the region associated with its nearest genotyped marker (or tag SNP). Then, for each region *r*, we aggregated the contributions of all SNPs (in the region), each modeled based on (1), by summing them up and taking a sigmoid function:
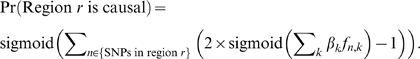
(2)


Therefore, a region that contains a number of SNPs with high regulatory potentials is likely to have a high regulatory potential, but the outer-most sigmoid function again prevents it from increasing unboundedly. We note that a region that contains a large number of moderately relevant SNPs can also achieve a high regulatory potential. This method of aggregation tends to prefer regions with more SNPs, which is arguably justified, as they are also more likely to contain a causal polymorphism. However, other methods of aggregation are also plausible. We experimented with several other approaches; the one selected achieved the highest performance in prediction of expression profiles in test data not used for training the model.

### Regulatory Potentials for Expression Regulators

We also model the regulatory potential of candidate expression regulators based on their regulatory features. We used the regulatory features of SNPs (Table S1) for constructing those of an expression regulator. The regulatory features consist of five categories: (1) 7 features each representing the number of SNPs in the gene region having one of the features 1 & 12–17 in Table S1; (2) 1 feature representing the conservation score of the gene region (analogous to 18 in Table S1); (3) 1 binary feature indicating whether the gene is cis-regulated (analogous to 19 in Table S1); (4) 87 (for yeast)/ 48 (for human) binary features indicating whether the gene belongs to each of the GO categories listed in Table S12 (analogous to 20 in Table S1); and (5) three pairwise binary features indicating whether the gene belongs to a GO process category enriched in the module, whether the gene belongs to a GO function category enriched in the module and whether the gene is the TF whose putative binding targets are enriched in the module (analogous to 21–23 in Table S1).

We define the regulatory potential of *r* to be the probability that each candidate e-regulator *r* causes expression variation, which we model as follows:
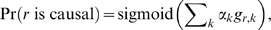
(3)where *g_rk_* represents the *k*'th regulatory feature of e-regulator *r* (explained above) and *α_k_* is the weight assigned to the *k*'th regulatory feature.

### Learning Regulatory Programs using the Lirnet Algorithm

Lirnet attempts to reconstruct regulatory programs that define the regulatory interactions between each group of co-regulated genes (called a *module*) and its regulatory factors (regulators). Candidate regulators of a module consist of binary genotype values of genetic markers and expression levels of genes that are not in the module. We model the expression level of each gene *g* in a module *m* (denoted by *y_m,g_*) as a linear combination of the potential regulators (denoted by *x_1_*,…,*x_n_*):
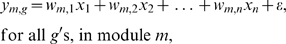
(4)where *ε* represents a zero mean Gaussian noise, and ***x*** and ***y*** are standardized.

Our objective is to estimate the weights (*w_m,1_*,…,*w_m,n_*) for each module *m*, from the data that best reflect the regulatory relationship between *x*'s and *y*. More precisely, given *x* and *y*, we aim to construct the network by maximizing the joint log-likelihood *Log P(* ***w*** *,* ***y*** *|* ***x*** *) = Log P(* ***y*** *|* ***x*** *,* ***w*** *)+Log P(* ***w*** *)*, where for each module and its regulators *P(* ***y*** *|* ***x*** *,* ***w*** *)∼Ν(Σ_i_w_i_x_i_,σ^2^)* and *P(* ***w*** *)* represents the prior probability distribution of ***w***. We model the prior probabilities on the weights based on the regulator's regulatory potential: For a regulator *r*, which can be either a region or an e-regulator, the prior probability is modeled as:
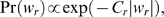
(5)

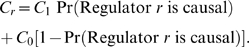



The regulatory potentials, Pr(Regulator *r* is causal), are defined in (2) and (3) as functions of ***β*** and ***α***, respectively. C_0_ and C_1_ represent the maximum and minimum regularization parameters C_i_, respectively. The prior on the weight *w_r_* is an L_1_ prior, which tends to move the weights of less relevant coefficients to 0 [16]; the larger *C_r_*, the stronger the bias towards 0. As the regulatory potential increases, we have that *C_r_* decreases, reducing the tendency of the learning algorithm to set the regulator's coefficient to 0.

To avoid the singularity problem (when the number of parameters is greater than the number of data instances) and a degeneracy problem that occurs when some of the *x*'s are correlated, we introduced an additional *L_2_ regularization term* (also called a *ridge term*) with a regularization parameter *D*. The Lirnet algorithm estimates ***α***, ***β***, and ***w*** by solving the following optimization problem:
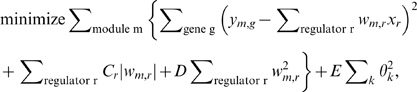
(6)where *C_r_*'s are defined in (5), θ = {α}∪{β}.

Unlike previous approaches that use tree regression [7],[62] or stepwise linear regression [48], this approach deals well with correlated regulators. In particular, when several regulators are highly correlated, both tree regression and stepwise linear regression will select one representative within the set, often arbitrarily; with that regulator selected, the correlated regulators have little explanatory power, and will not be added to the regulator set. This approach is susceptible to making arbitrary decisions based on random fluctuations in the data.

Lirnet uses an iterative coordinate descent algorithm to minimize the above objective function (Figure 1), iterating over two steps, where in one step we optimize over *w*'s given the current α and β, and in the other step we optimize over α and β given the current *w*'s. To learn θ, we solved the optimization problem of Eq. (6) with the current weights *w*'s. To estimate *w*'s, we modified least angle regression (LARS) [63] to allow it to handle the L_2_ regularization term (the third term in (6)); LARS is known to be one of the most efficient algorithms for solving this type of regularized regression problem. The regularization parameters C_0_, C_1_, *D* and *E* were determined through a 10 fold cross validation procedure: The arrays were divided into 10 groups; in each run, we train on 9/10 of the arrays, and compute predictive accuracy on the held out 1/10; the parameters were selected to maximize the average performance over the 10 runs. The final set of regulators was determined by using the chosen D and E over the entire set of arrays.

Lirnet can be used both to construct a regulatory program for a pre-determined set of modules, or, as a step in an iterative procedure whereby modules are adapted dynamically to match the learned regulatory programs. In this iterative process, first developed in the module networks algorithm [14], one starts with an initial assignment of genes to modules, for which regulatory programs are subsequently learned. Each gene is then reassigned to the module whose current regulatory program best explains its expression profile. We ran Lirnet both on a set of Geronemo modules, and using this iterative process initialized from these modules. The PGV analysis (see below) showed essentially no difference between these two runs (data not shown). To facilitate comparison to the previous results, we therefore used the Lirnet program for the Geronemo modules.

### Experiments on the Human Data

We applied Lirnet to the human eQTL dataset: genotype data from Phase II HapMap Project −60 European (CEU) and 60 African (YRI) individuals [56] – and expression profiles from the same individuals [4]. We treated SNPs on the Affymetrix GeneChip Human Mapping 100 k/ 500 k Array sets as genetic markers (i.e. tagging SNPs), and assumed we observed the genotype of only those SNPs. The regulatory features were constructed for 6,515,224 SNPs downloaded from NCBI dbSNP database (http://www.ncbi.nlm.nih.gov/SNP), based on various data sources such as dbSNP, UCSC genome browser (http://www.genome.ucsc.edu/), and gene ontology [64]. The list of regulatory features for the human data can be found in Table S3. We divided the SNPs into regions corresponding to each of the tag SNP, assigning each gene to its closest tag SNP, and defined the regulatory potential of each individual SNP and each region, according to Eq (1) & (2) in Methods, similarly to the experiments on the yeast data. The learned regulatory prior is listed in Figure 2 and Table S3.

### Implementation of a Classical Method for Comparison

As a baseline method for comparison, we used the standard single-marker linkage model (as in [1]–[3]). For each (gene, marker) pair, we performed a linear regression using the gene's expression level as a response variable and the marker as a predictor, and chose the marker that achieves the best fit. For the comparison on the yeast data, we used the published results [1]. We compared those results to those of our implementation of single-marker linkage model (explained above), and the results were almost identical (data not shown). For the human data, we used our implementation.

### Calculation of Percentage of Genetic Variance (PGV)

We estimated the percentage of genetic variance (PGV) explained by the identified genetic regulators, following the procedure of Brem & Kruglyak, as also used for Geronemo [7]. In brief, we randomly divided the data of 112 segregants into a detection set (training data) and an estimation set (test data). We used the method on the detection set to learn the regulation programs and (where relevant) the modules, and used the estimation set to calculate the PGV for these regulation programs. The PGV formula uses a corrected ANOVA, which automatically accounts for model complexity determined by the number of predictors. We repeated this process 10 times with different random splits of data, and estimated PGV of each gene by taking the average of its PGV over 10 runs.

We note that, in the protocol of Brem & Kruglyak, the set of regulators is chosen on the detection set, but the actual parameters are estimated using ANOVA on the estimation set. Thus, there is a risk that more complex regulatory program will be able to overfit the training data, producing misleadingly good results. Although the number of parameters in our model is not larger than the number in the Geronemo model, we wanted to demonstrate directly that overfitting is not a factor in these results. We therefore also used an alternative PGV protocol, where the entire regulatory program – both the choice of regulators and the parameters – are derived from the detection set alone, and then the resulting model is estimated on the test set. In the results from this protocol (Figure S1), the proposed models also considerably outperformed Geronemo.

### Biological Evaluation of Learned Regulatory Programs

We constructed a set of putative regulator-target pairs for the biological evaluation of the methods. We used five kinds of datasets: (1) deletion and over-expression microarrays [21],[22]; (2) chromatin immunce-precipitation (ChIP-chip) binding experiments [23]; (3) mRNA binding pull-down experiments [31]; (4) transcription factor binding sites [65]; and (5) a literature-curated set of signaling interactions from the Proteome database (http://www.proteome.com). For (1), we considered as targets the genes whose expression changes are within the top 10% of all genes in terms of the magnitude of the expression change. For (2), for each transcription factor, we picked the genes with a p-value of p<0.01. For (3), (4) and (5), we downloaded the lists of putative targets suggested by the corresponding papers.

### Biological Evaluation of Predicted Causative SNPs in a Large Chromosomal Region

For each of 13 regions that are identified to contain many candidate regulators of expression variation [1], we constructed a list of genes that have experimental supports of their regulatory role on the targets linked to that region, based on microarray data from deletion experiments [1], [9], [21]–[23] and ChIP-chip binding data [23] (see Figure 4B legend). For each region, we checked for each candidate regulator whether the candidate is “supported” by any of these data: (1) For the deletion microarray dataset, we considered the candidate regulator to be supported if there is a significant overlap (p<0.01; hypergeometric distribution) between its putative target based on the deletion data (within top 20% of differentially expressed genes) and the targets defined by the eQTL data [1]; (2) For the ChIP-chip binding data, we considered a regulator to be supported if there is a significant overlap between the putative targets with a binding significance of p<0.01 and the linked targets.

### Ranking Genes in a Chromosomal Region

To identify the causal SNP in a chromosomal region chosen by Lirnet as a g-regulator, we ranked each SNP using its regulatory potential, computed from its regulatory features and the learned coefficients (as in Figure 2). We then ranked the genes according to the SNP of highest regulatory potential in the gene region (coding region, 500 bp upstream, 100 bp downstream).

### Yeast Strains and Media

Unless stated, all *S. cerevisiae* strains used in this study are isogenic with a *GAL2* ^+^ derivative of S288c [66] and are also isogenic to the BY parental strain [3]. Strains used to test the effect of deleting *MKT1* in the RM background we constructed by transforming a *URA3*-marked deletion allele into RM11-1a. The “allele swap” strains (RM *mkt1-by*), replacing the RM allele of *MKT1* (G30, R453) with the BY allele (D30, K453), were constructed by standard methods of PCR and transformation into the RM *mkt1*Δ*::URA3* deletion strain. The presence of the appropriate variations and absence of any secondary mutations in the substituted region was confirmed by DNA sequence analysis. All strains were constructed by standard methods of PCR amplification and yeast transformation; details are available upon request. Microarray expression analysis of *puf3*Δ and *mkt1*Δ in the BY background used strains from the homozygous yeast deletion collection [67] (Open Biosystems) with the BY4743 isogenic parental strain as a control. Microarray expression analysis of *mkt1*Δ and *mkt1-by* in the RM strain background used the isogenic parental strain RM11-1a as a control. Strains containing Puf3, Dhh1, and Edc3 GFP protein fusions were taken from the collection described by Huh et al. [68]. Strains containing inframe protein fusions to the Red fluorescent protein, tdimer2, were constructed using pKT176 [69] by standard methods of yeast methods of PCR amplification and yeast transformation into either the strains containing the GFP tagged protein or the isogenic wild-type strain BY4741. Unless stated, all strains were grown in YPD medium and harvested in mid-log phase.

### Microarray Analysis for the *mkt1Δ* Experiment

Total yeast RNA was isolated by hot phenol method [70]. For both standard and tiling array analysis (see Text S1), total RNA was converted to cDNA and labeled with Alexa 647 and Alexa 555 (Molecular Probes) using the Atlas PowerScript Fluorescent Labeling Kit (Clontech) and an oligo(dT) primer as described by the manufacturer. Labeled cDNA samples were hybridized to either a stock yeast expression array (Agilent-011445 Yeast Oligo Microarray G4140A) or a custom yeast tiling array (described below) and processed according to manufacturer's instructions (Agilent Technologies). Arrays were scanned using a ScanArray Express HT (Perkin Elmer) at a constant laser power of 90% and various photomultiplier tube gains as described in Dudley et al. (2002). Signal and background intensities were measured using GENEPIX image analysis software (Axon Instruments) and data from multiple intensity scans were combined onto a common scale using the MASLINER linear regression method [71]. The log_2_ ratio of intensities of the signal and the background was calculated for each array element, and the standard normalization techniques described in Yang *et al.* [72] were applied to the log_2_ ratio values. We used global normalization and intensity-dependent normalization by using LOWESS (locally weighted scatter-plot smoothing) [73] with parameters relevant to our experimental setting, single slides and single print tips.

### Fluorescence Microscopy

Live yeast cells containing GFP and tdimer2 fluorescently tagged proteins were visualized with a Nikon Eclipse TE2000-E inverted microscope under 100× objective with oil. GFP was detected using a FITC filter, and tdimer2 was detected using HCRed1. Images were captured using a Hamamatsu Orca-ER CCD digital camera. Image capture and analysis used Metamorph 6.3R5 and Adobe Photoshop software. P-bodies, observed as bright punctate spots in the cytoplasm of cells containing a fluorescently labeled P-body protein, form in live cells after approximately 10 minutes in water or minimal medium lacking glucose under a microscope coverslip. Under the same conditions after approximately 12 minutes, a Puf3-GFP fusion protein formed similar fluorescent spots (Figure S5), most of which overlapped the P-bodies (Table S10). When present in the same cells, punctate spots of Puf3-GFP fluorescence overlap with the punctate pattern formed by known P-body components (69/75 = 92% of P-body spots are also Puf3 spots), showing localization of Puf3 to P-bodies (Table S10).

### Analysis of the Oaf1 Module

Of the ten target genes in the peroxisome module, the proximity and orientation of one (YAL049C) suggested that its co-expression could be the result of cross hybridization to the *OAF1* probe in the original microarray data; thus, it was removed from further consideration. To evaluate the dependence of the remaining target genes on Oaf1, we examined a published microarray dataset [29] comparing RNA expression *oaf1Δ* to a wild-type (BY) strain in the presence of oleate (an inducing condition). This dataset also included an estimate of the likelihood of differential expression [74]. We sorted RNA expression levels by the log10 ratios and filtered for λ values greater than 36.23 to arrive at the top 1% (63) most significantly down regulated genes (Table S11).

### Accession Numbers

The revised RM11-1a *PUF3* DNA sequence determined by this study will be deposited in GenBank (NCBI) prior to publication. All Microarray datasets will be deposited in the GEO database prior to publication.

## Supporting Information

Figure S1 Additional PGV plots. These graphs show additional comparisons of percent genetic variation (PGV), in the format of Figure 3 in the main text on (A) the yeast data, and (B) the human HapMap data, for both CEU and YRI individuals, with 500 k tag SNPs, (C) with 100 k tag SNPs, and (D) with 100 k tag SNPs (the same protocol as in Figure 3). Each shows PGV explained by detected regulation programs for Lirnet and Lirnet without modeling the regulatory potential, as measured by an alternative protocol. In the protocol of Brem & Kruglyak (used for Figure 3 in the main text), the set of regulators is chosen on the detection set (training data), but the actual parameters are estimated using ANOVA on the estimation set (test data). Thus, there is a risk that more complex regulatory program will be able to overfit the training data, producing misleadingly good results. These graphs in (A), (B) & (C) shows PGV values computed using an alternative PGV protocol, where the entire regulatory program – both the choice of regulators and the parameters – are derived from the detection set alone, and then the resulting model is estimated on the test set. In the results of this protocol, Lirnet also outperforms Lirnet without the regulatory prior and the classical single-marker approach (for human data).
(0.1 MB TIF)

Figure S2 Distribution of the regulatory potentials of individual SNPs (A) and genes (B). (A) Using the learned regulatory prior (Figure 2), we computed the regulatory potential of all SNPs (Eq 1 in Methods) for each module with the corresponding pairwise regulatory features. The histogram shows the distribution of these values. (B) We defined the regulatory potential of a particular gene to be that of the highest regulatory potential SNP associated with that gene (see Methods). The graph shows the distribution of the gene regulatory potentials.
(0.2 MB TIF)

Figure S3 Statistical enrichment for Puf3 target mRNAs. (A) Statistical enrichment for 3,152 genes included in our analysis. The 210 Puf3 targets from Gerber et al. [31] were obtained from (http://microarray-pubs.stanford.edu/yeast_puf/). Of these, 147 were present in the set of 3,152 genes used in the Lirnet analysis used to construct the module. The *P*-value representing the significance of the overlap (108 genes) between the 153 Puf3 module genes and 147 Puf3-bound mRNA transcripts was computed based on the hypergeometric distribution. (B) Statistical enrichment within the subset of genes with mitochondrial functions. We restricted our analysis to 956 nuclear genes whose protein products function in the mitochondrion, of which 588 are present in the set of 3,152 genes used in our analysis. The *P*-value representing the significance of the overlap between the 139 Dhh1 module genes and 127 Puf3 target genes was calculated based on the hypergeometric distribution. The significant enrichment for Puf3-bound transcripts within the subset of mitochondrial genes supports the hypothesis that Puf3 binding (rather than some other feature common to a large set of mitochondrial genes) is the relevant characteristic shared between these co-expressed genes. (C) Distribution of Puf3 motif scores. The distribution of Puf3 motif scores of the 147 Puf3 targets identified by the assay of Gerber et al. [31] and used in our analysis. These 147 genes were divided into two groups: 108 genes that were members of the Puf3 module (purple) and the remaining 39 Puf3 targets (blue). Motif scores were obtained from Gerber et al. [31] who used the motif finding tool MEME (Multiple EM for Motif Elicitation) [75] to search for the Puf3 motif. The Puf3 motif is more coherent in the module genes than in the other Puf3 targets, providing further support for the assertion that our method has independently identified a group of Puf3-dependent transcripts. (D) Up-regulation of Puf3 targets in a BY *puf3*Δ. Distributions of microarray expression values in a BY puf3 deletion mutant (*puf3*Δ) are shown. The x-axis shows the log-2 ratio expression level (*puf3Δ* : wildtype), and the y-axis the percentage of genes with that expression level within the Puf3 module genes (purple) and within the set of all remaining genes (blue). The higher expression levels of the Puf3 module in *puf3*Δ are significant (p-value<10^−37^) by Kolmogorov-Smirnov test.
(0.1 MB TIF)

Figure S4 Revised protein sequence for the RM allele of *PUF3*. Orthologous genes between BY and RM were determined by reciprocal best BLAST hit [57], as previously described [7]. Although the genome sequence of the RM strain (*Saccharomyces cerevisiae* RM11-1a Sequencing Project, Broad Institute, http://www.broad.mit.edu/annotation/genome/saccharomyces_cerevisiae/Home.html) reports the presence of a series of coding mutations in *PUF3* that would effectively truncate the C-terminal portion of the protein, re-sequencing of this region of *PUF3* from the RM strain revealed only two amino acid substitution mutations in this region and an additional amino acid substitution mutation in the N-terminal region of the protein.
(0.03 MB TIF)

Figure S5 Images of live cells containing a Puf3-GFP fusion protein. Puf3-GFP forms punctuate spots under conditions required for P-body formation. GFP Fluorescence channel (FITC) and cell morphology (DIC). These strains do not contain any other fluorescently labeled proteins, and thus control for the possibility that Puf3 spots seen in the co-localization experiments are an artifact of P-body fluorescence.
(0.3 MB TIF)

Figure S6 Summary of known functional interactions between the PTR module target genes. The network was generated using data collected from the literature (Table S13), E-MAP analysis (Table S14), and RNA expression levels (Figure S12). The edges represent different functional connections, as indicated; thick lines correspond to interactions tested in small-scale experiments, thin lines to high-throughput assays. The genetic interaction edges (pink, purple) are taken from a recent E-MAP assay of 505 genes associated with various aspects of RNA metabolism (Table S14, Figure S13). The expression correlation edge (dark green) indicates very high similarity of gene expression microarray data in knockout strains *puf3Δ* and *gcn20Δ* (Figure S12, Table S15), indicating a functional connection between the deleted genes [21],[76]. We note that absence of an edge has no significance, since not all possible combinations have been tested.
(0.01 MB TIF)

Figure S7 *MKT1* polymorphisms. The aligned protein sequences of Mkt1 encoded by BY and RM, constructed as described in Lee et al. [7]. The two sequences are identical except for two SNPs: G30D and R453K. Both polymorphisms occur in residues that are highly conserved in the other yeast species shown. Also marked are three previously identified protein domains [13]: the XPG-N putative nuclease domain (pink), the XPG-I putative nuclease domain (green), and the Pbp1 binding domain (yellow). The non-conservative G30D SNP is located in the XPG-N domain.
(0.05 MB TIF)

Figure S8 RNA expression in an RM strain harboring the BY allele of *MKT1* (*mkt1-by*). Expression-value distribution for different groups of genes in RM *mkt1-by* experiment, measured by tiling array hybridization (Methods): genome wide (dark blue); Puf3 Module (green); PTR Module (red). The results show a modest but consistent down-regulation of the Puf3 Module (KS p-value<10^−13^) and up-regulation of the PTR Module (KS p-value<10^−8^). In the PTR module, we find 23 of 40 genes in the module in the top 10% of genes most up-regulated (hypergeometric p-value<10^−12^). These genes include Lirnet-predicted regulators of the Puf3 module: *DHH1* (2.3%; 114 out of 4926 verified ORFs), *KEM1* (2.8%), *GCN1* (1.1%) and other genes in the PTR modules: *BLM3* (5.8%), *TUB1* (7.8%), *ECM29* (0.6%) and *SIM1* (3.2%). The results agree well with the effects seen in a complete deletion of the *MKT1* open reading frame.
(0.02 MB TIF)

Figure S9 Overview of the three-tiered regulatory cascade proposed by our analysis. (I) A highly coherent module of 153 genes was identified; these genes are nuclear genes with mitochondrial function, of which an overwhelming majority is bound by the sequence-specific RNA-binding protein Puf3 (Figure S3A). (II) Lirnet predicted a set of regulators that suggested the regulation of these targets by two distinct post-transcriptional regulation processes: P-body factors and the Gcn1/Gcn20 complex (Figure 7A). The relationship between the Puf3 targets and P-bodies is supported by microscopy experiments. (III) Lirnet also identified a locus on chromosome XIV as linked with the expression variation in these processes, and suggested a specific gene in this region – MKT1 – as the causal polymorphism (Figure 8B). The regulatory role of MKT1 in inducing the observed variation is supported by microarray experiments.
(0.1 MB TIF)

Figure S10 Results obtained by a previous linkage based approach. Results of analysis by Yvert et al. [1], which is a purely linkage based approach. Among the 3,152 genes to which Geronemo was applied, 169 genes had been linked to a locus in chromosome XIV in the previous study [1]. Of these (i) 125 were assigned to the Puf3 module and (ii) 24 were assigned to the PTR module.
(0.3 MB TIF)

Figure S11 The Geronemo Puf3 module. The regulatory program generated by Geronemo for the Puf3 module. Although Dhh1 was selected as the top regulator, the remainder of the regulatory program appears unrelated to the module function.
(0.5 MB TIF)

Figure S12 Comparison between the expression levels in *puf3*Δ and *gcn20*Δ mutants. (A) Expression levels of *puf3Δ* and *gcn20*Δ mutant arrays for 153 Puf3 module genes (top) and the rest of the genes included in our analysis (bottom). To show the correlation between the two arrays more effectively, we sorted the genes in each group based on the sum of the expression levels in the two arrays. The scatter plot shows the expression levels of the Puf3 module genes (purple) and the other genes (blue) in *puf3*Δ (x-axis) and *gcn20*Δ (y-axis) mutant. (B) A scatter plot showing the correlation between the *puf3*Δ and *gcn20*Δ arrays both within the Puf3 module (pink) and for all other genes (blue). The overall genome-wide Pearson correlation is 0.65. Although the Puf3 module is induced in *puf3*Δ and repressed in *gcn20*Δ, there is still a correlation between the values within the module (Pearson correlation 0.5). One possible explanation is that the *puf3*Δ profile is an aggregate of two effects: a general cellular response to a disruption in its mRNA turnover and translation pathways, which is the same for both knockouts; and a direct effect of the Puf3 knockout of increasing the RNA levels of the Puf3 targets. (C) Distribution of the Pearson correlation coefficients from every pair of 300 arrays from the Rosetta yeast deletion mutant compendium of Hughes et al. [21]. Of the expression profiles resulting from different gene knockouts, only 28 of 44,850 pairs of knockouts exhibited a Pearson correlation >0.65, almost all occurring in pairs of genes that are functionally related (Table S15).
(0.2 MB TIF)

Figure S13 Distribution of the E-MAP synthetic sickness values and pairwise correlations. (A) We find significant synthetic sickness between *gcn1*Δ and deletion of P-body component *dcp1*Δ (−2.7). These values are at the top 1.91% and 1.93%, respectively, in the distribution of synthetic sickness values of all 94,680 measured pairs among the 505 genes in the E-MAP. We also found synthetic sickness relationships between *puf4*Δ, another member of the PUF family, and two deletions of genes encoding P-body components, *dcp1*Δ (−3.9, top 1.13%) and *lsm1*ΔΔ (−8.85, top 0.21%). (B) E-MAP data can also be used to measure similarity between the interaction profiles of different genes. We find strong correlations of synthetic sickness profiles between *puf3*Δ and P-body component *edc3*Δ (PCC = 0.401 – top 0.9% in PCCs of all deletion pairs; 0.245 – top 4.8%), between *puf4*Δ and P-body components *lsm1*Δ and *pat1*Δ (PCC = 0.544 – top 0.21%; PCC = 0.476 – top 0.41%), and between *gcn1*Δ and *puf3*Δ, *puf4*Δ (PCC = 0.236 – top 5.32%; PCC = 0.277 – top 3.34%).
(0.06 MB TIF)

Table S1 Regulatory features. For each single nucleotide polymorphism (SNP), we constructed a list of properties (called *regulatory features*) that can indicate how much likely the SNP causes variation in expression levels of genes. Each column contains the following information: **Name –** Name of the regulatory feature; **Property –** One of S, G and GP meaning **S**NP-specific, **G**ene-specific and **G**ene-specific **P**airwise, respectively; and **Description –** The meaning of the regulatory feature.
(0.05 MB DOC)

Table S2 Learned regulatory features for yeast. We list the learned regulatory prior for all regulatory features in the yeast data. Each column contains: **Regulatory feature** – name of the regulatory feature; and **Regulatory prior** – the learned regulatory prior.
(0.07 MB DOC)

Table S3 Learned regulatory features for human. We list the learned regulatory prior for all regulatory features in the human HapMap data (CEU & YRI). Each column contains: **Regulatory feature –** name of the regulatory feature; and **Regulatory prior –** the learned regulatory prior.
(0.08 MB DOC)

Table S4 Biological evaluation of the learned regulatory program. We constructed a set of comparison regulatory interactions from various datasets: deletion and over-expression microarrays [21],[22]; chromatin immune-precipitation (ChIP-chip) binding experiments [23]; mRNA binding pull-down experiments [31]; transcription factor binding sites [65]; and a literature-curated set of signaling interactions from the Proteome database (http://www.proteome.com/). For a prediction that a regulator R regulates a module M, we defined it to be validated if there was significant overlap (hypergeometric p<0.01) between the members of M and the putative targets of R, suggested by one of the above datasets. We note that none of these datasets was used for constructing the regulatory features for Lirnet. For each method, we counted the number of validated interactions (column named # regulators), where each entry shows: *a/b* (*c*%), where *a* is the number of significant regulators, *b* is the total number of predicted regulators that appear at least once in the reference dataset, and *c* is the proportion (*a/b*×100). We similarly counted the number of modules that have at least one validated regulator (column named #modules), relative to the total number of modules having a predicted regulator in the reference set. We also considered two-step regulatory cascades, as described in the main text (see also Methods). (A) and (B) show the number of validated regulators for expression and genetic regulators, respectively.
(0.05 MB DOC)

Table S5 Learned regulatory programs and their supports. For each module with ≥5 genes, we listed the learned regulators with their p-values. The p-values indicate the significance of overlap between the module targets and the genes that have been suggested to be targets of the regulator. We also considered two-step regulatory cascades, as described in the main text (see also Methods).
(0.05 MB XLS)

Table S6 Comparison to the method of Suthram et al. We compare to another published method [10], which improves on earlier work of Tu et al. [11]. The authors validate their results relative to a pre-defined set of 548 regulatory relationships, extracted from gene knockout or over-expression microarray studies [21],[25], similarly to our analysis. The predicted network of Suthram et al. was not available, so we evaluated Lirnet using their protocol and reference set, to allow for a direct comparison. For each target gene and its linked region, we selected the gene containing the SNP with the highest regulatory potential in that region. We then evaluated these predictions using the 548 reference pairs of Suthram et al. The result shows that Lirnet significantly outperforms both the method of Suthram et al. and the previous method of Tu et al. [11], according to this evaluation metric. The results of other methods –Random, Tu et al and eQED – are from Table 1 in Suthram et al [10].
(0.04 MB DOC)

Table S7 Composition of Zap1 region in terms of SNPs and regulatory potentials. We list all SNPs in the Zap1 region. Each column contains the following information: **SNP ID –** the SNP ID (1-n); **Gene –** name of the gene where the SNP resides (including upstream and downstream regions); **Loc –** one of U, C and D representing **U**pstream, **C**oding region and **D**ownstream, respectively; **Regpot –** learned regulatory potential of the SNP; **Chr, Pos –** chromosome, position of the SNP; **BY-Nuc –** nucleotide allele in BY, **RM-Nuc –** nucleotide allele in RM; **BY-AA –** corresponding AA in BY; and **RM-AA –** corresponding AA in RM.
(0.4 MB DOC)

Table S8 Composition of Oaf1 region in terms of SNPs and regulatory potentials. We list all SNPs in the Oaf1 region. Each column contains the following information: **SNP ID –** the SNP ID (1-n); **Gene –** name of the gene where the SNP resides (including upstream and downstream regions); **Loc –** one of U, C and D representing **U**pstream, **C**oding region and **D**ownstream, respectively; **Regpot –** learned regulatory potential of the SNP; **Chr, Pos –** chromosome, position of the SNP; **BY-Nuc –** nucleotide allele in BY, **RM-Nuc –** nucleotide allele in RM; **BY-AA –** corresponding AA in BY; and **RM-AA –** corresponding AA in RM.
(0.2 MB DOC)

Table S9 Composition of Mkt1 region in terms of SNPs and regulatory potentials. We list all SNPs in the Mkt1 region. Each column contains the following information: **SNP ID –** the SNP ID (1-n); **Gene –** name of the gene where the SNP resides (including upstream and downstream regions); **Loc –** one of U, C and D representing **U**pstream, **C**oding region and **D**ownstream, respectively; **Regpot –** learned regulatory potential of the SNP; **Chr, Pos –** chromosome, position of the SNP; **BY-Nuc –** nucleotide allele in BY, **RM-Nuc –** nucleotide allele in RM; **BY-AA –** corresponding AA in BY; and **RM-AA –** corresponding AA in RM.
(0.2 MB DOC)

Table S10 Microscopy quantitation. To quantitate the extent to which Puf3 protein (assayed by Puf3-GFP fluorescence, green) co-localized with P-bodies (assayed as Edc3-tdimer2 fluorescence, red), we counted the number of spots that exhibited visually detectable green and/or red fluorescence. The number of Puf3 spots reached a maximum at approximately 20 minutes post induction. We cannot rule out the possibility that differences in rates of spot formation could be due to differences in the properties of GFP and tdimer2 because our inability to detect Puf3 fused to tdimer2 or other versions of red fluorescent protein (Dudley and Drubin, unpublished results) prevented us from swapping the fluorescent protein tags.
(0.05 MB DOC)

Table S11 Deletion arrays. To evaluate the dependence of the remaining target genes on Oaf1, we examined a published microarray dataset [29] comparing RNA expression *oaf1Δ* to a wild-type (BY) strain in the presence of oleate (an inducing condition). This dataset also included an estimate of the likelihood of differential expression [74]. We sorted RNA expression levels by the log10 ratios and filtered for λ values greater than 36.23 to arrive at the top 1% (63) most significantly down regulated genes.
(0.1 MB DOC)

Table S12 Regulatory Gene Ontology (GO) categories. For constructing regulatory features, we characterized each gene based on GO categories. Different organisms have different sets of categories that are relevant to the regulatory processes. Therefore, we used different lists for yeast and human data. (A) We constructed a list of 76 biological process and 11 molecular function Gene Ontology (GO) categories that might be related to gene regulatory functions in yeast. (B) For human data, we used a list of 48 GO Slim biological process categories.
(0.09 MB DOC)

Table S13 Functional interactions between the PTR module members and related genes. Data and references were obtained from the Saccharomyces Genome Database (SGD).
(0.09 MB DOC)

Table S14 Genetic Interactions in the EMAP data.
(0.7 MB DOC)

Table S15 Highly correlated expression profiles in the Rosetta yeast deletion compendium. We show the significance of the correlation between the genomic expression levels of *puf3*Δ and *gcn20*Δ mutants (Pearson's correlation coefficient = 0.65; Figure S12) by comparing it with those of the pairs of arrays from Rosetta deletion mutant dataset [21]. For every pair from 300 arrays consisting of diverse mutations and chemical treatment in *S. cerevisiae*, we calculated Pearson's correlation coefficients, and present the pairs whose correlation coefficients are higher than 0.65.
(0.05 MB DOC)

Text S1 Supplementary Methods.
(0.08 MB DOC)
