# Supplementary figures and images for: Learning a Prior on Regulatory Potential from eQTL Data
Source: PLoS Genet. 2009 Jan 30;5(1):e1000358. doi: 10.1371/journal.pgen.1000358 (PMC2627940; doi:10.1371/journal.pgen.1000358)

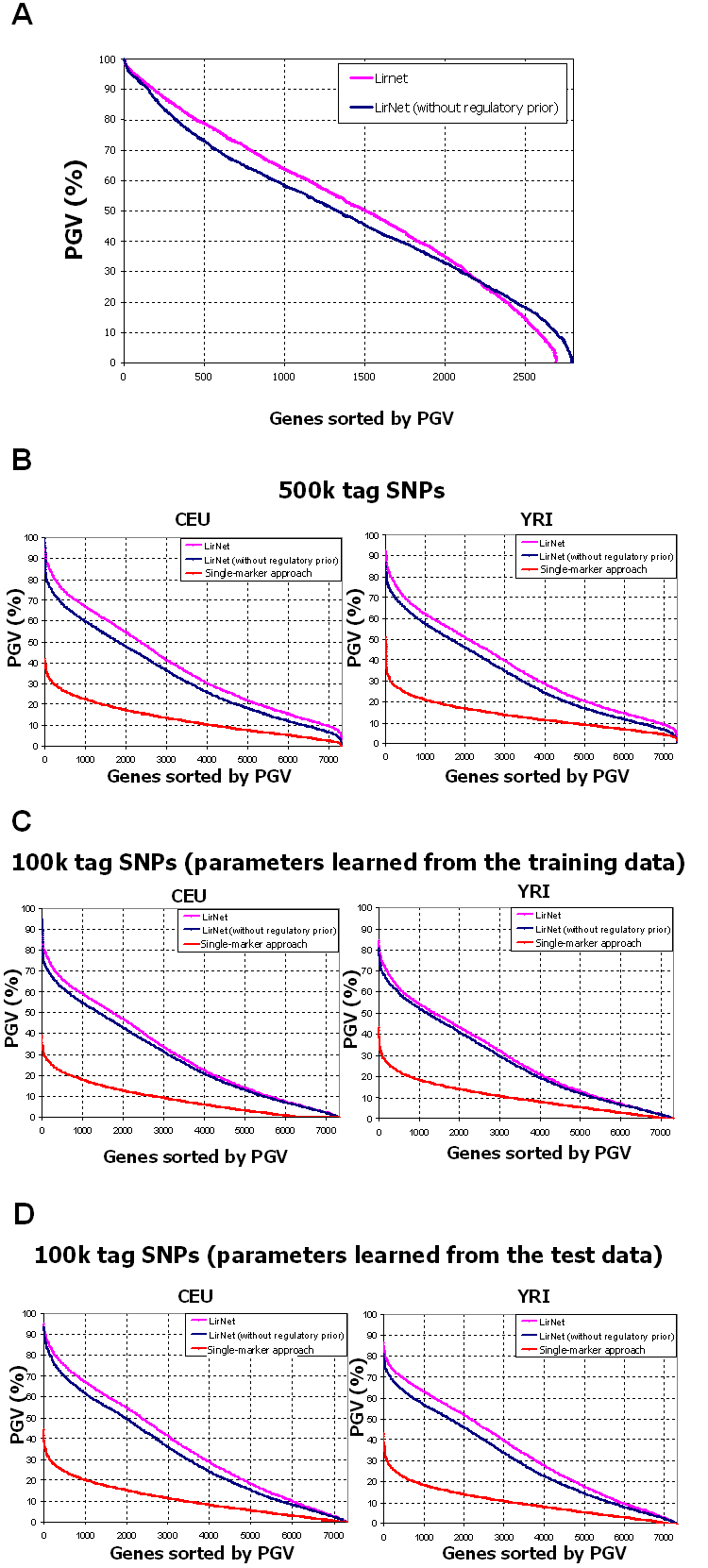

Supplement: Figure S1 — Additional PGV plots. These graphs show additional comparisons of percent genetic variation (PGV), in the format of Figure 3 in the main text on (A) the yeast data, and (B) the human HapMap data, for both CEU and YRI individuals, with 500 k tag SNPs, (C) with 100 k tag SNPs, and (D) with 100 k tag SNPs (the same protocol as in Figure 3). Each shows PGV explained by detected regulation programs for Lirnet and Lirnet without modeling the regulatory potential, as measured by an alternative protocol. In the protocol of Brem & Kruglyak (used for Figure 3 in the main text), the set of regulators is chosen on the detection set (training data), but the actual parameters are estimated using ANOVA on the estimation set (test data). Thus, there is a risk that more complex regulatory program will be able to overfit the training data, producing misleadingly good results. These graphs in (A), (B) & (C) shows PGV values computed using an alternative PGV protocol, where the entire regulatory program – both the choice of regulators and the parameters – are derived from the detection set alone, and then the resulting model is estimated on the test set. In the results of this protocol, Lirnet also outperforms Lirnet without the regulatory prior and the classical single-marker approach (for human data). (0.1 MB TIF) [file pgen.1000358.s001.tif]

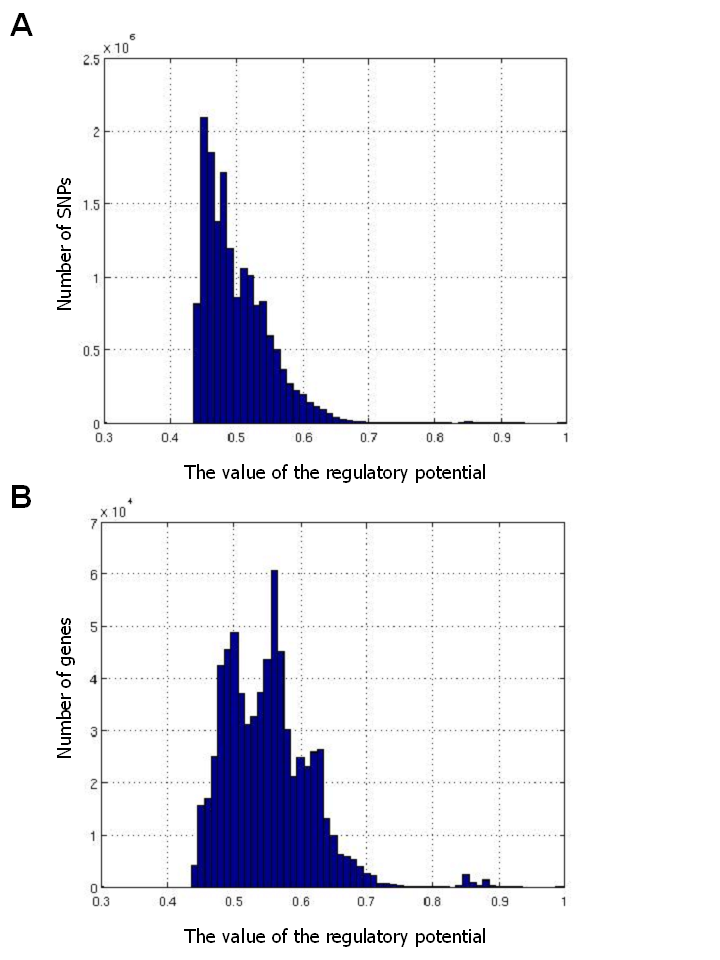

Supplement: Figure S2 — Distribution of the regulatory potentials of individual SNPs (A) and genes (B). (A) Using the learned regulatory prior (Figure 2), we computed the regulatory potential of all SNPs (Eq 1 in Methods) for each module with the corresponding pairwise regulatory features. The histogram shows the distribution of these values. (B) We defined the regulatory potential of a particular gene to be that of the highest regulatory potential SNP associated with that gene (see Methods). The graph shows the distribution of the gene regulatory potentials. (0.2 MB TIF) [file pgen.1000358.s002.tif]

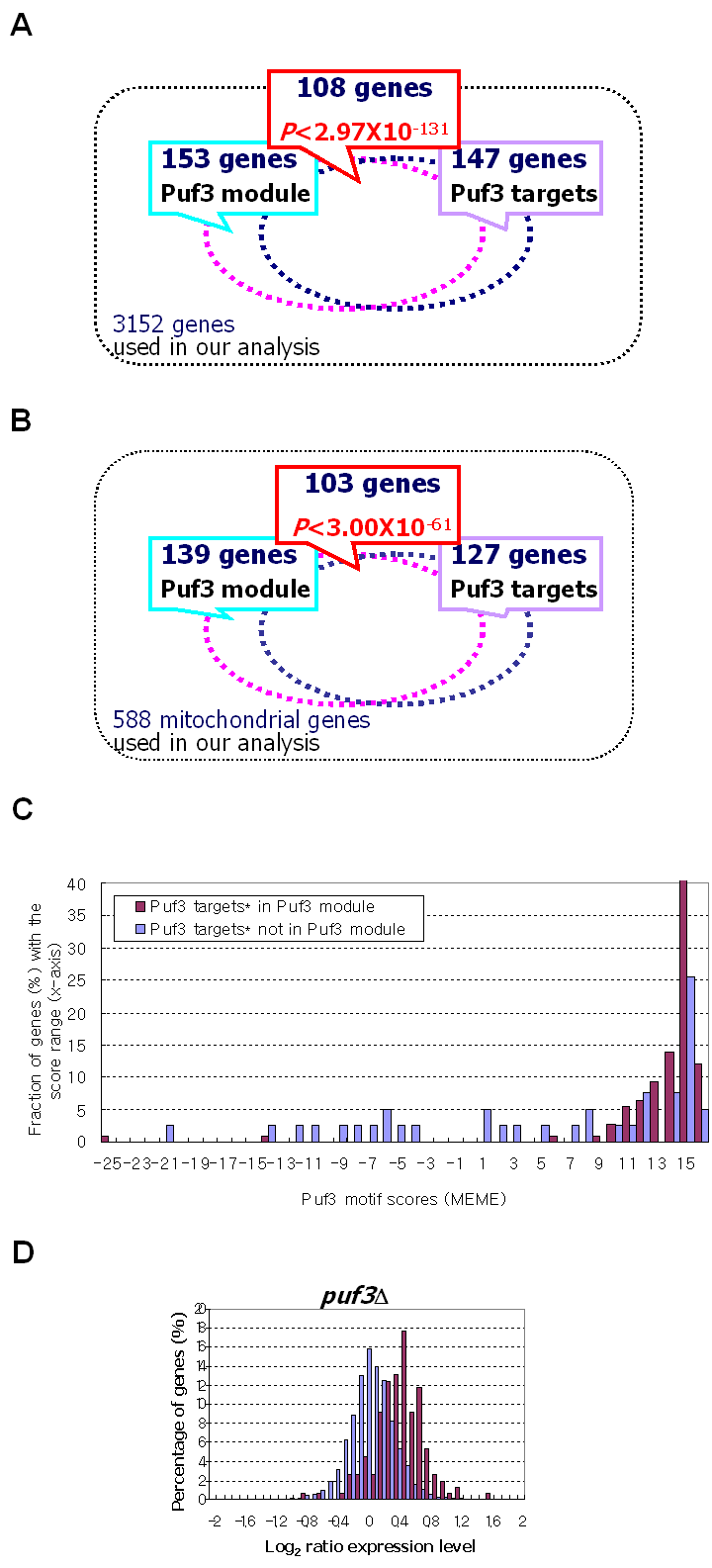

Supplement: Figure S3 — Statistical enrichment for Puf3 target mRNAs. (A) Statistical enrichment for 3,152 genes included in our analysis. The 210 Puf3 targets from Gerber et al. [31] were obtained from (http://microarray-pubs.stanford.edu/yeast_puf/). Of these, 147 were present in the set of 3,152 genes used in the Lirnet analysis used to construct the module. The P-value representing the significance of the overlap (108 genes) between the 153 Puf3 module genes and 147 Puf3-bound mRNA transcripts was computed based on the hypergeometric distribution. (B) Statistical enrichment within the subset of genes with mitochondrial functions. We restricted our analysis to 956 nuclear genes whose protein products function in the mitochondrion, of which 588 are present in the set of 3,152 genes used in our analysis. The P-value representing the significance of the overlap between the 139 Dhh1 module genes and 127 Puf3 target genes was calculated based on the hypergeometric distribution. The significant enrichment for Puf3-bound transcripts within the subset of mitochondrial genes supports the hypothesis that Puf3 binding (rather than some other feature common to a large set of mitochondrial genes) is the relevant characteristic shared between these co-expressed genes. (C) Distribution of Puf3 motif scores. The distribution of Puf3 motif scores of the 147 Puf3 targets identified by the assay of Gerber et al. [31] and used in our analysis. These 147 genes were divided into two groups: 108 genes that were members of the Puf3 module (purple) and the remaining 39 Puf3 targets (blue). Motif scores were obtained from Gerber et al. [31] who used the motif finding tool MEME (Multiple EM for Motif Elicitation) [75] to search for the Puf3 motif. The Puf3 motif is more coherent in the module genes than in the other Puf3 targets, providing further support for the assertion that our method has independently identified a group of Puf3-dependent transcripts. (D) Up-regulation of Puf3 targets in a BY puf3Δ. Distribut [file pgen.1000358.s003.tif]

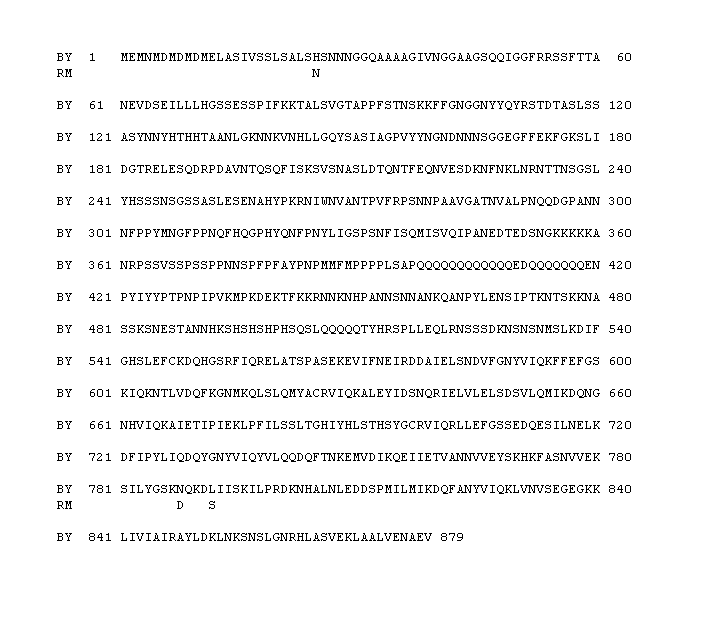

Supplement: Figure S4 — Revised protein sequence for the RM allele of PUF3. Orthologous genes between BY and RM were determined by reciprocal best BLAST hit [57], as previously described [7]. Although the genome sequence of the RM strain (Saccharomyces cerevisiae RM11-1a Sequencing Project, Broad Institute, http://www.broad.mit.edu/annotation/genome/saccharomyces_cerevisiae/Home.html) reports the presence of a series of coding mutations in PUF3 that would effectively truncate the C-terminal portion of the protein, re-sequencing of this region of PUF3 from the RM strain revealed only two amino acid substitution mutations in this region and an additional amino acid substitution mutation in the N-terminal region of the protein. (0.03 MB TIF) [file pgen.1000358.s004.tif]

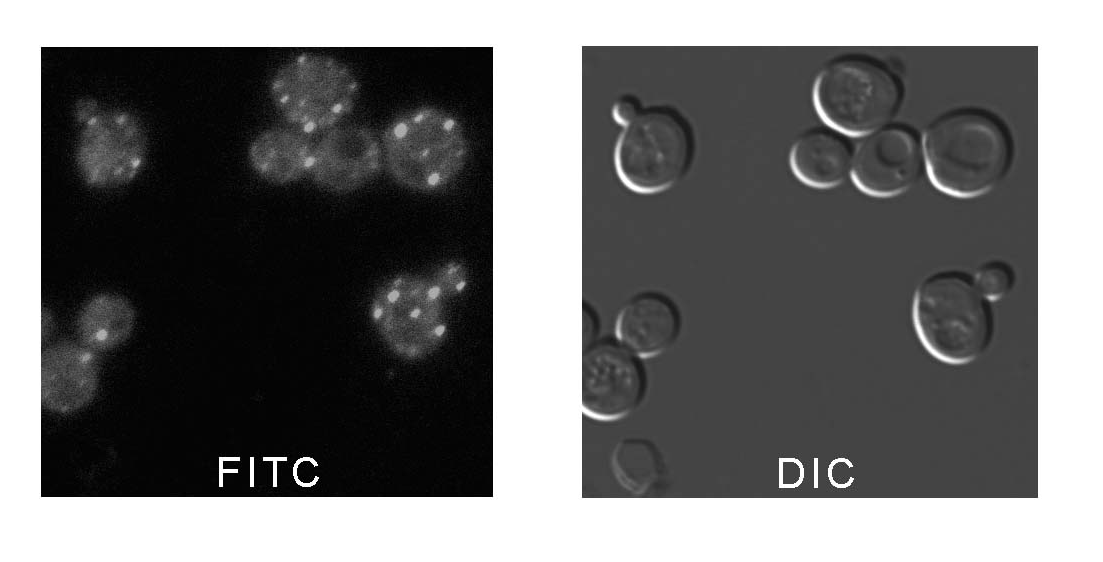

Supplement: Figure S5 — Images of live cells containing a Puf3-GFP fusion protein. Puf3-GFP forms punctuate spots under conditions required for P-body formation. GFP Fluorescence channel (FITC) and cell morphology (DIC). These strains do not contain any other fluorescently labeled proteins, and thus control for the possibility that Puf3 spots seen in the co-localization experiments are an artifact of P-body fluorescence. (0.3 MB TIF) [file pgen.1000358.s005.tif]

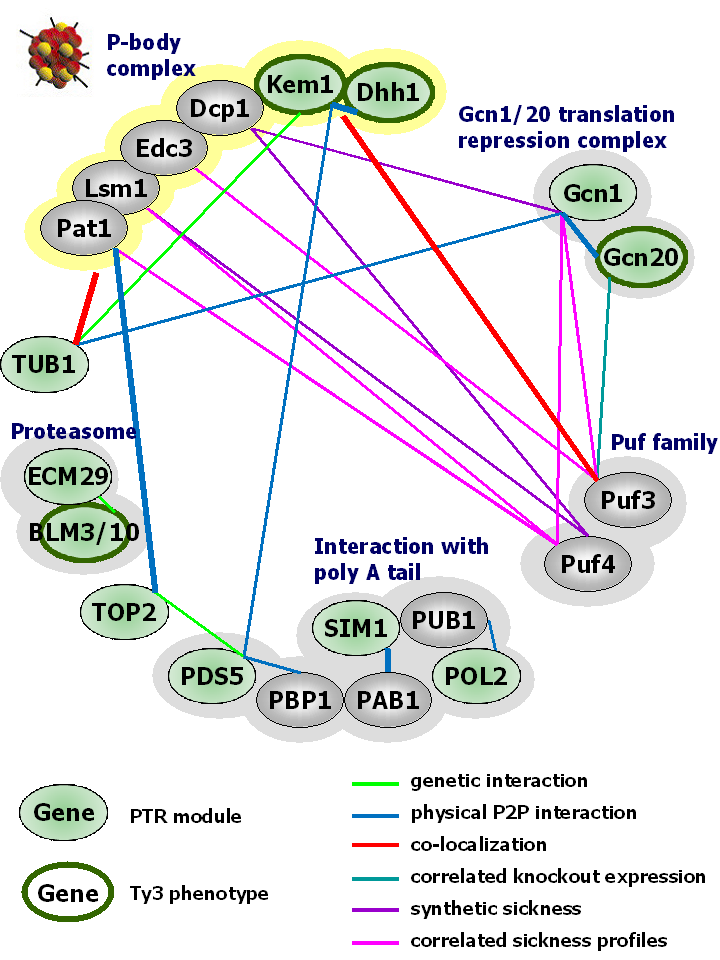

Supplement: Figure S6 — Summary of known functional interactions between the PTR module target genes. The network was generated using data collected from the literature (Table S13), E-MAP analysis (Table S14), and RNA expression levels (Figure S12). The edges represent different functional connections, as indicated; thick lines correspond to interactions tested in small-scale experiments, thin lines to high-throughput assays. The genetic interaction edges (pink, purple) are taken from a recent E-MAP assay of 505 genes associated with various aspects of RNA metabolism (Table S14, Figure S13). The expression correlation edge (dark green) indicates very high similarity of gene expression microarray data in knockout strains puf3Δ and gcn20Δ (Figure S12, Table S15), indicating a functional connection between the deleted genes [21],[76]. We note that absence of an edge has no significance, since not all possible combinations have been tested. (0.01 MB TIF) [file pgen.1000358.s006.tif]

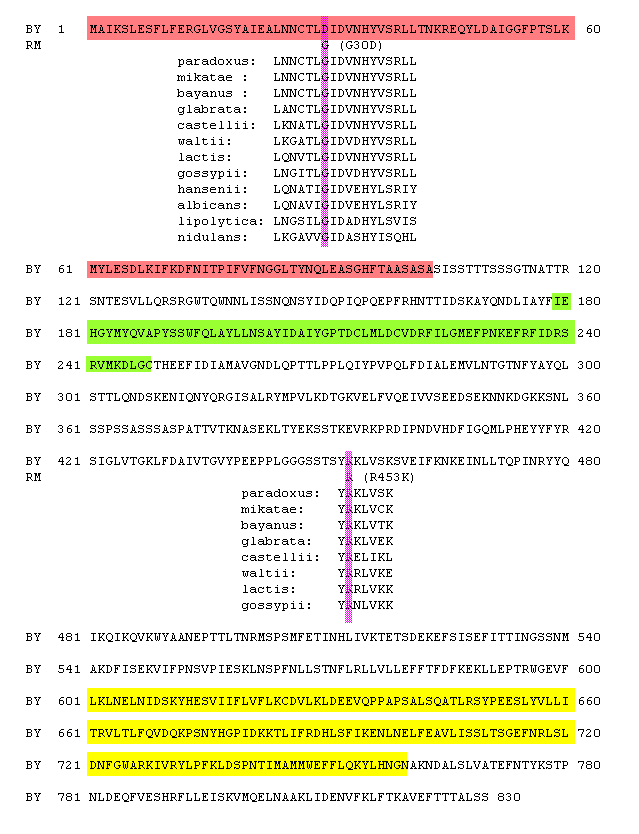

Supplement: Figure S7 — MKT1 polymorphisms. The aligned protein sequences of Mkt1 encoded by BY and RM, constructed as described in Lee et al. [7]. The two sequences are identical except for two SNPs: G30D and R453K. Both polymorphisms occur in residues that are highly conserved in the other yeast species shown. Also marked are three previously identified protein domains [13]: the XPG-N putative nuclease domain (pink), the XPG-I putative nuclease domain (green), and the Pbp1 binding domain (yellow). The non-conservative G30D SNP is located in the XPG-N domain. (0.05 MB TIF) [file pgen.1000358.s007.tif]

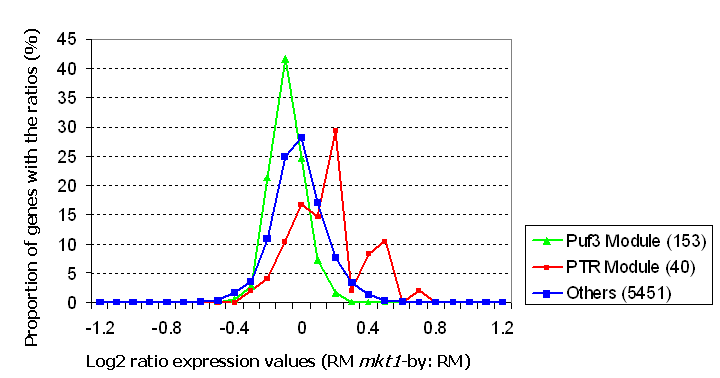

Supplement: Figure S8 — RNA expression in an RM strain harboring the BY allele of MKT1 (mkt1-by). Expression-value distribution for different groups of genes in RM mkt1-by experiment, measured by tiling array hybridization (Methods): genome wide (dark blue); Puf3 Module (green); PTR Module (red). The results show a modest but consistent down-regulation of the Puf3 Module (KS p-value<10−13) and up-regulation of the PTR Module (KS p-value<10−8). In the PTR module, we find 23 of 40 genes in the module in the top 10% of genes most up-regulated (hypergeometric p-value<10−12). These genes include Lirnet-predicted regulators of the Puf3 module: DHH1 (2.3%; 114 out of 4926 verified ORFs), KEM1 (2.8%), GCN1 (1.1%) and other genes in the PTR modules: BLM3 (5.8%), TUB1 (7.8%), ECM29 (0.6%) and SIM1 (3.2%). The results agree well with the effects seen in a complete deletion of the MKT1 open reading frame. (0.02 MB TIF) [file pgen.1000358.s008.tif]

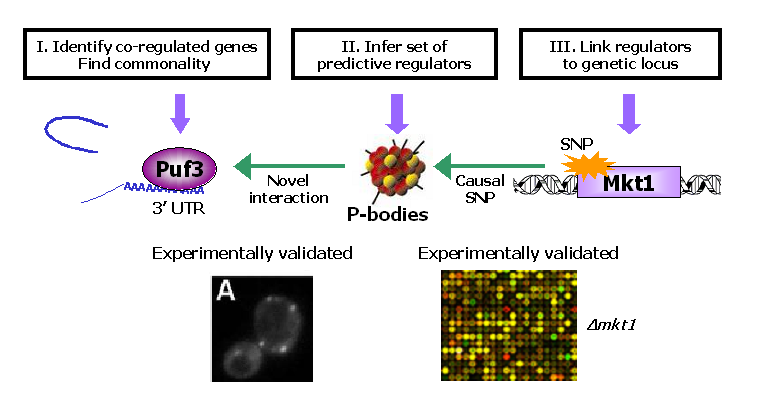

Supplement: Figure S9 — Overview of the three-tiered regulatory cascade proposed by our analysis. (I) A highly coherent module of 153 genes was identified; these genes are nuclear genes with mitochondrial function, of which an overwhelming majority is bound by the sequence-specific RNA-binding protein Puf3 (Figure S3A). (II) Lirnet predicted a set of regulators that suggested the regulation of these targets by two distinct post-transcriptional regulation processes: P-body factors and the Gcn1/Gcn20 complex (Figure 7A). The relationship between the Puf3 targets and P-bodies is supported by microscopy experiments. (III) Lirnet also identified a locus on chromosome XIV as linked with the expression variation in these processes, and suggested a specific gene in this region – MKT1 – as the causal polymorphism (Figure 8B). The regulatory role of MKT1 in inducing the observed variation is supported by microarray experiments. (0.1 MB TIF) [file pgen.1000358.s009.tif]

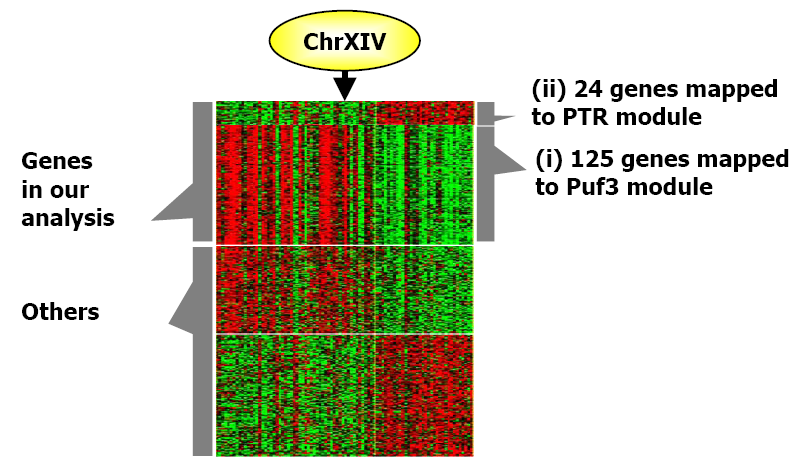

Supplement: Figure S10 — Results obtained by a previous linkage based approach. Results of analysis by Yvert et al. [1], which is a purely linkage based approach. Among the 3,152 genes to which Geronemo was applied, 169 genes had been linked to a locus in chromosome XIV in the previous study [1]. Of these (i) 125 were assigned to the Puf3 module and (ii) 24 were assigned to the PTR module. (0.3 MB TIF) [file pgen.1000358.s010.tif]

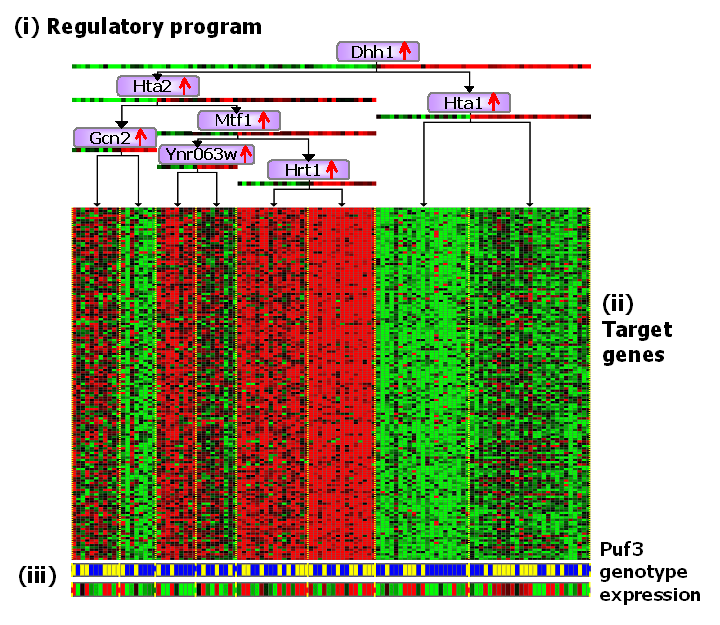

Supplement: Figure S11 — The Geronemo Puf3 module. The regulatory program generated by Geronemo for the Puf3 module. Although Dhh1 was selected as the top regulator, the remainder of the regulatory program appears unrelated to the module function. (0.5 MB TIF) [file pgen.1000358.s011.tif]

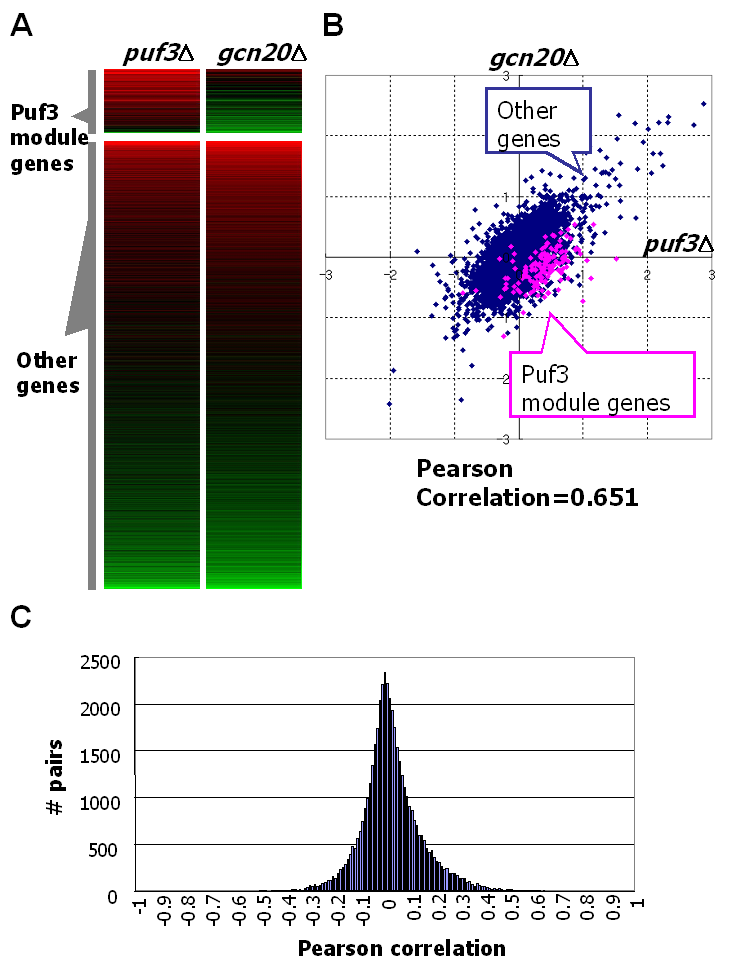

Supplement: Figure S12 — Comparison between the expression levels in puf3Δ and gcn20Δ mutants. (A) Expression levels of puf3Δ and gcn20Δ mutant arrays for 153 Puf3 module genes (top) and the rest of the genes included in our analysis (bottom). To show the correlation between the two arrays more effectively, we sorted the genes in each group based on the sum of the expression levels in the two arrays. The scatter plot shows the expression levels of the Puf3 module genes (purple) and the other genes (blue) in puf3Δ (x-axis) and gcn20Δ (y-axis) mutant. (B) A scatter plot showing the correlation between the puf3Δ and gcn20Δ arrays both within the Puf3 module (pink) and for all other genes (blue). The overall genome-wide Pearson correlation is 0.65. Although the Puf3 module is induced in puf3Δ and repressed in gcn20Δ, there is still a correlation between the values within the module (Pearson correlation 0.5). One possible explanation is that the puf3Δ profile is an aggregate of two effects: a general cellular response to a disruption in its mRNA turnover and translation pathways, which is the same for both knockouts; and a direct effect of the Puf3 knockout of increasing the RNA levels of the Puf3 targets. (C) Distribution of the Pearson correlation coefficients from every pair of 300 arrays from the Rosetta yeast deletion mutant compendium of Hughes et al. [21]. Of the expression profiles resulting from different gene knockouts, only 28 of 44,850 pairs of knockouts exhibited a Pearson correlation >0.65, almost all occurring in pairs of genes that are functionally related (Table S15). (0.2 MB TIF) [file pgen.1000358.s012.tif]

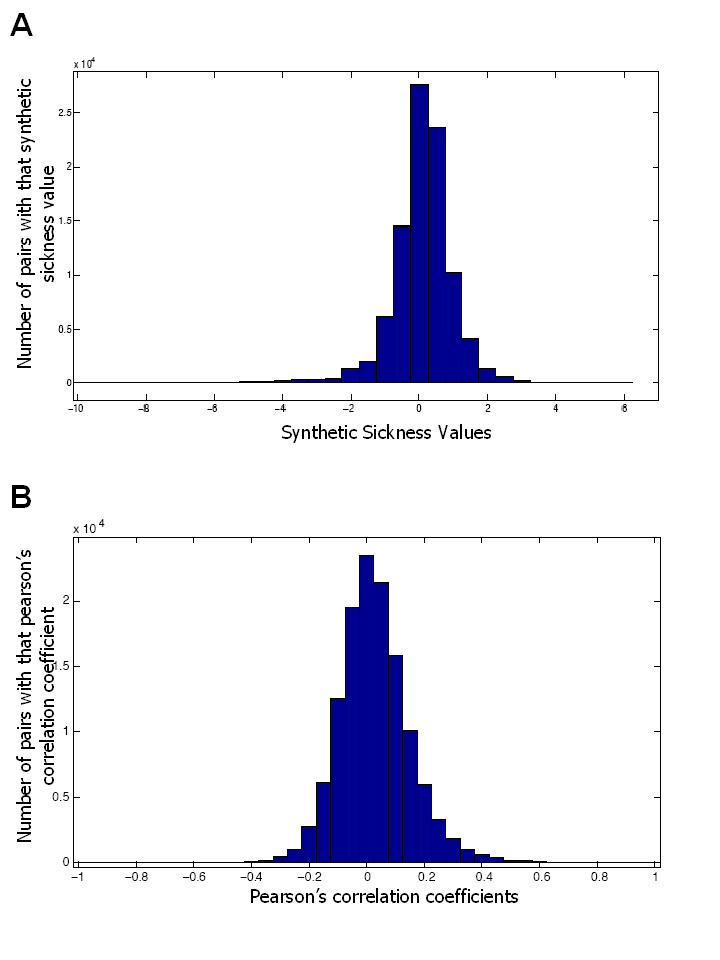

Supplement: Figure S13 — Distribution of the E-MAP synthetic sickness values and pairwise correlations. (A) We find significant synthetic sickness between gcn1Δ and deletion of P-body component dcp1Δ (−2.7). These values are at the top 1.91% and 1.93%, respectively, in the distribution of synthetic sickness values of all 94,680 measured pairs among the 505 genes in the E-MAP. We also found synthetic sickness relationships between puf4Δ, another member of the PUF family, and two deletions of genes encoding P-body components, dcp1Δ (−3.9, top 1.13%) and lsm1ΔΔ (−8.85, top 0.21%). (B) E-MAP data can also be used to measure similarity between the interaction profiles of different genes. We find strong correlations of synthetic sickness profiles between puf3Δ and P-body component edc3Δ (PCC = 0.401 – top 0.9% in PCCs of all deletion pairs; 0.245 – top 4.8%), between puf4Δ and P-body components lsm1Δ and pat1Δ (PCC = 0.544 – top 0.21%; PCC = 0.476 – top 0.41%), and between gcn1Δ and puf3Δ, puf4Δ (PCC = 0.236 – top 5.32%; PCC = 0.277 – top 3.34%). (0.06 MB TIF) [file pgen.1000358.s013.tif]
