# Supplementary material for: Learning a Prior on Regulatory Potential from eQTL Data
Source: PLoS Genet. 2009 Jan 30;5(1):e1000358. doi: 10.1371/journal.pgen.1000358 (PMC2627940; doi:10.1371/journal.pgen.1000358)
Supplement: Table S1 — Regulatory features. For each single nucleotide polymorphism (SNP), we constructed a list of properties (called regulatory features) that can indicate how much likely the SNP causes variation in expression levels of genes. Each column contains the following information: Name – Name of the regulatory feature; Property – One of S, G and GP meaning SNP-specific, Gene-specific and Gene-specific Pairwise, respectively; and Description – The meaning of the regulatory feature. (0.05 MB DOC) [file pgen.1000358.s014.doc]

| **Name** | Pro-perty | Description |
| --- | --- | --- |
| 1.Non-synonymous coding | S | A binary feature that indicates whether the SNP causes an amino acid change. |
| Sub-categories of Non-synonymous coding | S | These features characterize each non-synonymous coding SNP in 10 ways in terms of changes in various properties caused by the corresponding amino acid change.  2. Average mass (Da)  3. Isoelectric point (denoted by pI): the pH at which a particular molecule or surface carries no net electrical charge.  4. Dissociation constant (denoted by pK1)  5. Dissociation constant (denoted by pK2)  6. Hydro-phobicity: the physical property of a molecule that is repelled from a mass of water.  7. Acid dissociation constant (denoted by pKa): an equilibrium constant for the dissociation of a weak acid.  8. Polarity: the dipole-dipole intermolecular forces between the slightly positively-charged end of one molecule to the negative end of another or the same molecule.  9. Acidity (denoted by pH): a measure of the acidity or alkalinity of a solution.  10. Van der Waals volume: the radius of an imaginary hard sphere which can be used to model the atom for many purposes.  11. Essentiality: an essential amino acid cannot be synthesized in humans and must be supplied in the diet. |
| 12. Stop codon | S | A binary feature indicating whether it introduces a stop codon. |
| 13. Synonymous coding | S | A binary feature indicating whether the SNP does not cause an amino acid change. |
| 14. 3’ UTR | S | A binary feature indicating whether the SNP resides in the 3’ UTR region. |
| 15. 5’ UTR | S | A binary feature indicating whether the SNP resides in the 5’ UTR region. |
| 16. Upstream | S | A binary feature indicating whether the SNP is located in the region 500bp upstream of the transcription start site. |
| 17. Downstream | S | A binary feature indicating whether the SNP is located in the region 500bp downstream of a gene. |
| 18. Conservation score | S | A real-valued feature representing how well the SNP site is conserved across different strains (or species). |
| 19. Cis-regulation | G | A binary feature indicating whether the SNP is significantly indicative of the expression level of the gene in which it resides (t-test p-value of < 10^-4^ ). |
| 20. Gene function | G | A binary feature indicating whether the SNP resides in the gene that is known to belong to each of 87 gene ontology (GO) categories that are related to regulatory functions. For human data, we considered 48 GO Slim process categories (Tables S12) |
| 21. Pairwise GO process | GP | A binary pairwise feature defined for a combination of a SNP and a module, indicating whether the gene containing the SNP belongs to a GO biological process category that is enriched in the module. |
| 22. Pairwise GO function | GP | A binary pairwise feature defined for a combination of a SNP and a module, indicating whether the gene containing the SNP belongs to a GO molecular function category that is enriched in the module. |
| 23.Pairwise binding | GP | A binary pairwise feature defined for a combination of a SNP and a module, indicating whether the module is enriched for putative binding targets of the TF containing the SNP. |
