# Supplementary material for: Learning a Prior on Regulatory Potential from eQTL Data
Source: PLoS Genet. 2009 Jan 30;5(1):e1000358. doi: 10.1371/journal.pgen.1000358 (PMC2627940; doi:10.1371/journal.pgen.1000358)
Supplement: Table S2 — Learned regulatory features for yeast. We list the learned regulatory prior for all regulatory features in the yeast data. Each column contains: Regulatory feature – name of the regulatory feature; and Regulatory prior – the learned regulatory prior. (0.07 MB DOC) [file pgen.1000358.s015.doc]

| **Regulatory feature** | **Regulatory prior** | **Regulatory feature** | **Regulatory prior** |
| --- | --- | --- | --- |
| Non-synonymous coding | 0.233917 | mRNA metabolic process | 0 |
| Stop codon | 0.43893 | Nitrogen compound catabolic process | 0.00343187 |
| Synonymous coding | 0.258139 | Nitrogen compound metabolic process | 0 |
| 3' UTR | 0.225325 | Nuclear transport | 0 |
| 500 bp upstream | 0.112055 | Oxidative phosphorylation | 0 |
| 5' UTR | 0.194139 | Post-translational protein modification | 0.000460047 |
| 500 bp downstream | 0.146042 | Protein folding | 0.101193 |
| Conservation score | 0.28211 | Protein localization | 0.00226286 |
| Cis-regulation | 0.306031 | Protein modification process | 0.00328517 |
| Change of average mass (Da) | 0.121118 | Protein transport | 0.00553692 |
| Change of isoelectric point (pI) | 0.127865 | Protein ubiquitination | 0 |
| Change of pK1 | 0.115885 | Proteolysis | 0.0666369 |
| Change of pK2 | 0.0740295 | Pyruvate metabolic process | 0 |
| Change of hydro-phobicity | 0.0517083 | Regulation of cell cycle | 0.00253788 |
| Change of pKa | 0.095504 | Regulation of gene expression | 0.0271549 |
| Change of polarity | 0.0608224 | Regulation of gene expression, epigenetic | 0 |
| Change of pH | 0.0930487 | Regulation of metabolic process | 0.0223296 |
| Change of van der Waals volume | 0.107773 | Regulation of RNA metabolic process | 0.0291587 |
| Change of essentiality | 0.0455052 | Regulation of transcription | 0.0404949 |
| Aerobic respiration | 0 | Regulation of translation | 0.0412378 |
| Amine metabolic process | 0.00301562 | Response to DNA damage stimulus | 0.0083791 |
| Amino acid and derivative metabolic process | 0.00852627 | Response to drug | 0 |
| Aromatic compound metabolic process | 0 | Response to stimulus | 0.0164909 |
| Carbohydrate biosynthetic process | 0 | Response to stress | 0.0168409 |
| Carbohydrate metabolic process | 0.0365825 | Ribosome assembly | 0 |
| Carbohydrate transport | 0 | Ribosome biogenesis and assembly | 0 |
| Carboxylic acid metabolic process | 0.00410577 | RNA biosynthetic process | 0.0219748 |
| Cell communication | 0 | RNA metabolic process | 0.0111038 |
| Cell cycle | 0.0281754 | RNA modification | 0.0855505 |
| Cell cycle checkpoint | 0.0687474 | RNA processing | 0 |
| Cellular respiration | 0 | Secretion | 0 |
| Cell wall organization and biogenesis | 0.0312327 | Signal transduction | 0 |
| Chromatin modification | 0.00094716 | Sporulation | 0.0188201 |
| Chromatin remodeling | 0.00251332 | Sulfur metabolic process | 0.0446121 |
| Chromosome organization and biogenesis | 0.0233903 | Telomere organization and biogenesis | 0.156596 |
| Cytoskeleton organization and biogenesis | 0.0227989 | Transcription | 0.0261155 |
| Developmental process | 0.0248449 | Translation | 0.0153418 |
| DNA metabolic process | 0.0355782 | Translational initiation | 0 |
| DNA repair | 0 | Transport | 0.00991353 |
| Ergosterol metabolic process | 0 | TRNA metabolic process | 0.0120347 |
| Establishment of localization | 0.00975434 | Ubiquitin cycle | 0 |
| Glucose metabolic process | 0.100341 | ATPase activity | 0.0266334 |
| Glycolysis | 0 | DNA binding | 0.00156867 |
| Glycoprotein metabolic process | 0.00482355 | Enzyme regulator activity | 0.025876 |
| Golgi vesicle transport | 0.00225624 | Kinase activity | 0 |
| Growth | 0.0135392 | Ligase activity | 0.0153004 |
| Hexose metabolic process | 0.0761087 | Oxidoreductase activity, acting on CH-OH group of donors | 0.032485 |
| Histone modification | 0 | Protein kinase activity | 0 |
| Homeostatic process | 0 | RNA binding | 0 |
| Lipid metabolic process | 0.0201742 | Structural constituent of ribosome | 0 |
| Membrane organization and biogenesis | 0 | Transcription regulator activity | 0.181823 |
| Methionine metabolic process | 0 | Transporter activity | 0.0324857 |
| Mitochondrial transport | 0.00311074 | same GO process | 0.15827829 |
| Mitotic cell cycle | 0.0390234 | same GO function | 0.176159416 |
|  |  | ChIP-chip binding | 0.219317574 |
