# Supplementary material for: Learning a Prior on Regulatory Potential from eQTL Data
Source: PLoS Genet. 2009 Jan 30;5(1):e1000358. doi: 10.1371/journal.pgen.1000358 (PMC2627940; doi:10.1371/journal.pgen.1000358)
Supplement: Table S3 — Learned regulatory features for human. We list the learned regulatory prior for all regulatory features in the human HapMap data (CEU & YRI). Each column contains: Regulatory feature – name of the regulatory feature; and Regulatory prior – the learned regulatory prior. (0.08 MB DOC) [file pgen.1000358.s016.doc]

| **Regulatory feature** | **Regulatory prior** | | **Regulatory feature** | **Regulatory prior** | |
| --- | --- | --- | --- | --- | --- |
|  | **CEU** | **YRI** |  | **CEU** | **YRI** |
| Non-synonymous coding | 0.21135912 | 0.082092 | mitochondrion organization and biogenesis | 0.046071778 | 0.12263322 |
| Synonymous coding | 0.24747438 | 0.092838 | cytoskeleton organization and biogenesis | 0.037961892 | 0.034756709 |
| Intron | 0.05222177 | 0.008966 | cytoplasm organization and biogenesis | 0 | 0.092838516 |
| Locus region | 0.1589809 | 0.086223 | cell cycle | 0.050622536 | 0 |
| Splice site | 0.06128878 | 0.001616 | cell communication | 0.156576699 | 0.124043333 |
| UTR region | 0.16061538 | 0.076888 | signal transduction | 0.131607749 | 0.088864777 |
| Conservation score | 0.34112371 | 0.112234 | cell-cell signaling | 0.025637528 | 0.020236858 |
| Cis-regulation | 0.48054821 | 0.142857 | multicellular organismal development | 0.053065513 | 0.003082568 |
| Change of average mass (Da) | 0.10913261 | 0.036195 | behavior | 0.047664024 | 0 |
| Change of isoelectric point | 0.05214288 | 0.014501 | cell recognition | 0.014495506 | 0 |
| Change of pK1 | 0.05453091 | 0.016528 | metabolic process | 0.061411352 | 0.025385809 |
| Change of pK2 | 0.02726133 | 0.016643 | cell death | 0.059187991 | 0.072111672 |
| Change of hydro-phobicity | 0.03936422 | 0.001298 | cell proliferation | 0 | 0 |
| Change of pKa | 0.05421617 | 0.012068 | catabolic process | 0.02666964 | 0.017920226 |
| Change of polarity | 0.05862236 | 0.007212 | biosynthetic process | 0.05363503 | 0.047267919 |
| Change of pH | 0.05943284 | 0.032249 | response to external stimulus | 0 | 0.008368172 |
| Change of van der Waals volume | 0.0779721 | 0.005518 | response to biotic stimulus | 0 | 0 |
| Change of essentiality | 0.02099906 | 0.002563 | response to abiotic stimulus | 0.029336555 | 0 |
| reproduction | 0.03058678 | 0.09415171 | anatomical structure morphogenesis | 0.044330009 | 0.07214781 |
| carbohydrate metabolic process | 0.05222177 | 0.01405993 | response to endogenous stimulus | 0.088046586 | 0.046198859 |
| generation of precursor metabolites and energy | 0.0491292 | 0 | embryonic development | 0.005176701 | 0.018651958 |
| electron transport | 0.02992232 | 0.12512214 | protein transport | 0 | 0 |
| nucleobase, nucleoside, nucleotide and nucleic acid metabolic process | 0.05459965 | 0.0749332 | cellular component organization and biogenesis | 0.05916011 | 0.05160599 |
| DNA metabolic process | 0.0775168 | 0.06777273 | cell growth | 0.023309642 | 0.06051799 |
| transcription | 0.10429178 | 0.12101749 | death | 0.059136927 | 0.019283571 |
| translation | 0 | 0 | protein metabolic process | 0.019139649 | 0.026201755 |
| protein modification process | 0.03492883 | 0.04017481 | cellular homeostasis | 0 | 0 |
| amino acid and derivative metabolic process | 0 | 0 | secondary metabolic process | 0.043670197 | 0.041604791 |
| lipid metabolic process | 0.03018018 | 0 | cell differentiation | 0.063479696 | 0.040297923 |
| transport | 0.05296134 | 0.03411034 | growth | 0.016843787 | 0 |
| ion transport | 0.01140242 | 0.05299528 | regulation of gene expression, epigenetic | 0 | 0 |
| response to stress | 0.0414003 | 0.05686924 | primary metabolic process | 0.065277206 | 0.131443247 |
| organelle organization and biogenesis | 0.02585983 | 0 | regulation of biological process | 0.038841095 | 0.069978507 |
