# Supplementary material for: Learning a Prior on Regulatory Potential from eQTL Data
Source: PLoS Genet. 2009 Jan 30;5(1):e1000358. doi: 10.1371/journal.pgen.1000358 (PMC2627940; doi:10.1371/journal.pgen.1000358)
Supplement: Table S4 — Biological evaluation of the learned regulatory program. We constructed a set of comparison regulatory interactions from various datasets: deletion and over-expression microarrays [21],[22]; chromatin immune-precipitation (ChIP-chip) binding experiments [23]; mRNA binding pull-down experiments [31]; transcription factor binding sites [65]; and a literature-curated set of signaling interactions from the Proteome database (http://www.proteome.com/). For a prediction that a regulator R regulates a module M, we defined it to be validated if there was significant overlap (hypergeometric p<0.01) between the members of M and the putative targets of R, suggested by one of the above datasets. We note that none of these datasets was used for constructing the regulatory features for Lirnet. For each method, we counted the number of validated interactions (column named # regulators), where each entry shows: a/b (c%), where a is the number of significant regulators, b is the total number of predicted regulators that appear at least once in the reference dataset, and c is the proportion (a/b×100). We similarly counted the number of modules that have at least one validated regulator (column named #modules), relative to the total number of modules having a predicted regulator in the reference set. We also considered two-step regulatory cascades, as described in the main text (see also Methods). (A) and (B) show the number of validated regulators for expression and genetic regulators, respectively. (0.05 MB DOC) [file pgen.1000358.s017.doc]

A. Expression regulators:

|  | Direct | | Two-Step Cascade | |
| --- | --- | --- | --- | --- |
|  | # interactions | # modules | # interactions | # modules |
| Lirnet | 23/103 (22.33%) | 19/43 (44.19%) | 79/146 (54.11%) | 39/47 (82.98%) |
| Lirnet without regulatory prior | 20/104 (19.23%) | 17/44 (38.64%) | 72/140 (51.43%) | 36/46 (78.26%) |
| Geronemo | 10/65 (15.38%) | 9/43 (20.93%) | 49/104 (47.12%) | 28/52 (53.85%) |
| Random Model | 5/74 (6.76%) | 5/49 (10.20%) | 27/101 (26.73%) | 23/51 (45.10%) |

B. Genetic marker regulators:

|  | Direct | | Two-Step Cascade | |
| --- | --- | --- | --- | --- |
|  | # interactions | # modules | # interactions | # modules |
| Lirnet | 9/20 (45.00%) | 8/19 (42.11%) | 22/27  (81.48%) | 17/22 (77.27%) |
| Lirnet without regulatory prior | 8/18 (44.44%) | 5/16 (31.25%) | 16/22 (72.73%) | 8/18 (44.44%) |
| Geronemo | 6/40 (15.00%) | 5/30 (16.67%) | 32/54 (59.26%) | 15/40 (37.50%) |
| Random Model | 4/36 (11.11%) | 4/28 (14.29%) | 12/50 (24.00%) | 7/35 (20.00%) |
