# Supplementary material for: Learning a Prior on Regulatory Potential from eQTL Data
Source: PLoS Genet. 2009 Jan 30;5(1):e1000358. doi: 10.1371/journal.pgen.1000358 (PMC2627940; doi:10.1371/journal.pgen.1000358)
Supplement: Table S6 — Comparison to the method of Suthram et al. We compare to another published method [10], which improves on earlier work of Tu et al. [11]. The authors validate their results relative to a pre-defined set of 548 regulatory relationships, extracted from gene knockout or over-expression microarray studies [21],[25], similarly to our analysis. The predicted network of Suthram et al. was not available, so we evaluated Lirnet using their protocol and reference set, to allow for a direct comparison. For each target gene and its linked region, we selected the gene containing the SNP with the highest regulatory potential in that region. We then evaluated these predictions using the 548 reference pairs of Suthram et al. The result shows that Lirnet significantly outperforms both the method of Suthram et al. and the previous method of Tu et al. [11], according to this evaluation metric. The results of other methods –Random, Tu et al and eQED – are from Table 1 in Suthram et al [10]. (0.04 MB DOC) [file pgen.1000358.s019.doc]

| Methods | Number of correctly predicted pairs (out of 548) |
| --- | --- |
| Random | 118 |
| Tu et al | 262 |
| eQED | 438 |
| Lirnet | 466 |
