# Supplementary material for: Learning a Prior on Regulatory Potential from eQTL Data
Source: PLoS Genet. 2009 Jan 30;5(1):e1000358. doi: 10.1371/journal.pgen.1000358 (PMC2627940; doi:10.1371/journal.pgen.1000358)
Supplement: Table S7 — Composition of Zap1 region in terms of SNPs and regulatory potentials. We list all SNPs in the Zap1 region. Each column contains the following information: SNP ID – the SNP ID (1-n); Gene – name of the gene where the SNP resides (including upstream and downstream regions); Loc – one of U, C and D representing Upstream, Coding region and Downstream, respectively; Regpot – learned regulatory potential of the SNP; Chr, Pos – chromosome, position of the SNP; BY-Nuc – nucleotide allele in BY, RM-Nuc – nucleotide allele in RM; BY-AA – corresponding AA in BY; and RM-AA – corresponding AA in RM. (0.4 MB DOC) [file pgen.1000358.s020.doc]

| **SNP ID** | **Gene** | **Loc** | **Regpot** | **Chr** | **Pos** | **BY-Nuc** | **RM-Nuc** | **BY-AA** | **RM-AA** |
| --- | --- | --- | --- | --- | --- | --- | --- | --- | --- |
| 1 | SCP160 | C | 0.563831 | 10 | 286103 | T | C | V | A |
| 2 | SCP160 | C | 0.634312 | 10 | 287654 | G | A | C | Y |
| 3 | SCP160 | C | 0.603427 | 10 | 288236 | T | C | L | S |
| 4 | SCP160 | C | 0.538612 | 10 | 288526 | C | T | A | A |
| 5 | PRY1 | U | 0.448097 | 10 | 290800 | C | T | _ | _ |
| 6 | PRY1 | U | 0.448097 | 10 | 290885 | A | G | _ | _ |
| 7 | PRY3 | D | 0.456516 | 10 | 290885 | A | G | _ | _ |
| 8 | PRY3 | D | 0.496403 | 10 | 291165 | C | A | _ | _ |
| 9 | PRY3 | C | 0.538813 | 10 | 291878 | C | T | T | I |
| 10 | PRY3 | C | 0.51808 | 10 | 291987 | G | A | D | N |
| 11 | PRY3 | C | 0.468472 | 10 | 292327 | C | - | S | - |
| 12 | PRY3 | C | 0.468472 | 10 | 292328 | C | - | S | - |
| 13 | PRY3 | C | 0.468472 | 10 | 292329 | T | - | S | - |
| 14 | PRY3 | C | 0.468472 | 10 | 292330 | A | - | Q | - |
| 15 | PRY3 | C | 0.468472 | 10 | 292331 | A | - | Q | - |
| 16 | PRY3 | C | 0.468472 | 10 | 292332 | C | - | Q | - |
| 17 | PRY3 | C | 0.468472 | 10 | 292333 | G | - | E | - |
| 18 | PRY3 | C | 0.468472 | 10 | 292334 | A | - | E | - |
| 19 | PRY3 | C | 0.468472 | 10 | 292335 | G | - | E | - |
| 20 | PRY3 | C | 0.476244 | 10 | 292354 | C | T | A | A |
| 21 | PRY3 | C | 0.556419 | 10 | 292533 | A | G | T | A |
| 22 | PRY3 | C | 0.491003 | 10 | 292847 | A | G | N | S |
| 23 | PRY3 | C | 0.507033 | 10 | 293026 | C | G | S | T |
| 24 | PRY3 | C | 0.507033 | 10 | 293027 | G | C | S | T |
| 25 | PRY3 | C | 0.528489 | 10 | 293029 | G | T | S | D |
| 26 | PRY3 | C | 0.528489 | 10 | 293030 | C | A | S | D |
| 27 | PRY3 | C | 0.528489 | 10 | 293031 | T | G | S | D |
| 28 | PRY3 | C | 0.476244 | 10 | 293032 | C | T | T | T |
| 29 | PRY3 | C | 0.53142 | 10 | 293035 | T | C | P | S |
| 30 | PRY3 | C | 0.53142 | 10 | 293036 | C | G | P | S |
| 31 | PRY3 | C | 0.53142 | 10 | 293037 | C | A | P | S |
| 32 | PRY3 | C | 0.565052 | 10 | 293038 | C | T | D | A |
| 33 | PRY3 | C | 0.565052 | 10 | 293039 | A | C | D | A |
| 34 | PRY3 | C | 0.507033 | 10 | 293041 | C | T | S | T |
| 35 | PRY3 | C | 0.507033 | 10 | 293043 | T | A | S | T |
| 36 | PRY3 | C | 0.561174 | 10 | 293045 | C | G | S | C |
| 37 | PRY3 | C | 0.545592 | 10 | 293047 | T | C | T | P |
| 38 | PRY3 | C | 0.545592 | 10 | 293049 | A | C | T | P |
| 39 | PRY3 | C | 0.507033 | 10 | 293052 | A | T | T | S |
| 40 | PRY3 | C | 0.507033 | 10 | 293054 | G | C | S | T |
| 41 | PRY3 | C | 0.491003 | 10 | 293105 | G | A | S | N |
| 42 | PRY3 | C | 0.53142 | 10 | 293139 | T | C | S | P |
| 43 | PRY3 | C | 0.476244 | 10 | 293448 | C | T | L | L |
| 44 | PRY3 | U | 0.478388 | 10 | 293997 | T | C | _ | _ |
| 45 | YJL077W-B | C | 0.556261 | 10 | 293997 | A | G | K | E |
| 46 | ICS3 | D | 0.456516 | 10 | 294249 | G | A | _ | _ |
| 47 | PRY3 | U | 0.448097 | 10 | 294249 | G | A | _ | _ |
| 48 | YJL077W-B | D | 0.456516 | 10 | 294249 | C | T | _ | _ |
| 49 | ICS3 | D | 0.456516 | 10 | 294250 | A | G | _ | _ |
| 50 | YJL077W-B | D | 0.456516 | 10 | 294250 | T | C | _ | _ |
| 51 | PRY3 | U | 0.448097 | 10 | 294250 | A | G | _ | _ |
| 52 | ICS3 | D | 0.456516 | 10 | 294398 | - | G | _ | _ |
| 53 | YJL077W-B | D | 0.456516 | 10 | 294406 | C | T | _ | _ |
| 54 | ICS3 | D | 0.456516 | 10 | 294407 | G | A | _ | _ |
| 55 | ICS3 | D | 0.496403 | 10 | 294505 | C | A | _ | _ |
| 56 | YJL077W-B | D | 0.456516 | 10 | 294505 | G | T | _ | _ |
| 57 | SMC3 | D | 0.496628 | 10 | 298832 | G | A | _ | _ |
| 58 | SMC3 | C | 0.521613 | 10 | 300543 | G | A | V | I |
| 59 | SMC3 | C | 0.60659 | 10 | 301784 | A | G | E | G |
| 60 | JEM1 | C | 0.608245 | 10 | 303185 | A | G | E | G |
| 61 | SMC3 | U | 0.488133 | 10 | 303186 | T | C | _ | _ |
| 62 | JEM1 | C | 0.586789 | 10 | 303233 | C | A | P | T |
| 63 | JEM1 | C | 0.582729 | 10 | 303500 | C | T | H | Y |
| 64 | ARG2 | U | 0.479624 | 10 | 305893 | G | T | _ | _ |
| 65 | PSF2 | U | 0.456911 | 10 | 305894 | C | A | _ | _ |
| 66 | ARG2 | C | 0.507929 | 10 | 306549 | G | A | K | K |
| 67 | ARG2 | C | 0.507929 | 10 | 307017 | C | T | G | G |
| 68 | ARG2 | C | 0.507929 | 10 | 307404 | C | G | G | G |
| 69 | YJL070C | D | 0.456516 | 10 | 307704 | A | G | _ | _ |
| 70 | ARG2 | C | 0.571962 | 10 | 307705 | T | C | L | L |
| 71 | YJL070C | C | 0.55531 | 10 | 309275 | A | G | N | S |
| 72 | UTP18 | D | 0.459272 | 10 | 310701 | A | G | _ | _ |
| 73 | YJL070C | U | 0.512441 | 10 | 310701 | A | G | _ | _ |
| 74 | UTP18 | C | 0.536402 | 10 | 311190 | A | C | Q | H |
| 75 | UTP18 | C | 0.559158 | 10 | 312320 | A | G | T | A |
| 76 | UTP18 | C | 0.491051 | 10 | 312399 | A | C | E | D |
| 77 | MPM1 | U | 0.448097 | 10 | 315154 | A | G | _ | _ |
| 78 | DLS1 | C | 0.527494 | 10 | 315154 | A | G | P | P |
| 79 | MRPL8 | C | 0.486058 | 10 | 316123 | G | A | L | L |
| 80 | MRPL8 | C | 0.511545 | 10 | 316201 | T | C | V | A |
| 81 | YJL062W-A | U | 0.448097 | 10 | 316202 | A | G | _ | _ |
| 82 | YJL062W-A | U | 0.448097 | 10 | 316451 | C | T | _ | _ |
| 83 | MRPL8 | U | 0.488204 | 10 | 316452 | G | A | _ | _ |
| 84 | MRPL8 | U | 0.488204 | 10 | 316487 | C | A | _ | _ |
| 85 | YJL062W-A | U | 0.448097 | 10 | 316488 | G | T | _ | _ |
| 86 | MRPL8 | U | 0.457837 | 10 | 316506 | A | G | _ | _ |
| 87 | YJL062W-A | U | 0.448097 | 10 | 316507 | T | C | _ | _ |
| 88 | YJL062W-A | D | 0.456516 | 10 | 317006 | T | - | _ | _ |
| 89 | YJL062W-A | D | 0.456516 | 10 | 317007 | A | - | _ | _ |
| 90 | YJL062W-A | D | 0.456516 | 10 | 317008 | T | - | _ | _ |
| 91 | YJL062W-A | D | 0.456516 | 10 | 317009 | A | - | _ | _ |
| 92 | LAS21 | U | 0.453905 | 10 | 317009 | A | - | _ | _ |
| 93 | LAS21 | U | 0.453905 | 10 | 317010 | T | - | _ | _ |
| 94 | YJL062W-A | D | 0.456516 | 10 | 317010 | T | - | _ | _ |
| 95 | LAS21 | U | 0.453905 | 10 | 317011 | A | - | _ | _ |
| 96 | YJL062W-A | D | 0.456516 | 10 | 317011 | A | - | _ | _ |
| 97 | LAS21 | U | 0.453905 | 10 | 317012 | T | - | _ | _ |
| 98 | LAS21 | U | 0.453905 | 10 | 317013 | A | - | _ | _ |
| 99 | LAS21 | U | 0.453905 | 10 | 317014 | T | - | _ | _ |
| 100 | LAS21 | C | 0.482099 | 10 | 319189 | C | T | S | S |
| 101 | NUP82 | U | 0.454899 | 10 | 319781 | C | T | _ | _ |
| 102 | LAS21 | D | 0.502268 | 10 | 319781 | C | T | _ | _ |
| 103 | NUP82 | C | 0.500865 | 10 | 320176 | C | T | L | F |
| 104 | NUP82 | C | 0.545631 | 10 | 320528 | C | T | T | I |
| 105 | NUP82 | C | 0.545631 | 10 | 320529 | C | T | T | I |
| 106 | BNA3 | U | 0.448097 | 10 | 322947 | G | A | _ | _ |
| 107 | BNA3 | U | 0.448097 | 10 | 322986 | A | G | _ | _ |
| 108 | BNA3 | U | 0.448097 | 10 | 323063 | T | C | _ | _ |
| 109 | BNA3 | U | 0.478388 | 10 | 323211 | G | A | _ | _ |
| 110 | BNA3 | U | 0.478388 | 10 | 323230 | G | A | _ | _ |
| 111 | BNA3 | U | 0.478388 | 10 | 323231 | G | A | _ | _ |
| 112 | BNA3 | U | 0.478388 | 10 | 323258 | C | T | _ | _ |
| 113 | BNA3 | U | 0.478388 | 10 | 323274 | C | T | _ | _ |
| 114 | BNA3 | U | 0.478388 | 10 | 323278 | T | C | _ | _ |
| 115 | BNA3 | U | 0.478388 | 10 | 323302 | A | G | _ | _ |
| 116 | BNA3 | C | 0.476244 | 10 | 323380 | C | T | F | F |
| 117 | BNA3 | C | 0.476244 | 10 | 323518 | C | G | A | A |
| 118 | BNA3 | C | 0.48847 | 10 | 323554 | G | A | M | I |
| 119 | BNA3 | C | 0.476244 | 10 | 323620 | T | C | S | S |
| 120 | BNA3 | C | 0.476244 | 10 | 323719 | C | T | G | G |
| 121 | BNA3 | C | 0.476244 | 10 | 323812 | T | C | P | P |
| 122 | BNA3 | C | 0.476244 | 10 | 324181 | T | C | Y | Y |
| 123 | BNA3 | C | 0.476244 | 10 | 324349 | A | C | L | L |
| 124 | BNA3 | C | 0.476244 | 10 | 324544 | T | C | A | A |
| 125 | YHC3 | U | 0.452965 | 10 | 324544 | T | C | _ | _ |
| 126 | BNA3 | D | 0.496403 | 10 | 324709 | G | A | _ | _ |
| 127 | YHC3 | U | 0.452965 | 10 | 324709 | G | A | _ | _ |
| 128 | YHC3 | U | 0.452965 | 10 | 324747 | T | A | _ | _ |
| 129 | BNA3 | D | 0.456516 | 10 | 324747 | T | A | _ | _ |
| 130 | YHC3 | C | 0.481152 | 10 | 325249 | G | A | L | L |
| 131 | YHC3 | C | 0.474183 | 10 | 325286 | A | C | I | L |
| 132 | YHC3 | C | 0.486325 | 10 | 325352 | G | A | V | I |
| 133 | YHC3 | C | 0.540873 | 10 | 325422 | A | T | Y | F |
| 134 | YHC3 | C | 0.481152 | 10 | 325675 | A | G | K | K |
| 135 | BIT61 | D | 0.473324 | 10 | 325720 | G | A | _ | _ |
| 136 | YHC3 | C | 0.481152 | 10 | 325720 | C | T | T | T |
| 137 | YHC3 | C | 0.493385 | 10 | 325776 | G | A | M | I |
| 138 | BIT61 | D | 0.473324 | 10 | 325777 | C | T | _ | _ |
| 139 | BIT61 | D | 0.473324 | 10 | 325862 | T | C | _ | _ |
| 140 | YHC3 | C | 0.52837 | 10 | 325863 | A | G | H | R |
| 141 | BIT61 | D | 0.473324 | 10 | 326007 | G | C | _ | _ |
| 142 | YHC3 | C | 0.481152 | 10 | 326008 | C | G | S | S |
| 143 | BIT61 | C | 0.49312 | 10 | 326209 | A | G | R | R |
| 144 | YHC3 | D | 0.4614 | 10 | 326210 | T | C | _ | _ |
| 145 | BIT61 | C | 0.49312 | 10 | 326338 | C | T | N | N |
| 146 | YHC3 | D | 0.4614 | 10 | 326339 | G | A | _ | _ |
| 147 | YHC3 | D | 0.4614 | 10 | 326413 | A | G | _ | _ |
| 148 | BIT61 | C | 0.49312 | 10 | 326414 | T | C | S | S |
| 149 | BIT61 | C | 0.49312 | 10 | 326533 | G | A | E | E |
| 150 | YHC3 | D | 0.4614 | 10 | 326534 | C | T | _ | _ |
| 151 | BIT61 | C | 0.49312 | 10 | 326600 | T | C | S | S |
| 152 | BIT61 | C | 0.534924 | 10 | 326632 | A | G | N | D |
| 153 | BIT61 | C | 0.49312 | 10 | 326906 | C | T | D | D |
| 154 | BIT61 | C | 0.498298 | 10 | 327418 | G | A | V | I |
| 155 | YJL057C | D | 0.520928 | 10 | 327635 | A | G | _ | _ |
| 156 | BIT61 | C | 0.49312 | 10 | 327635 | A | G | T | T |
| 157 | BIT61 | C | 0.523908 | 10 | 327726 | G | C | S | T |
| 158 | YJL057C | D | 0.520928 | 10 | 327726 | G | C | _ | _ |
| 159 | BIT61 | C | 0.537783 | 10 | 327768 | T | C | L | P |
| 160 | YJL057C | D | 0.520928 | 10 | 327768 | T | C | _ | _ |
| 161 | YJL057C | D | 0.520928 | 10 | 327795 | - | A | _ | _ |
| 162 | BIT61 | U | 0.495267 | 10 | 327795 | - | A | _ | _ |
| 163 | BIT61 | U | 0.495267 | 10 | 327863 | G | C | _ | _ |
| 164 | YJL057C | D | 0.520928 | 10 | 327863 | G | C | _ | _ |
| 165 | BIT61 | U | 0.46486 | 10 | 327903 | C | G | _ | _ |
| 166 | YJL057C | D | 0.520928 | 10 | 327903 | C | G | _ | _ |
| 167 | BIT61 | U | 0.46486 | 10 | 328079 | G | A | _ | _ |
| 168 | YJL057C | C | 0.545811 | 10 | 328079 | G | A | V | I |
| 169 | BIT61 | U | 0.46486 | 10 | 328191 | C | A | _ | _ |
| 170 | YJL057C | C | 0.540671 | 10 | 328191 | C | A | I | I |
| 171 | YJL057C | C | 0.540671 | 10 | 328368 | T | C | N | N |
| 172 | YJL057C | C | 0.540671 | 10 | 328656 | C | T | P | P |
| 173 | YJL057C | C | 0.552613 | 10 | 328794 | C | A | D | E |
| 174 | YJL057C | C | 0.540671 | 10 | 329007 | T | C | I | I |
| 175 | YJL057C | C | 0.540671 | 10 | 329109 | C | T | R | R |
| 176 | YJL057C | C | 0.545811 | 10 | 329240 | G | A | V | I |
| 177 | YJL057C | C | 0.532917 | 10 | 329241 | A | - | E | - |
| 178 | YJL057C | C | 0.532917 | 10 | 329242 | A | - | E | - |
| 179 | YJL057C | C | 0.532917 | 10 | 329243 | G | - | E | - |
| 180 | YJL057C | C | 0.605408 | 10 | 329647 | C | T | S | L |
| 181 | YJL057C | C | 0.540671 | 10 | 329775 | A | G | R | R |
| 182 | YJL057C | C | 0.540671 | 10 | 329820 | G | A | V | V |
| 183 | ZAP1 | D | 0.989606 | 10 | 329918 | A | G | _ | _ |
| 184 | YJL057C | C | 0.581879 | 10 | 329918 | A | G | N | D |
| 185 | ZAP1 | D | 0.991952 | 10 | 329967 | T | C | _ | _ |
| 186 | YJL057C | C | 0.476244 | 10 | 329967 | T | C | V | V |
| 187 | ZAP1 | D | 0.989606 | 10 | 330061 | G | A | _ | _ |
| 188 | YJL057C | U | 0.542804 | 10 | 330061 | G | A | _ | _ |
| 189 | YJL057C | U | 0.542804 | 10 | 330085 | C | A | _ | _ |
| 190 | ZAP1 | D | 0.989606 | 10 | 330085 | C | A | _ | _ |
| 191 | ZAP1 | D | 0.989606 | 10 | 330108 | T | C | _ | _ |
| 192 | YJL057C | U | 0.542804 | 10 | 330108 | T | C | _ | _ |
| 193 | ZAP1 | D | 0.989606 | 10 | 330137 | - | G | _ | _ |
| 194 | YJL057C | U | 0.542804 | 10 | 330137 | - | G | _ | _ |
| 195 | YJL057C | U | 0.512441 | 10 | 330289 | A | T | _ | _ |
| 196 | ZAP1 | D | 0.991129 | 10 | 330289 | A | T | _ | _ |
| 197 | ZAP1 | C | 0.992349 | 10 | 330387 | G | C | Q | H |
| 198 | YJL057C | U | 0.512441 | 10 | 330387 | G | C | _ | _ |
| 199 | YJL057C | U | 0.512441 | 10 | 330474 | G | A | _ | _ |
| 200 | ZAP1 | C | 0.990391 | 10 | 330474 | G | A | L | L |
| 201 | ZAP1 | C | 0.990391 | 10 | 330684 | G | A | K | K |
| 202 | ZAP1 | C | 0.992976 | 10 | 330947 | G | C | D | H |
| 203 | ZAP1 | C | 0.990391 | 10 | 330948 | A | T | A | A |
| 204 | ZAP1 | C | 0.993434 | 10 | 330961 | A | C | E | A |
| 205 | ZAP1 | C | 0.992505 | 10 | 330967 | C | T | T | I |
| 206 | ZAP1 | C | 0.990391 | 10 | 330981 | C | T | C | C |
| 207 | ZAP1 | C | 0.991044 | 10 | 331026 | T | G | F | L |
| 208 | ZAP1 | C | 0.990391 | 10 | 331329 | C | T | S | S |
| 209 | ZAP1 | C | 0.991277 | 10 | 331556 | G | A | V | M |
| 210 | ZAP1 | C | 0.990391 | 10 | 331728 | T | C | N | N |
| 211 | ZAP1 | C | 0.992349 | 10 | 331782 | C | G | H | Q |
| 212 | ZAP1 | C | 0.990391 | 10 | 331851 | G | A | L | L |
| 213 | ZAP1 | C | 0.992691 | 10 | 332458 | C | T | S | F |
| 214 | ZAP1 | C | 0.994424 | 10 | 332512 | T | G | L | R |
| 215 | ZAP1 | C | 0.990391 | 10 | 332514 | C | T | D | D |
| 216 | ZAP1 | C | 0.990391 | 10 | 332538 | T | C | S | S |
| 217 | ZAP1 | C | 0.991495 | 10 | 332582 | T | A | S | T |
| 218 | YJL055W | U | 0.452178 | 10 | 332991 | A | G | _ | _ |
| 219 | ZAP1 | U | 0.990472 | 10 | 332992 | T | C | _ | _ |
| 220 | ZAP1 | U | 0.990472 | 10 | 333052 | G | A | _ | _ |
| 221 | YJL055W | U | 0.452178 | 10 | 333053 | C | T | _ | _ |
| 222 | ZAP1 | U | 0.989251 | 10 | 333328 | T | C | _ | _ |
| 223 | YJL055W | C | 0.520626 | 10 | 333329 | A | G | K | R |
| 224 | YJL055W | D | 0.500526 | 10 | 334042 | C | T | _ | _ |
| 225 | TIM54 | U | 0.463739 | 10 | 334042 | C | T | _ | _ |
| 226 | TIM54 | U | 0.494141 | 10 | 334121 | A | G | _ | _ |
| 227 | YJL055W | D | 0.46061 | 10 | 334121 | A | G | _ | _ |
| 228 | TIM54 | U | 0.494141 | 10 | 334181 | C | T | _ | _ |
| 229 | YJL055W | D | 0.46061 | 10 | 334181 | C | T | _ | _ |
| 230 | YJL055W | D | 0.46061 | 10 | 334208 | T | C | _ | _ |
| 231 | TIM54 | C | 0.491993 | 10 | 334208 | T | C | I | I |
| 232 | TIM54 | C | 0.491993 | 10 | 334598 | A | G | R | R |
| 233 | TIM54 | C | 0.491993 | 10 | 334775 | C | T | I | I |
| 234 | TIM54 | C | 0.54551 | 10 | 335065 | C | T | T | M |
| 235 | TIM54 | D | 0.512166 | 10 | 335705 | C | A | _ | _ |
| 236 | PEP8 | U | 0.461589 | 10 | 335705 | C | A | _ | _ |
| 237 | PEP8 | C | 0.489832 | 10 | 336300 | G | A | K | K |
| 238 | PEP8 | C | 0.489832 | 10 | 336429 | C | T | I | I |
| 239 | PEP8 | C | 0.489832 | 10 | 336468 | C | T | N | N |
| 240 | PEP8 | C | 0.505442 | 10 | 336500 | C | G | A | G |
| 241 | PEP8 | C | 0.53702 | 10 | 336530 | A | G | H | R |
| 242 | PEP8 | C | 0.489832 | 10 | 336642 | G | A | G | G |
| 243 | PEP8 | C | 0.489832 | 10 | 336672 | G | A | R | R |
| 244 | PEP8 | C | 0.489832 | 10 | 336861 | T | C | F | F |
| 245 | PEP8 | D | 0.510006 | 10 | 336967 | T | A | _ | _ |
| 246 | PEP8 | D | 0.510006 | 10 | 337004 | C | T | _ | _ |
| 247 | PEP8 | D | 0.510006 | 10 | 337050 | A | G | _ | _ |
| 248 | PEP8 | D | 0.470046 | 10 | 337139 | A | C | _ | _ |
| 249 | PEP8 | D | 0.470046 | 10 | 337180 | A | C | _ | _ |
| 250 | PEP8 | D | 0.470046 | 10 | 337262 | A | C | _ | _ |
| 251 | PEP8 | D | 0.470046 | 10 | 337316 | A | - | _ | _ |
| 252 | YJL052C-A | D | 0.456516 | 10 | 337352 | T | A | _ | _ |
| 253 | PEP8 | D | 0.470046 | 10 | 337353 | A | T | _ | _ |
| 254 | YJL052C-A | D | 0.456516 | 10 | 337385 | C | T | _ | _ |
| 255 | PEP8 | D | 0.470046 | 10 | 337385 | G | A | _ | _ |
| 256 | YJL052C-A | D | 0.456516 | 10 | 337468 | A | G | _ | _ |
| 257 | YJL052C-A | D | 0.456516 | 10 | 337496 | G | A | _ | _ |
| 258 | YJL052C-A | D | 0.456516 | 10 | 337644 | T | A | _ | _ |
| 259 | YJL052C-A | C | 0.604396 | 10 | 337811 | C | T | R | C |
| 260 | TDH1 | U | 0.502193 | 10 | 337812 | G | A | _ | _ |
| 261 | YJL052C-A | U | 0.478388 | 10 | 337960 | C | - | _ | _ |
| 262 | TDH1 | U | 0.502193 | 10 | 337966 | G | - | _ | _ |
| 263 | TDH1 | C | 0.530473 | 10 | 338910 | T | C | V | V |
| 264 | TDH1 | C | 0.623862 | 10 | 338930 | A | C | E | A |
| 265 | TDH1 | C | 0.530473 | 10 | 338967 | T | C | V | V |
| 266 | YJL051W | U | 0.512441 | 10 | 339263 | G | A | _ | _ |
| 267 | TDH1 | D | 0.550515 | 10 | 339263 | G | A | _ | _ |
| 268 | TDH1 | D | 0.510689 | 10 | 339343 | C | T | _ | _ |
| 269 | YJL051W | U | 0.512441 | 10 | 339343 | C | T | _ | _ |
| 270 | TDH1 | D | 0.510689 | 10 | 339346 | T | C | _ | _ |
| 271 | YJL051W | U | 0.512441 | 10 | 339346 | T | C | _ | _ |
| 272 | YJL051W | U | 0.512441 | 10 | 339363 | T | A | _ | _ |
| 273 | TDH1 | D | 0.510689 | 10 | 339363 | T | A | _ | _ |
| 274 | TDH1 | D | 0.510689 | 10 | 339368 | G | A | _ | _ |
| 275 | YJL051W | U | 0.512441 | 10 | 339368 | G | A | _ | _ |
| 276 | YJL051W | U | 0.512441 | 10 | 339410 | C | T | _ | _ |
| 277 | TDH1 | D | 0.510689 | 10 | 339410 | C | T | _ | _ |
| 278 | TDH1 | D | 0.510689 | 10 | 339429 | A | T | _ | _ |
| 279 | YJL051W | U | 0.512441 | 10 | 339429 | A | T | _ | _ |
| 280 | YJL051W | U | 0.512441 | 10 | 339574 | C | T | _ | _ |
| 281 | TDH1 | D | 0.510689 | 10 | 339574 | C | T | _ | _ |
| 282 | YJL051W | U | 0.512441 | 10 | 339586 | C | G | _ | _ |
| 283 | TDH1 | D | 0.510689 | 10 | 339586 | C | G | _ | _ |
| 284 | YJL051W | U | 0.542804 | 10 | 339677 | A | - | _ | _ |
| 285 | YJL051W | U | 0.542804 | 10 | 339678 | T | - | _ | _ |
| 286 | YJL051W | C | 0.558266 | 10 | 339913 | T | G | F | L |
| 287 | YJL051W | C | 0.540671 | 10 | 339931 | T | C | H | H |
| 288 | YJL051W | C | 0.540671 | 10 | 340012 | C | T | T | T |
| 289 | YJL051W | C | 0.540671 | 10 | 340076 | T | C | L | L |
| 290 | YJL051W | C | 0.581848 | 10 | 340382 | T | G | S | A |
| 291 | YJL051W | C | 0.540671 | 10 | 340654 | G | A | G | G |
| 292 | YJL051W | C | 0.594834 | 10 | 340679 | C | T | P | S |
| 293 | YJL051W | C | 0.540671 | 10 | 340864 | G | A | Q | Q |
| 294 | YJL051W | C | 0.540671 | 10 | 341207 | C | T | L | L |
| 295 | YJL051W | C | 0.618876 | 10 | 341213 | G | A | A | T |
| 296 | YJL051W | C | 0.573329 | 10 | 341339 | C | G | P | A |
| 297 | YJL051W | C | 0.564805 | 10 | 341483 | A | G | M | V |
| 298 | YJL051W | C | 0.540671 | 10 | 341503 | T | C | S | S |
| 299 | YJL051W | C | 0.577774 | 10 | 341714 | G | A | G | S |
| 300 | YJL051W | C | 0.55531 | 10 | 341760 | A | G | N | S |
| 301 | YJL051W | C | 0.604511 | 10 | 341930 | C | T | H | Y |
| 302 | MTR4 | U | 0.465325 | 10 | 342207 | T | C | _ | _ |
| 303 | YJL051W | D | 0.560638 | 10 | 342207 | T | C | _ | _ |
| 304 | YJL051W | D | 0.560638 | 10 | 342234 | T | - | _ | _ |
| 305 | MTR4 | U | 0.465325 | 10 | 342234 | T | - | _ | _ |
| 306 | MTR4 | U | 0.465325 | 10 | 342272 | T | G | _ | _ |
| 307 | YJL051W | D | 0.520928 | 10 | 342272 | T | G | _ | _ |
| 308 | YJL051W | D | 0.520928 | 10 | 342282 | T | - | _ | _ |
| 309 | YJL051W | D | 0.520928 | 10 | 342283 | T | - | _ | _ |
| 310 | MTR4 | U | 0.465325 | 10 | 342284 | T | - | _ | _ |
| 311 | YJL051W | D | 0.520928 | 10 | 342284 | T | - | _ | _ |
| 312 | MTR4 | U | 0.465325 | 10 | 342285 | T | - | _ | _ |
| 313 | YJL051W | D | 0.520928 | 10 | 342285 | T | - | _ | _ |
| 314 | YJL051W | D | 0.520928 | 10 | 342286 | T | - | _ | _ |
| 315 | MTR4 | U | 0.465325 | 10 | 342286 | T | - | _ | _ |
| 316 | MTR4 | U | 0.465325 | 10 | 342287 | T | - | _ | _ |
| 317 | MTR4 | U | 0.465325 | 10 | 342288 | T | - | _ | _ |
| 318 | MTR4 | U | 0.495734 | 10 | 342374 | C | G | _ | _ |
| 319 | YJL051W | D | 0.520928 | 10 | 342374 | C | G | _ | _ |
| 320 | MTR4 | U | 0.495734 | 10 | 342396 | T | C | _ | _ |
| 321 | YJL051W | D | 0.520928 | 10 | 342396 | T | C | _ | _ |
| 322 | MTR4 | U | 0.495734 | 10 | 342422 | T | C | _ | _ |
| 323 | YJL051W | D | 0.520928 | 10 | 342422 | T | C | _ | _ |
| 324 | YJL051W | D | 0.456516 | 10 | 342475 | A | G | _ | _ |
| 325 | MTR4 | C | 0.635114 | 10 | 342475 | A | G | T | A |
| 326 | MTR4 | C | 0.493587 | 10 | 342768 | C | T | D | D |
| 327 | MTR4 | C | 0.493587 | 10 | 342873 | A | T | T | T |
| 328 | MTR4 | C | 0.493587 | 10 | 343329 | A | G | P | P |
| 329 | MTR4 | C | 0.493587 | 10 | 343362 | A | G | K | K |
| 330 | MTR4 | C | 0.493587 | 10 | 343740 | T | C | D | D |
| 331 | MTR4 | C | 0.493587 | 10 | 343773 | C | T | N | N |
| 332 | MTR4 | C | 0.493587 | 10 | 343788 | A | G | L | L |
| 333 | MTR4 | C | 0.493587 | 10 | 343837 | T | C | L | L |
| 334 | MTR4 | C | 0.511356 | 10 | 344311 | C | T | L | F |
| 335 | MTR4 | C | 0.558628 | 10 | 344377 | C | T | H | Y |
| 336 | MTR4 | C | 0.493587 | 10 | 344649 | T | C | P | P |
| 337 | MTR4 | C | 0.511356 | 10 | 344664 | A | C | L | F |
| 338 | MTR4 | C | 0.505634 | 10 | 344979 | A | T | E | D |
| 339 | MTR4 | C | 0.493587 | 10 | 345063 | C | G | A | A |
| 340 | MTR4 | C | 0.493587 | 10 | 345282 | C | T | R | R |
| 341 | YJL049W | U | 0.448097 | 10 | 345704 | A | - | _ | _ |
| 342 | MTR4 | D | 0.513759 | 10 | 345704 | A | - | _ | _ |
| 343 | YJL049W | U | 0.448097 | 10 | 345748 | T | A | _ | _ |
| 344 | MTR4 | D | 0.513759 | 10 | 345748 | T | A | _ | _ |
