# Supplementary material for: Learning a Prior on Regulatory Potential from eQTL Data
Source: PLoS Genet. 2009 Jan 30;5(1):e1000358. doi: 10.1371/journal.pgen.1000358 (PMC2627940; doi:10.1371/journal.pgen.1000358)
Supplement: Table S8 — Composition of Oaf1 region in terms of SNPs and regulatory potentials. We list all SNPs in the Oaf1 region. Each column contains the following information: SNP ID – the SNP ID (1-n); Gene – name of the gene where the SNP resides (including upstream and downstream regions); Loc – one of U, C and D representing Upstream, Coding region and Downstream, respectively; Regpot – learned regulatory potential of the SNP; Chr, Pos – chromosome, position of the SNP; BY-Nuc – nucleotide allele in BY, RM-Nuc – nucleotide allele in RM; BY-AA – corresponding AA in BY; and RM-AA – corresponding AA in RM. (0.2 MB DOC) [file pgen.1000358.s021.doc]

| **SNP ID** | **Gene** | **Loc** | **Regpot** | **Chr** | **Pos** | **BY-Nuc** | **RM-Nuc** | **BY-AA** | **RM-AA** |
| --- | --- | --- | --- | --- | --- | --- | --- | --- | --- |
| 1 | GPB2 | C | 0.631 | 1 | 39796 | G | A | Q | Q |
| 2 | GPB2 | C | 0.689 | 1 | 39902 | T | C | Y | H |
| 3 | GPB2 | C | 0.672 | 1 | 39920 | T | C | L | P |
| 4 | GPB2 | C | 0.672 | 1 | 39921 | T | C | L | P |
| 5 | GPB2 | C | 0.631 | 1 | 39964 | G | A | K | K |
| 6 | GPB2 | C | 0.672 | 1 | 40065 | C | T | P | L |
| 7 | GPB2 | C | 0.631 | 1 | 40231 | C | G | P | P |
| 8 | GPB2 | C | 0.631 | 1 | 40238 | C | T | L | L |
| 9 | GPB2 | C | 0.631 | 1 | 40381 | G | A | T | T |
| 10 | GPB2 | C | 0.631 | 1 | 40861 | A | G | K | K |
| 11 | GPB2 | C | 0.631 | 1 | 40867 | T | C | G | G |
| 12 | GPB2 | C | 0.726 | 1 | 40915 | C | G | S | R |
| 13 | GPB2 | C | 0.636 | 1 | 41240 | A | G | I | V |
| 14 | GPB2 | C | 0.702 | 1 | 41279 | G | A | A | T |
| 15 | GPB2 | C | 0.631 | 1 | 41482 | A | G | V | V |
| 16 | GPB2 | C | 0.631 | 1 | 41500 | A | G | R | R |
| 17 | GPB2 | C | 0.681 | 1 | 41588 | C | T | P | S |
| 18 | GPB2 | C | 0.706 | 1 | 41664 | T | G | F | C |
| 19 | GPB2 | C | 0.715 | 1 | 41700 | C | A | A | E |
| 20 | GPB2 | C | 0.71 | 1 | 41703 | C | A | A | D |
| 21 | GPB2 | C | 0.631 | 1 | 41737 | T | C | D | D |
| 22 | PEX22 | U | 0.506 | 1 | 41737 | T | C | _ | _ |
| 23 | GPB2 | C | 0.569 | 1 | 41740 | A | G | T | T |
| 24 | PEX22 | U | 0.57 | 1 | 41740 | A | G | _ | _ |
| 25 | GPB2 | C | 0.631 | 1 | 41803 | G | A | L | L |
| 26 | PEX22 | U | 0.506 | 1 | 41803 | G | A | _ | _ |
| 27 | PEX22 | U | 0.57 | 1 | 41884 | C | T | _ | _ |
| 28 | GPB2 | C | 0.569 | 1 | 41884 | C | T | S | S |
| 29 | PEX22 | U | 0.506 | 1 | 41907 | G | A | _ | _ |
| 30 | GPB2 | D | 0.65 | 1 | 41907 | G | A | _ | _ |
| 31 | PEX22 | U | 0.506 | 1 | 41988 | C | T | _ | _ |
| 32 | GPB2 | D | 0.65 | 1 | 41988 | C | T | _ | _ |
| 33 | GPB2 | D | 0.612 | 1 | 42116 | T | C | _ | _ |
| 34 | PEX22 | U | 0.537 | 1 | 42116 | T | C | _ | _ |
| 35 | GPB2 | D | 0.55 | 1 | 42258 | G | A | _ | _ |
| 36 | PEX22 | C | 0.598 | 1 | 42258 | G | A | T | T |
| 37 | ACS1 | D | 0.52 | 1 | 42484 | C | T | _ | _ |
| 38 | PEX22 | C | 0.682 | 1 | 42485 | G | A | G | E |
| 39 | ACS1 | D | 0.583 | 1 | 42527 | A | G | _ | _ |
| 40 | PEX22 | C | 0.534 | 1 | 42528 | T | C | Y | Y |
| 41 | PEX22 | C | 0.598 | 1 | 42554 | A | T | S | S |
| 42 | ACS1 | D | 0.52 | 1 | 42555 | T | A | _ | _ |
| 43 | ACS1 | D | 0.583 | 1 | 42562 | C | T | _ | _ |
| 44 | PEX22 | C | 0.574 | 1 | 42563 | G | A | R | K |
| 45 | ACS1 | D | 0.583 | 1 | 42590 | C | T | _ | _ |
| 46 | PEX22 | C | 0.534 | 1 | 42591 | G | A | L | L |
| 47 | PEX22 | C | 0.534 | 1 | 42629 | A | G | A | A |
| 48 | ACS1 | D | 0.583 | 1 | 42630 | T | C | _ | _ |
| 49 | ACS1 | D | 0.52 | 1 | 42635 | G | A | _ | _ |
| 50 | PEX22 | C | 0.598 | 1 | 42636 | C | T | V | V |
| 51 | ACS1 | D | 0.583 | 1 | 42684 | C | T | _ | _ |
| 52 | PEX22 | C | 0.54 | 1 | 42685 | G | A | V | I |
| 53 | ACS1 | C | 0.603 | 1 | 43004 | C | T | S | S |
| 54 | PEX22 | D | 0.515 | 1 | 43005 | G | A | _ | _ |
| 55 | PEX22 | D | 0.515 | 1 | 43137 | C | T | _ | _ |
| 56 | ACS1 | C | 0.617 | 1 | 43138 | G | A | S | N |
| 57 | ACS1 | C | 0.603 | 1 | 43479 | C | T | N | N |
| 58 | ACS1 | C | 0.603 | 1 | 43566 | T | C | V | V |
| 59 | ACS1 | C | 0.603 | 1 | 43692 | G | A | E | E |
| 60 | ACS1 | C | 0.603 | 1 | 43785 | G | C | A | A |
| 61 | ACS1 | C | 0.603 | 1 | 44385 | T | C | G | G |
| 62 | ACS1 | C | 0.603 | 1 | 44589 | T | C | T | T |
| 63 | ACS1 | C | 0.603 | 1 | 44676 | C | T | G | G |
| 64 | ACS1 | C | 0.603 | 1 | 44892 | T | C | T | T |
| 65 | ACS1 | C | 0.642 | 1 | 44936 | G | T | A | S |
| 66 | ACS1 | U | 0.605 | 1 | 45025 | C | T | _ | _ |
| 67 | ACS1 | U | 0.605 | 1 | 45031 | - | C | _ | _ |
| 68 | ACS1 | U | 0.575 | 1 | 45199 | C | T | _ | _ |
| 69 | ACS1 | U | 0.575 | 1 | 45360 | - | C | _ | _ |
| 70 | ACS1 | U | 0.575 | 1 | 45360 | - | C | _ | _ |
| 71 | ACS1 | U | 0.575 | 1 | 45360 | - | C | _ | _ |
| 72 | ACS1 | U | 0.575 | 1 | 45432 | C | T | _ | _ |
| 73 | YAL053W | U | 0.49 | 1 | 45526 | T | - | _ | _ |
| 74 | YAL053W | U | 0.49 | 1 | 45527 | G | - | _ | _ |
| 75 | YAL053W | U | 0.49 | 1 | 45547 | A | T | _ | _ |
| 76 | YAL053W | U | 0.49 | 1 | 45652 | - | T | _ | _ |
| 77 | YAL053W | U | 0.521 | 1 | 45832 | G | C | _ | _ |
| 78 | YAL053W | C | 0.518 | 1 | 46031 | G | A | L | L |
| 79 | YAL053W | C | 0.602 | 1 | 46231 | C | G | S | C |
| 80 | YAL053W | C | 0.53 | 1 | 46376 | A | T | E | D |
| 81 | YAL053W | C | 0.518 | 1 | 46724 | A | G | S | S |
| 82 | YAL053W | C | 0.56 | 1 | 46833 | G | A | D | N |
| 83 | YAL053W | C | 0.518 | 1 | 46838 | G | A | L | L |
| 84 | YAL053W | C | 0.518 | 1 | 46847 | T | C | T | T |
| 85 | YAL053W | C | 0.518 | 1 | 46895 | A | G | R | R |
| 86 | YAL053W | C | 0.518 | 1 | 46913 | T | G | A | A |
| 87 | YAL053W | C | 0.518 | 1 | 46967 | T | C | F | F |
| 88 | YAL053W | C | 0.518 | 1 | 47045 | A | C | I | I |
| 89 | YAL053W | C | 0.518 | 1 | 47207 | A | G | V | V |
| 90 | YAL053W | C | 0.518 | 1 | 47519 | A | G | P | P |
| 91 | YAL053W | C | 0.518 | 1 | 47661 | T | C | L | L |
| 92 | YAL053W | C | 0.518 | 1 | 47691 | T | C | L | L |
| 93 | YAL053W | C | 0.578 | 1 | 47821 | T | A | I | N |
| 94 | YAL053W | C | 0.573 | 1 | 47826 | C | T | P | S |
| 95 | YAL053W | C | 0.518 | 1 | 47897 | C | T | D | D |
| 96 | OAF1 | U | 0.616 | 1 | 48116 | G | C | _ | _ |
| 97 | YAL053W | C | 0.518 | 1 | 48116 | G | C | A | A |
| 98 | OAF1 | U | 0.616 | 1 | 48287 | A | T | _ | _ |
| 99 | YAL053W | D | 0.539 | 1 | 48287 | A | T | _ | _ |
| 100 | OAF1 | C | 0.701 | 1 | 48752 | T | C | L | S |
| 101 | OAF1 | C | 0.755 | 1 | 48772 | T | A | W | R |
| 102 | OAF1 | C | 0.643 | 1 | 48816 | T | C | G | G |
| 103 | OAF1 | C | 0.643 | 1 | 48891 | C | T | D | D |
| 104 | OAF1 | C | 0.643 | 1 | 49104 | C | T | T | T |
| 105 | OAF1 | C | 0.698 | 1 | 49115 | T | C | I | T |
| 106 | OAF1 | C | 0.643 | 1 | 49248 | C | T | F | F |
| 107 | OAF1 | C | 0.643 | 1 | 49404 | C | T | F | F |
| 108 | OAF1 | C | 0.643 | 1 | 49590 | T | C | I | I |
| 109 | OAF1 | C | 0.643 | 1 | 49872 | C | T | Y | Y |
| 110 | OAF1 | C | 0.699 | 1 | 49904 | C | A | P | Q |
| 111 | OAF1 | C | 0.643 | 1 | 50025 | C | T | H | H |
| 112 | OAF1 | C | 0.643 | 1 | 50205 | A | G | K | K |
| 113 | OAF1 | C | 0.709 | 1 | 50327 | C | A | T | K |
| 114 | OAF1 | C | 0.713 | 1 | 50344 | A | G | K | E |
| 115 | OAF1 | C | 0.643 | 1 | 50355 | C | T | F | F |
| 116 | OAF1 | C | 0.643 | 1 | 50385 | G | A | S | S |
| 117 | OAF1 | C | 0.643 | 1 | 50433 | T | C | H | H |
| 118 | OAF1 | C | 0.643 | 1 | 50457 | G | A | K | K |
| 119 | OAF1 | C | 0.643 | 1 | 50499 | A | G | L | L |
| 120 | OAF1 | C | 0.643 | 1 | 50661 | T | A | L | L |
| 121 | OAF1 | C | 0.643 | 1 | 50664 | C | T | I | I |
| 122 | OAF1 | C | 0.643 | 1 | 50841 | A | G | T | T |
| 123 | OAF1 | C | 0.643 | 1 | 51096 | G | A | R | R |
| 124 | OAF1 | C | 0.643 | 1 | 51288 | T | C | R | R |
| 125 | OAF1 | C | 0.643 | 1 | 51322 | T | C | L | L |
| 126 | OAF1 | C | 0.643 | 1 | 51372 | A | C | G | G |
| 127 | OAF1 | C | 0.643 | 1 | 51434 | A | G | S | S |
| 128 | YAL049C | D | 0.517 | 1 | 51435 | T | C | _ | _ |
| 129 | OAF1 | D | 0.624 | 1 | 51951 | T | G | _ | _ |
| 130 | YAL049C | C | 0.537 | 1 | 51952 | A | C | A | A |
| 131 | OAF1 | D | 0.624 | 1 | 52125 | C | T | _ | _ |
| 132 | YAL049C | C | 0.542 | 1 | 52125 | G | A | V | I |
| 133 | YAL049C | C | 0.537 | 1 | 52165 | G | A | G | G |
| 134 | YAL049C | C | 0.554 | 1 | 52311 | T | C | F | L |
| 135 | GEM1 | D | 0.458 | 1 | 52604 | T | A | _ | _ |
| 136 | YAL049C | U | 0.539 | 1 | 52604 | T | A | _ | _ |
| 137 | YAL049C | U | 0.539 | 1 | 52614 | C | T | _ | _ |
| 138 | GEM1 | D | 0.458 | 1 | 52614 | C | T | _ | _ |
| 139 | GEM1 | D | 0.458 | 1 | 52633 | A | G | _ | _ |
| 140 | YAL049C | U | 0.539 | 1 | 52633 | A | G | _ | _ |
| 141 | GEM1 | D | 0.497 | 1 | 52734 | T | C | _ | _ |
| 142 | YAL049C | U | 0.509 | 1 | 52734 | T | C | _ | _ |
| 143 | YAL049C | U | 0.509 | 1 | 52847 | C | T | _ | _ |
| 144 | GEM1 | C | 0.477 | 1 | 52847 | C | T | S | S |
| 145 | GEM1 | C | 0.477 | 1 | 52850 | T | C | C | C |
| 146 | YAL049C | U | 0.509 | 1 | 52850 | T | C | _ | _ |
| 147 | GEM1 | C | 0.477 | 1 | 52859 | A | G | V | V |
| 148 | YAL049C | U | 0.509 | 1 | 52859 | A | G | _ | _ |
| 149 | GEM1 | C | 0.542 | 1 | 52940 | G | A | P | P |
| 150 | YAL049C | U | 0.444 | 1 | 52940 | G | A | _ | _ |
| 151 | GEM1 | C | 0.477 | 1 | 52952 | C | A | G | G |
| 152 | YAL049C | U | 0.509 | 1 | 52952 | C | A | _ | _ |
| 153 | GEM1 | C | 0.542 | 1 | 52985 | T | C | F | F |
| 154 | YAL049C | U | 0.444 | 1 | 52985 | T | C | _ | _ |
| 155 | GEM1 | C | 0.542 | 1 | 52997 | T | C | L | L |
| 156 | YAL049C | U | 0.444 | 1 | 52997 | T | C | _ | _ |
| 157 | GEM1 | C | 0.477 | 1 | 53147 | A | C | T | T |
| 158 | GEM1 | C | 0.477 | 1 | 53168 | T | C | V | V |
| 159 | GEM1 | C | 0.477 | 1 | 53267 | G | A | Q | Q |
| 160 | GEM1 | C | 0.477 | 1 | 53543 | G | A | Q | Q |
| 161 | GEM1 | C | 0.477 | 1 | 53837 | T | C | V | V |
| 162 | GEM1 | C | 0.518 | 1 | 53844 | G | A | R | K |
| 163 | GEM1 | C | 0.477 | 1 | 53852 | C | T | L | L |
| 164 | GEM1 | C | 0.477 | 1 | 53936 | T | A | A | A |
| 165 | GEM1 | C | 0.477 | 1 | 54176 | T | C | A | A |
| 166 | GEM1 | C | 0.477 | 1 | 54199 | C | T | L | L |
| 167 | GEM1 | C | 0.477 | 1 | 54245 | T | C | A | A |
| 168 | GEM1 | C | 0.477 | 1 | 54320 | A | G | E | E |
| 169 | GEM1 | C | 0.477 | 1 | 54479 | C | T | S | S |
| 170 | GEM1 | C | 0.477 | 1 | 54482 | A | G | R | R |
| 171 | GEM1 | C | 0.477 | 1 | 54509 | T | C | V | V |
| 172 | SPC72 | D | 0.504 | 1 | 54662 | C | A | _ | _ |
| 173 | GEM1 | C | 0.477 | 1 | 54662 | C | A | I | I |
| 174 | GEM1 | C | 0.477 | 1 | 54680 | G | A | V | V |
| 175 | SPC72 | D | 0.504 | 1 | 54680 | G | A | _ | _ |
| 176 | GEM1 | C | 0.477 | 1 | 54743 | T | G | V | V |
| 177 | SPC72 | D | 0.504 | 1 | 54743 | T | G | _ | _ |
| 178 | SPC72 | D | 0.504 | 1 | 54785 | T | C | _ | _ |
| 179 | GEM1 | C | 0.477 | 1 | 54785 | T | C | T | T |
| 180 | SPC72 | D | 0.504 | 1 | 54807 | A | G | _ | _ |
| 181 | GEM1 | U | 0.479 | 1 | 54807 | A | G | _ | _ |
| 182 | SPC72 | C | 0.523 | 1 | 55146 | G | A | L | L |
| 183 | GEM1 | U | 0.449 | 1 | 55146 | G | A | _ | _ |
| 184 | GEM1 | U | 0.449 | 1 | 55172 | T | C | _ | _ |
| 185 | SPC72 | C | 0.523 | 1 | 55172 | T | C | L | L |
| 186 | SPC72 | C | 0.565 | 1 | 55214 | T | G | S | A |
| 187 | GEM1 | U | 0.449 | 1 | 55214 | T | G | _ | _ |
