# Supplementary material for: Learning a Prior on Regulatory Potential from eQTL Data
Source: PLoS Genet. 2009 Jan 30;5(1):e1000358. doi: 10.1371/journal.pgen.1000358 (PMC2627940; doi:10.1371/journal.pgen.1000358)
Supplement: Table S9 — Composition of Mkt1 region in terms of SNPs and regulatory potentials. We list all SNPs in the Mkt1 region. Each column contains the following information: SNP ID – the SNP ID (1-n); Gene – name of the gene where the SNP resides (including upstream and downstream regions); Loc – one of U, C and D representing Upstream, Coding region and Downstream, respectively; Regpot – learned regulatory potential of the SNP; Chr, Pos – chromosome, position of the SNP; BY-Nuc – nucleotide allele in BY, RM-Nuc – nucleotide allele in RM; BY-AA – corresponding AA in BY; and RM-AA – corresponding AA in RM. (0.2 MB DOC) [file pgen.1000358.s022.doc]

| **SNP ID** | **Gene** | **Loc** | **Regpot** | **Chr** | **Pos** | **BY-Nuc** | **RM-Nuc** | **BY-AA** | **RM-AA** |
| --- | --- | --- | --- | --- | --- | --- | --- | --- | --- |
| 1 | DBP2 | D | 0.5504 | 14 | 416365 | - | T | _ | _ |
| 2 | CYB5 | D | 0.5128 | 14 | 416689 | A | - | _ | _ |
| 3 | DBP2 | D | 0.5106 | 14 | 416700 | T | - | _ | _ |
| 4 | CYB5 | U | 0.5043 | 14 | 417423 | G | A | _ | _ |
| 5 | NOP15 | D | 0.5205 | 14 | 417423 | G | A | _ | _ |
| 6 | NOP15 | C | 0.5403 | 14 | 418170 | A | G | L | L |
| 7 | NOP15 | C | 0.5403 | 14 | 418269 | G | A | Q | Q |
| 8 | NOP15 | C | 0.5403 | 14 | 418302 | A | G | T | T |
| 9 | NOP15 | U | 0.512 | 14 | 418769 | C | T | _ | _ |
| 10 | YNL108C | D | 0.4526 | 14 | 418769 | C | T | _ | _ |
| 11 | YNL108C | D | 0.4526 | 14 | 418805 | C | T | _ | _ |
| 12 | NOP15 | U | 0.512 | 14 | 418805 | C | T | _ | _ |
| 13 | YAF9 | U | 0.5991 | 14 | 420065 | T | C | _ | _ |
| 14 | YNL108C | U | 0.4442 | 14 | 420065 | A | G | _ | _ |
| 15 | YNL108C | U | 0.4442 | 14 | 420183 | A | G | _ | _ |
| 16 | YAF9 | C | 0.597 | 14 | 420183 | T | C | G | G |
| 17 | YAF9 | C | 0.597 | 14 | 420267 | G | C | S | S |
| 18 | YNL108C | U | 0.4442 | 14 | 420267 | C | G | _ | _ |
| 19 | YNL108C | U | 0.4442 | 14 | 420268 | A | G | _ | _ |
| 20 | YAF9 | C | 0.658 | 14 | 420268 | T | C | Y | H |
| 21 | YAF9 | C | 0.6671 | 14 | 420278 | A | C | K | T |
| 22 | YAF9 | C | 0.6481 | 14 | 420326 | C | A | S | Y |
| 23 | YAF9 | C | 0.597 | 14 | 420342 | G | C | P | P |
| 24 | INP52 | C | 0.5699 | 14 | 422748 | A | G | K | E |
| 25 | MET4 | C | 0.7409 | 14 | 429057 | C | G | R | A |
| 26 | MET4 | C | 0.7409 | 14 | 429058 | G | C | R | A |
| 27 | MET4 | C | 0.6198 | 14 | 429061 | G | C | G | A |
| 28 | POL1 | C | 0.5576 | 14 | 431065 | T | C | L | S |
| 29 | POL1 | C | 0.5319 | 14 | 433150 | G | A | R | K |
| 30 | POL1 | D | 0.5118 | 14 | 434509 | A | G | _ | _ |
| 31 | YNL100W | C | 0.578 | 14 | 437957 | G | T | A | S |
| 32 | OCA1 | U | 0.4525 | 14 | 439399 | - | A | _ | _ |
| 33 | RAS2 | D | 0.5703 | 14 | 439399 | - | A | _ | _ |
| 34 | PHO23 | D | 0.4995 | 14 | 441278 | A | - | _ | _ |
| 35 | PHO23 | D | 0.4995 | 14 | 441279 | T | - | _ | _ |
| 36 | PHO23 | D | 0.4995 | 14 | 441280 | A | - | _ | _ |
| 37 | PHO23 | D | 0.4995 | 14 | 441281 | T | - | _ | _ |
| 38 | PHO23 | D | 0.4995 | 14 | 441282 | A | - | _ | _ |
| 39 | PHO23 | D | 0.4995 | 14 | 441283 | T | - | _ | _ |
| 40 | PHO23 | D | 0.4995 | 14 | 441284 | A | - | _ | _ |
| 41 | PHO23 | D | 0.4995 | 14 | 441285 | T | - | _ | _ |
| 42 | PHO23 | D | 0.4995 | 14 | 441286 | A | - | _ | _ |
| 43 | PHO23 | D | 0.4995 | 14 | 441287 | T | - | _ | _ |
| 44 | PHO23 | D | 0.4995 | 14 | 441288 | A | - | _ | _ |
| 45 | PHO23 | D | 0.4995 | 14 | 441289 | C | - | _ | _ |
| 46 | PHO23 | D | 0.4995 | 14 | 441290 | A | - | _ | _ |
| 47 | PHO23 | D | 0.4995 | 14 | 441291 | T | - | _ | _ |
| 48 | PHO23 | D | 0.4995 | 14 | 441292 | C | - | _ | _ |
| 49 | PHO23 | D | 0.4995 | 14 | 441293 | G | - | _ | _ |
| 50 | PHO23 | D | 0.4995 | 14 | 441294 | A | - | _ | _ |
| 51 | PHO23 | D | 0.4995 | 14 | 441295 | T | - | _ | _ |
| 52 | PHO23 | D | 0.4995 | 14 | 441296 | T | - | _ | _ |
| 53 | PHO23 | D | 0.4995 | 14 | 441297 | C | - | _ | _ |
| 54 | PHO23 | D | 0.4995 | 14 | 441298 | T | - | _ | _ |
| 55 | PHO23 | D | 0.4995 | 14 | 441346 | - | C | _ | _ |
| 56 | PHO23 | C | 0.4793 | 14 | 442267 | C | T | L | L |
| 57 | PHO23 | U | 0.4815 | 14 | 442392 | G | T | _ | _ |
| 58 | PHO23 | U | 0.4815 | 14 | 442394 | C | T | _ | _ |
| 59 | PHO23 | U | 0.4512 | 14 | 442496 | A | - | _ | _ |
| 60 | PHO23 | U | 0.4512 | 14 | 442497 | G | - | _ | _ |
| 61 | PHO23 | U | 0.4512 | 14 | 442498 | A | - | _ | _ |
| 62 | PHO23 | U | 0.4512 | 14 | 442499 | A | - | _ | _ |
| 63 | PHO23 | U | 0.4512 | 14 | 442520 | C | T | _ | _ |
| 64 | PHO23 | U | 0.4512 | 14 | 442650 | A | T | _ | _ |
| 65 | PHO23 | U | 0.4512 | 14 | 442655 | T | C | _ | _ |
| 66 | PHO23 | U | 0.4512 | 14 | 442662 | C | T | _ | _ |
| 67 | PHO23 | U | 0.4512 | 14 | 442667 | A | G | _ | _ |
| 68 | PHO23 | U | 0.4512 | 14 | 442718 | G | A | _ | _ |
| 69 | PHO23 | U | 0.4512 | 14 | 442738 | G | T | _ | _ |
| 70 | RPS7B | D | 0.4624 | 14 | 442991 | A | C | _ | _ |
| 71 | RPS7B | D | 0.4624 | 14 | 443036 | G | A | _ | _ |
| 72 | RPS7B | D | 0.4624 | 14 | 443044 | T | A | _ | _ |
| 73 | RPS7B | D | 0.4624 | 14 | 443046 | T | A | _ | _ |
| 74 | RPS7B | D | 0.4624 | 14 | 443048 | T | A | _ | _ |
| 75 | RPS7B | D | 0.4624 | 14 | 443054 | A | C | _ | _ |
| 76 | RPS7B | D | 0.4624 | 14 | 443056 | T | C | _ | _ |
| 77 | RPS7B | D | 0.4624 | 14 | 443060 | G | T | _ | _ |
| 78 | RPS7B | D | 0.4624 | 14 | 443075 | T | A | _ | _ |
| 79 | RPS7B | D | 0.4624 | 14 | 443091 | T | A | _ | _ |
| 80 | RPS7B | D | 0.4624 | 14 | 443097 | T | A | _ | _ |
| 81 | RPS7B | D | 0.4624 | 14 | 443104 | G | C | _ | _ |
| 82 | RPS7B | D | 0.4624 | 14 | 443107 | T | C | _ | _ |
| 83 | RPS7B | D | 0.4624 | 14 | 443114 | T | A | _ | _ |
| 84 | RPS7B | D | 0.4624 | 14 | 443117 | T | G | _ | _ |
| 85 | RPS7B | D | 0.4624 | 14 | 443129 | A | C | _ | _ |
| 86 | YNL095C | C | 0.6006 | 14 | 445768 | C | T | R | C |
| 87 | YNL095C | C | 0.4723 | 14 | 445985 | A | G | S | S |
| 88 | YNL095C | C | 0.5573 | 14 | 446679 | C | G | S | C |
| 89 | APP1 | U | 0.4499 | 14 | 447326 | C | T | _ | _ |
| 90 | APP1 | U | 0.4499 | 14 | 447384 | G | T | _ | _ |
| 91 | APP1 | C | 0.478 | 14 | 447756 | G | T | T | T |
| 92 | APP1 | C | 0.478 | 14 | 448977 | C | G | P | P |
| 93 | APP1 | C | 0.5582 | 14 | 449116 | G | A | A | T |
| 94 | YPT53 | U | 0.5154 | 14 | 449642 | A | G | _ | _ |
| 95 | APP1 | D | 0.4583 | 14 | 449642 | A | G | _ | _ |
| 96 | APP1 | D | 0.4583 | 14 | 449762 | - | A | _ | _ |
| 97 | YPT53 | U | 0.5154 | 14 | 449762 | - | T | _ | _ |
| 98 | YPT53 | U | 0.5154 | 14 | 449762 | - | A | _ | _ |
| 99 | YPT53 | U | 0.5154 | 14 | 449762 | - | G | _ | _ |
| 100 | YPT53 | U | 0.5154 | 14 | 449762 | - | T | _ | _ |
| 101 | YPT53 | U | 0.5154 | 14 | 449762 | - | T | _ | _ |
| 102 | APP1 | D | 0.4583 | 14 | 449762 | - | A | _ | _ |
| 103 | APP1 | D | 0.4583 | 14 | 449762 | - | C | _ | _ |
| 104 | APP1 | D | 0.4583 | 14 | 449762 | - | T | _ | _ |
| 105 | YPT53 | U | 0.5154 | 14 | 449762 | - | T | _ | _ |
| 106 | APP1 | D | 0.4583 | 14 | 449762 | - | A | _ | _ |
| 107 | APP1 | D | 0.4583 | 14 | 449762 | - | G | _ | _ |
| 108 | YPT53 | U | 0.5154 | 14 | 449762 | - | A | _ | _ |
| 109 | YPT53 | U | 0.5154 | 14 | 449762 | - | T | _ | _ |
| 110 | APP1 | D | 0.4583 | 14 | 449762 | - | C | _ | _ |
| 111 | APP1 | D | 0.4583 | 14 | 449762 | - | T | _ | _ |
| 112 | YPT53 | U | 0.5154 | 14 | 449762 | - | A | _ | _ |
| 113 | YPT53 | U | 0.5154 | 14 | 449762 | - | T | _ | _ |
| 114 | YPT53 | U | 0.5154 | 14 | 449762 | - | A | _ | _ |
| 115 | YPT53 | U | 0.5154 | 14 | 449762 | - | A | _ | _ |
| 116 | YPT53 | U | 0.5154 | 14 | 449762 | - | T | _ | _ |
| 117 | APP1 | D | 0.4583 | 14 | 449762 | - | T | _ | _ |
| 118 | YPT53 | U | 0.5154 | 14 | 449762 | - | A | _ | _ |
| 119 | YPT53 | U | 0.5154 | 14 | 449762 | - | A | _ | _ |
| 120 | APP1 | D | 0.4583 | 14 | 449762 | - | T | _ | _ |
| 121 | YPT53 | U | 0.5154 | 14 | 449762 | - | A | _ | _ |
| 122 | APP1 | D | 0.4583 | 14 | 449762 | - | T | _ | _ |
| 123 | YPT53 | U | 0.5154 | 14 | 449762 | - | T | _ | _ |
| 124 | APP1 | D | 0.4583 | 14 | 449762 | - | A | _ | _ |
| 125 | APP1 | D | 0.4583 | 14 | 449762 | - | T | _ | _ |
| 126 | YPT53 | U | 0.5154 | 14 | 449762 | - | T | _ | _ |
| 127 | YPT53 | U | 0.5154 | 14 | 449762 | - | C | _ | _ |
| 128 | APP1 | D | 0.4583 | 14 | 449762 | - | A | _ | _ |
| 129 | YPT53 | U | 0.5154 | 14 | 449762 | - | A | _ | _ |
| 130 | YPT53 | U | 0.5154 | 14 | 449762 | - | G | _ | _ |
| 131 | APP1 | D | 0.4583 | 14 | 449762 | - | T | _ | _ |
| 132 | YPT53 | U | 0.5154 | 14 | 449762 | - | A | _ | _ |
| 133 | APP1 | D | 0.4583 | 14 | 449762 | - | T | _ | _ |
| 134 | YPT53 | U | 0.5154 | 14 | 449762 | - | A | _ | _ |
| 135 | APP1 | D | 0.4583 | 14 | 449762 | - | A | _ | _ |
| 136 | APP1 | D | 0.4583 | 14 | 449762 | - | T | _ | _ |
| 137 | APP1 | D | 0.4583 | 14 | 449762 | - | T | _ | _ |
| 138 | YPT53 | U | 0.5154 | 14 | 449762 | - | T | _ | _ |
| 139 | APP1 | D | 0.4583 | 14 | 449762 | - | A | _ | _ |
| 140 | APP1 | D | 0.4583 | 14 | 449762 | - | C | _ | _ |
| 141 | APP1 | D | 0.4583 | 14 | 449762 | - | A | _ | _ |
| 142 | APP1 | D | 0.4583 | 14 | 449762 | - | A | _ | _ |
| 143 | YPT53 | U | 0.5154 | 14 | 449762 | - | C | _ | _ |
| 144 | YPT53 | U | 0.5154 | 14 | 449762 | - | T | _ | _ |
| 145 | APP1 | D | 0.4583 | 14 | 449762 | - | G | _ | _ |
| 146 | YPT53 | U | 0.5154 | 14 | 449762 | - | C | _ | _ |
| 147 | APP1 | D | 0.4583 | 14 | 449762 | - | T | _ | _ |
| 148 | APP1 | D | 0.4583 | 14 | 449762 | - | A | _ | _ |
| 149 | APP1 | D | 0.4583 | 14 | 449762 | - | A | _ | _ |
| 150 | NST1 | C | 0.6025 | 14 | 453091 | C | A | Q | K |
| 151 | NST1 | C | 0.5451 | 14 | 453939 | C | T | D | D |
| 152 | NST1 | C | 0.5572 | 14 | 454974 | G | A | M | I |
| 153 | RHO2 | U | 0.5282 | 14 | 456297 | T | C | _ | _ |
| 154 | NST1 | D | 0.5253 | 14 | 456297 | T | C | _ | _ |
| 155 | NST1 | D | 0.5253 | 14 | 456357 | T | C | _ | _ |
| 156 | RHO2 | U | 0.5282 | 14 | 456357 | T | C | _ | _ |
| 157 | NST1 | D | 0.5253 | 14 | 456439 | A | G | _ | _ |
| 158 | RHO2 | U | 0.5282 | 14 | 456439 | A | G | _ | _ |
| 159 | RHO2 | D | 0.5367 | 14 | 457245 | A | G | _ | _ |
| 160 | TOP2 | U | 0.5906 | 14 | 457487 | G | A | _ | _ |
| 161 | RHO2 | D | 0.5367 | 14 | 457487 | G | A | _ | _ |
| 162 | TOP2 | C | 0.6903 | 14 | 458516 | G | A | A | T |
| 163 | TOP2 | C | 0.6177 | 14 | 459667 | G | A | L | L |
| 164 | TOP2 | C | 0.6177 | 14 | 460627 | T | C | N | N |
| 165 | TOP2 | C | 0.6999 | 14 | 461487 | A | G | E | G |
| 166 | TOP2 | C | 0.7078 | 14 | 461865 | C | G | S | W |
| 167 | TCB2 | C | 0.5259 | 14 | 462480 | C | T | T | M |
| 168 | TCB2 | C | 0.49 | 14 | 463556 | A | T | I | F |
| 169 | TCB2 | C | 0.4723 | 14 | 463777 | C | T | S | S |
| 170 | TCB2 | C | 0.4723 | 14 | 463942 | T | A | G | G |
| 171 | TCB2 | C | 0.6213 | 14 | 463994 | C | G | R | G |
| 172 | TCB2 | C | 0.4723 | 14 | 464119 | A | T | S | S |
| 173 | TCB2 | C | 0.5126 | 14 | 464235 | G | A | R | K |
| 174 | TCB2 | C | 0.4723 | 14 | 465007 | C | T | P | P |
| 175 | TCB2 | C | 0.5327 | 14 | 465189 | A | T | N | I |
| 176 | YNL086W | U | 0.5085 | 14 | 466105 | G | A | _ | _ |
| 177 | TCB2 | D | 0.4526 | 14 | 466105 | G | A | _ | _ |
| 178 | YNL086W | C | 0.5489 | 14 | 466590 | T | G | I | M |
| 179 | YNL086W | D | 0.517 | 14 | 467030 | A | G | _ | _ |
| 180 | MKT1 | U | 0.5908 | 14 | 467030 | A | G | _ | _ |
| 181 | MKT1 | C | 0.7422 | 14 | 467221 | A | G | D | G |
| 182 | MKT1 | C | 0.7051 | 14 | 468490 | A | G | K | R |
| 183 | MKT1 | C | 0.6179 | 14 | 469226 | A | G | L | L |
| 184 | MKT1 | D | 0.599 | 14 | 469872 | A | G | _ | _ |
| 185 | END3 | D | 0.4818 | 14 | 469872 | T | C | _ | _ |
| 186 | END3 | C | 0.5434 | 14 | 470303 | G | A | D | N |
| 187 | END3 | C | 0.5164 | 14 | 470332 | G | A | S | N |
| 188 | END3 | C | 0.5017 | 14 | 470442 | A | G | K | K |
| 189 | END3 | C | 0.5017 | 14 | 470846 | T | C | L | L |
| 190 | PMS1 | U | 0.5628 | 14 | 473304 | T | C | _ | _ |
| 191 | SAL1 | D | 0.53 | 14 | 473304 | T | C | _ | _ |
| 192 | PMS1 | U | 0.5628 | 14 | 473370 | C | G | _ | _ |
| 193 | SAL1 | D | 0.53 | 14 | 473370 | C | G | _ | _ |
| 194 | SAL1 | D | 0.53 | 14 | 473372 | C | - | _ | _ |
| 195 | PMS1 | U | 0.5628 | 14 | 473372 | C | - | _ | _ |
| 196 | PMS1 | C | 0.5607 | 14 | 473649 | T | C | D | D |
| 197 | YNL080C | D | 0.4629 | 14 | 476711 | A | - | _ | _ |
| 198 | SWS2 | U | 0.6131 | 14 | 476711 | A | - | _ | _ |
| 199 | TPM1 | C | 0.6105 | 14 | 478702 | T | C | D | D |
| 200 | NIS1 | C | 0.6496 | 14 | 480473 | A | C | R | S |
| 201 | APJ1 | U | 0.4442 | 14 | 481082 | C | T | _ | _ |
| 202 | NIS1 | D | 0.5649 | 14 | 481082 | C | T | _ | _ |
| 203 | APJ1 | C | 0.5638 | 14 | 481897 | A | G | E | G |
| 204 | MKS1 | C | 0.6366 | 14 | 484905 | A | G | E | G |
| 205 | MKS1 | D | 0.5285 | 14 | 485550 | T | C | _ | _ |
| 206 | IMP4 | U | 0.499 | 14 | 485550 | T | C | _ | _ |
| 207 | MLF3 | D | 0.4567 | 14 | 486553 | - | T | _ | _ |
| 208 | IMP4 | D | 0.517 | 14 | 486575 | - | A | _ | _ |
| 209 | MLF3 | C | 0.4885 | 14 | 486860 | A | T | E | D |
| 210 | IMP4 | D | 0.4771 | 14 | 486860 | T | A | _ | _ |
| 211 | MLF3 | C | 0.5072 | 14 | 487251 | C | G | T | S |
| 212 | MLF3 | C | 0.5565 | 14 | 487819 | G | A | E | K |
| 213 | MLF3 | C | 0.5565 | 14 | 487936 | G | A | E | K |
| 214 | MSK1 | C | 0.5659 | 14 | 488599 | G | C | E | D |
| 215 | LAT1 | C | 0.5971 | 14 | 492376 | A | G | I | V |
| 216 | LAT1 | D | 0.6115 | 14 | 493071 | A | - | _ | _ |
| 217 | TOM7 | U | 0.4598 | 14 | 493072 | A | - | _ | _ |
| 218 | LAT1 | D | 0.5728 | 14 | 493072 | A | - | _ | _ |
| 219 | TOM7 | U | 0.4598 | 14 | 493073 | A | - | _ | _ |
| 220 | LAT1 | D | 0.5728 | 14 | 493334 | T | G | _ | _ |
| 221 | TOM7 | U | 0.4902 | 14 | 493334 | T | G | _ | _ |
