# Supplementary material for: Learning a Prior on Regulatory Potential from eQTL Data
Source: PLoS Genet. 2009 Jan 30;5(1):e1000358. doi: 10.1371/journal.pgen.1000358 (PMC2627940; doi:10.1371/journal.pgen.1000358)
Supplement: Table S10 — Microscopy quantitation. To quantitate the extent to which Puf3 protein (assayed by Puf3-GFP fluorescence, green) co-localized with P-bodies (assayed as Edc3-tdimer2 fluorescence, red), we counted the number of spots that exhibited visually detectable green and/or red fluorescence. The number of Puf3 spots reached a maximum at approximately 20 minutes post induction. We cannot rule out the possibility that differences in rates of spot formation could be due to differences in the properties of GFP and tdimer2 because our inability to detect Puf3 fused to tdimer2 or other versions of red fluorescent protein (Dudley and Drubin, unpublished results) prevented us from swapping the fluorescent protein tags. (0.05 MB DOC) [file pgen.1000358.s023.doc]

| **Time post induction** | **Edc3 & Puf3** | **Edc3 only** | **Puf3 only** | **Total Spots** | **% P-bodies + Puf3*** |
| --- | --- | --- | --- | --- | --- |
| ~12 min. | 10 | 46 | 0 | 56 | 18% |
| ~14 min. | 21 | 55 | 4 | 80 | 28% |
| ~20 min. | 69 | 6 | 4 | 79 | 92% |
| ~22 min. | 58 | 10 | 10 | 78 | 85% |

***% P-bodies + Puf3 = Edc3 & Puf3/ (Edc3 & Puf3 + Edc3 only)**
