# Supplementary material for: Learning a Prior on Regulatory Potential from eQTL Data
Source: PLoS Genet. 2009 Jan 30;5(1):e1000358. doi: 10.1371/journal.pgen.1000358 (PMC2627940; doi:10.1371/journal.pgen.1000358)
Supplement: Table S11 — Deletion arrays. To evaluate the dependence of the remaining target genes on Oaf1, we examined a published microarray dataset [29] comparing RNA expression oaf1Δ to a wild-type (BY) strain in the presence of oleate (an inducing condition). This dataset also included an estimate of the likelihood of differential expression [74]. We sorted RNA expression levels by the log10 ratios and filtered for λ values greater than 36.23 to arrive at the top 1% (63) most significantly down regulated genes. (0.1 MB DOC) [file pgen.1000358.s024.doc]

| Oaf1 responsive genes (log 10 ratio < -0.2 and λ > 36.23) | | | | | |  | | | |
| --- | --- | --- | --- | --- | --- | --- | --- | --- | --- |
|  |  | del_ADR1_vs_WT | | del_OAF3_vs_WT | | del_OAF1_vs_WT | | del_PIP2_vs_WT | |
| Gene | Alias | log_10_ ratio |  | log_10_ ratio |  | log_10_ ratio |  | log_10_ ratio |  |
| YGL205W | POX1 | -1.6216 | 188.10 | 0.106 | 12.99 | -1.3535 | 201.05 | -1.2532 | 205.22 |
| YPL095C | YPL095C | -0.0931 | 11.53 | 0.0814 | 8.61 | -1.1574 | 186.60 | -1.0186 | 180.40 |
| YDR256C | CTA1 | -1.2848 | 176.61 | 0.0919 | 5.87 | -0.9531 | 169.80 | -0.5015 | 119.83 |
| YKR009C | FOX2 | -1.0278 | 176.97 | 0.0114 | 0.16 | -0.8528 | 169.16 | -0.7064 | 152.63 |
| YER015W | FAA2 | -0.8704 | 159.68 | 0.1142 | 12.36 | -1.0084 | 165.01 | -0.7376 | 164.08 |
| YLR284C | ECI1 | -0.8608 | 140.99 | -0.0372 | 1.88 | -0.9857 | 164.66 | -0.625 | 140.29 |
| YNL202W | SPS19 | -1.0524 | 175.82 | 0.1876 | 30.72 | -0.8347 | 160.40 | -0.6679 | 143.36 |
| YJR019C | TES1 | -0.7062 | 129.58 | -0.0706 | 5.97 | -0.705 | 139.85 | -0.5803 | 133.57 |
| YAL051W | OAF1 | 0.0086 | 0.06 | 0.0458 | 1.03 | -1.1457 | 129.09 | -0.0018 | 0.00 |
| YDL037C | YDL037C | 0.0555 | 1.94 | -0.0808 | 2.56 | -1.0731 | 127.29 | -0.0658 | 4.38 |
| YNL009W | IDP3 | -0.7794 | 157.59 | -0.0559 | 4.67 | -0.6166 | 125.54 | -0.2919 | 71.85 |
| YDL038C | YDL038C | 0.082 | 4.44 | 0.218 | 14.94 | -0.7052 | 117.75 | -0.2813 | 67.28 |
| YOR084W | YOR084W | -0.3808 | 87.58 | 0.1152 | 10.17 | -0.5134 | 115.98 | -0.4862 | 121.26 |
| YHR140W | YHR140W | -0.4054 | 105.46 | 0.1014 | 10.11 | -0.5721 | 111.79 | -0.5512 | 120.26 |
| YGR035C | YGR035C | 0.1549 | 16.72 | 0.0378 | 1.20 | -0.7285 | 110.06 | -0.3186 | 73.66 |
| YHR160C | PEX18 | -0.0092 | 0.08 | 0.0756 | 6.67 | -0.4837 | 105.39 | -0.4369 | 104.20 |
| YJL153C | INO1 | -0.0949 | 10.09 | -0.0592 | 4.00 | -0.538 | 100.24 | -0.0497 | 5.24 |
| YOR190W | SPR1 | 0.0418 | 0.34 | 0.2296 | 0.98 | -0.5803 | 97.76 | 0.1925 | 10.07 |
| YKL188C | PXA2 | -0.2999 | 61.80 | 0.0762 | 2.61 | -0.4012 | 90.58 | -0.2673 | 62.31 |
| YDL039C | PRM7 | 0.2569 | 16.42 | 0.0547 | 0.64 | -0.6345 | 83.79 | -0.3198 | 66.98 |
| YJL196C | ELO1 | -0.2092 | 46.87 | 0.0458 | 1.60 | -0.4103 | 81.14 | 0.0004 | 0.00 |
| YOR229W | WTM2 | 0.0837 | 9.40 | 0.0023 | 0.01 | -0.4273 | 77.50 | 0.0912 | 12.22 |
| YOR273C | TPO4 | -0.2615 | 50.75 | 0.0887 | 8.54 | -0.3111 | 70.58 | -0.2842 | 75.59 |
| YOL156W | HXT11 | 0.0159 | 0.12 | 0.3123 | 1.35 | -0.3824 | 70.21 | -0.0334 | 0.77 |
| YOR180C | DCI1 | -0.9925 | 148.30 | 0.0981 | 8.30 | -0.4647 | 65.56 | -0.586 | 96.64 |
| YMR018W | YMR018W | -0.3502 | 15.38 | 0.1426 | 0.22 | -0.4361 | 63.60 | -0.1669 | 8.54 |
| YPL112C | PEX25 | -0.0805 | 6.74 | 0.0325 | 0.94 | -0.2889 | 62.87 | -0.0407 | 2.47 |
| YJL200C | YJL200C | 0.02 | 0.57 | -0.1081 | 5.82 | -0.295 | 60.45 | -0.0194 | 0.74 |
| YOR363C | PIP2 | -0.2386 | 51.91 | -0.2123 | 29.25 | -0.3227 | 58.82 | -0.4024 | 62.79 |
| YOR315W | YOR315W | 0.1619 | 9.08 | -0.1911 | 13.95 | -0.3326 | 56.26 | -0.0419 | 1.47 |
| YDR244W | PEX5 | -0.1321 | 27.52 | -0.0459 | 2.09 | -0.2598 | 56.06 | -0.1095 | 20.48 |
| YAL025C | MAK16 | 0.1279 | 19.47 | -0.3105 | 55.91 | -0.2357 | 53.58 | -0.0622 | 4.15 |
| YNL205C | YNL205C | -0.5706 | 92.56 | 0.0798 | 1.65 | -0.3828 | 53.54 | -0.3841 | 83.68 |
| YKL201C | MNN4 | -0.0246 | 0.51 | -0.0793 | 1.33 | -0.2558 | 53.48 | -0.0264 | 1.18 |
| YDR281C | PHM6 | 0.1173 | 14.97 | 0.05 | 2.93 | -0.2057 | 49.72 | -0.1324 | 31.55 |
| YOR017W | PET127 | 0.0409 | 0.62 | -0.2263 | 14.15 | -0.2702 | 49.05 | -0.0152 | 0.16 |
| YIL160C | POT1 | -0.3765 | 90.14 | 0.1003 | 6.31 | -0.2401 | 48.48 | -0.2188 | 48.29 |
| YOR101W | RAS1 | 0.0829 | 9.07 | -0.0459 | 0.65 | -0.2584 | 47.88 | -0.0188 | 0.55 |
| YBL008W | HIR1 | -0.1474 | 29.17 | -0.0553 | 3.16 | -0.2467 | 47.62 | -0.1475 | 36.30 |
| YOR105W | YOR105W | 0.1398 | 12.68 | 0.3021 | 5.78 | -0.2592 | 46.44 | 0.0039 | 0.02 |
| YLR285W | YLR285W | -0.055 | 3.26 | 0.0593 | 1.45 | -0.2779 | 45.43 | -0.1723 | 32.31 |
| YPL147W | PXA1 | -0.7142 | 131.99 | -0.0403 | 1.59 | -0.2143 | 45.30 | -0.1176 | 22.79 |
| YDR083W | RRP8 | 0.2992 | 43.65 | -0.0513 | 1.63 | -0.2202 | 44.93 | -0.0648 | 5.44 |
| YDL110C | YDL110C | -0.1287 | 20.91 | -0.0046 | 0.03 | -0.2267 | 44.60 | -0.1329 | 32.67 |
| YNR050C | LYS9 | -0.4441 | 98.33 | -0.0166 | 0.40 | -0.2165 | 44.12 | -0.298 | 72.67 |
| YMR210W | YMR210W | -0.1202 | 13.48 | 0.0219 | 0.17 | -0.2344 | 43.36 | -0.0625 | 3.26 |
| YBL095W | YBL095W | -0.0985 | 9.45 | 0.0365 | 1.65 | -0.2347 | 42.35 | -0.0428 | 2.30 |
| YGR079W | YGR079W | 0.4328 | 73.55 | 0.2077 | 7.43 | -0.2315 | 41.37 | 0.1639 | 23.57 |
| YLR002C | NOC3 | 0.0846 | 7.80 | 0.0253 | 0.45 | -0.2033 | 41.06 | -0.0288 | 1.43 |
| YLR197W | SIK1 | 0.1748 | 22.04 | -0.1285 | 20.92 | -0.2187 | 40.99 | -0.0439 | 3.19 |
| YBR154C | RPB5 | 0.0679 | 6.56 | -0.1376 | 23.40 | -0.2074 | 40.74 | -0.0272 | 1.29 |
| YOR209C | NPT1 | 0.0952 | 11.58 | 0.0639 | 4.61 | -0.2098 | 40.53 | 0.0641 | 6.47 |
| YOR268C | YOR268C | 0.1101 | 0.53 | 0 | 0.00 | -0.3208 | 40.18 | 0.0871 | 0.31 |
| YNL182C | IPI3 | 0.2267 | 36.27 | -0.0606 | 2.92 | -0.24 | 39.55 | 0.0232 | 0.73 |
| YDR071C | YDR071C | 0.0374 | 1.99 | 0.0452 | 2.77 | -0.2201 | 37.60 | -0.0691 | 9.14 |
| YJL179W | PFD1 | 0.0896 | 8.74 | -0.1001 | 4.76 | -0.2301 | 37.36 | 0.0142 | 0.38 |
| YGR272C | YGR272C | 0.1932 | 20.77 | -0.1731 | 8.83 | -0.2527 | 37.33 | 0.032 | 1.28 |
| YHR066W | SSF1 | 0.296 | 52.42 | -0.0594 | 0.43 | -0.2332 | 37.02 | 0.048 | 3.36 |
| YGR081C | YGR081C | 0.0347 | 0.98 | -0.0189 | 0.05 | -0.2446 | 36.88 | -0.0816 | 9.16 |
| YML093W | UTP14 | 0.1592 | 19.84 | -0.1211 | 4.56 | -0.2333 | 36.40 | 0.0243 | 0.81 |
