# Supplementary material for: Learning a Prior on Regulatory Potential from eQTL Data
Source: PLoS Genet. 2009 Jan 30;5(1):e1000358. doi: 10.1371/journal.pgen.1000358 (PMC2627940; doi:10.1371/journal.pgen.1000358)
Supplement: Table S12 — Regulatory Gene Ontology (GO) categories. For constructing regulatory features, we characterized each gene based on GO categories. Different organisms have different sets of categories that are relevant to the regulatory processes. Therefore, we used different lists for yeast and human data. (A) We constructed a list of 76 biological process and 11 molecular function Gene Ontology (GO) categories that might be related to gene regulatory functions in yeast. (B) For human data, we used a list of 48 GO Slim biological process categories. (0.09 MB DOC) [file pgen.1000358.s025.doc]

# A. Yeast

| **GO ID** | **Ontology** | **Definition** |
| --- | --- | --- |
| GO:0009060 | Biological process | Aerobic respiration |
| GO:0009308 |  | Amine metabolic process |
| GO:0006519 |  | Amino acid and derivative metabolic process |
| GO:0006725 |  | Aromatic compound metabolic process |
| GO:0016051 |  | Carbohydrate biosynthetic process |
| GO:0005975 |  | Carbohydrate metabolic process |
| GO:0008643 |  | Carbohydrate transport |
| GO:0019752 |  | Carboxylic acid metabolic process |
| GO:0007154 |  | Cell communication |
| GO:0007049 |  | Cell cycle |
| GO:0000075 |  | Cell cycle checkpoint |
| GO:0045333 |  | Cellular respiration |
| GO:0007047 |  | Cell wall organization and biogenesis |
| GO:0016568 |  | Chromatin modification |
| GO:0006338 |  | Chromatin remodeling |
| GO:0051276 |  | Chromosome organization and biogenesis |
| GO:0007010 |  | Cytoskeleton organization and biogenesis |
| GO:0032502 |  | Developmental process |
| GO:0006259 |  | DNA metabolic process |
| GO:0006281 |  | DNA repair |
| GO:0008204 |  | Ergosterol metabolic process |
| GO:0051234 |  | Establishment of localization |
| GO:0006006 |  | Glucose metabolic process |
| GO:0006096 |  | Glycolysis |
| GO:0009100 |  | Glycoprotein metabolic process |
| GO:0048193 |  | Golgi vesicle transport |
| GO:0040007 |  | Growth |
| GO:0019318 |  | Hexose metabolic process |
| GO:0016570 |  | Histone modification |
| GO:0042592 |  | Homeostatic process |
| GO:0006629 |  | Lipid metabolic process |
| GO:0016044 |  | Membrane organization and biogenesis |
| GO:0006555 |  | Methionine metabolic process |
| GO:0006839 |  | Mitochondrial transport |
| GO:0000278 |  | Mitotic cell cycle |
| GO:0016071 |  | MRNA metabolic process |
| GO:0044270 |  | Nitrogen compound catabolic process |
| GO:0006807 |  | Nitrogen compound metabolic process |
| GO:0051169 |  | Nuclear transport |
| GO:0006119 |  | Oxidative phosphorylation |
| GO:0043687 |  | Post-translational protein modification |
| GO:0006457 |  | Protein folding |
| GO:0008104 |  | Protein localization |
| GO:0006464 |  | Protein modification process |
| GO:0015031 |  | Protein transport |
| GO:0016567 |  | Protein ubiquitination |
| GO:0006508 |  | Proteolysis |
| GO:0006090 |  | Pyruvate metabolic process |
| GO:0051726 |  | Regulation of cell cycle |
| GO:0010468 |  | Regulation of gene expression |
| GO:0040029 |  | Regulation of gene expression, epigenetic |
| GO:0019222 |  | Regulation of metabolic process |
| GO:0051252 |  | Regulation of RNA metabolic process |
| GO:0045449 |  | Regulation of transcription |
| GO:0006417 |  | Regulation of translation |
| GO:0006974 |  | Response to DNA damage stimulus |
| GO:0042493 |  | Response to drug |
| GO:0050896 |  | Response to stimulus |
| GO:0006950 |  | Response to stress |
| GO:0042255 |  | Ribosome assembly |
| GO:0042254 |  | Ribosome biogenesis and assembly |
| GO:0032774 |  | RNA biosynthetic process |
| GO:0016070 |  | RNA metabolic process |
| GO:0009451 |  | RNA modification |
| GO:0006396 |  | RNA processing |
| GO:0046903 |  | Secretion |
| GO:0007165 |  | Signal transduction |
| GO:0030435 |  | Sporulation |
| GO:0006790 |  | Sulfur metabolic process |
| GO:0032200 |  | Telomere organization and biogenesis |
| GO:0006350 |  | Transcription |
| GO:0006412 |  | Translation |
| GO:0006413 |  | Translational initiation |
| GO:0006810 |  | Transport |
| GO:0006399 |  | TRNA metabolic process |
| GO:0006512 |  | Ubiquitin cycle |
| GO:0016887 | Molecular function | ATPase activity |
| GO:0003677 |  | DNA binding |
| GO:0030234 |  | Enzyme regulator activity |
| GO:0016301 |  | Kinase activity |
| GO:0016874 |  | Ligase activity |
| GO:0016614 |  | Oxidoreductase activity, acting on CH-OH group of donors |
| GO:0004672 |  | Protein kinase activity |
| GO:0003723 |  | RNA binding |
| GO:0003735 |  | Structural constituent of ribosome |
| GO:0030528 |  | Transcription regulator activity |
| GO:0005215 |  | Transporter activity |

B. Human

| **GO ID** | **Ontology** | **Description** |
| --- | --- | --- |
| GO:0000003 | Biological process | reproduction |
| GO:0005975 |  | carbohydrate metabolic process |
| GO:0006091 |  | generation of precursor metabolites and energy |
| GO:0006118 |  | electron transport |
| GO:0006139 |  | nucleobase, nucleoside, nucleotide and nucleic acid metabolic process |
| GO:0006259 |  | DNA metabolic process |
| GO:0006350 |  | transcription |
| GO:0006412 |  | translation |
| GO:0006464 |  | protein modification process |
| GO:0006519 |  | amino acid and derivative metabolic process |
| GO:0006629 |  | lipid metabolic process |
| GO:0006810 |  | transport |
| GO:0006811 |  | ion transport |
| GO:0006950 |  | response to stress |
| GO:0006996 |  | organelle organization and biogenesis |
| GO:0007005 |  | mitochondrion organization and biogenesis |
| GO:0007010 |  | cytoskeleton organization and biogenesis |
| GO:0007028 |  | cytoplasm organization and biogenesis |
| GO:0007049 |  | cell cycle |
| GO:0007154 |  | cell communication |
| GO:0007165 |  | signal transduction |
| GO:0007267 |  | cell-cell signaling |
| GO:0007275 |  | multicellular organismal development |
| GO:0007610 |  | behavior |
| GO:0008037 |  | cell recognition |
| GO:0008152 |  | metabolic process |
| GO:0008219 |  | cell death |
| GO:0008283 |  | cell proliferation |
| GO:0009056 |  | catabolic process |
| GO:0009058 |  | biosynthetic process |
| GO:0009605 |  | response to external stimulus |
| GO:0009607 |  | response to biotic stimulus |
| GO:0009628 |  | response to abiotic stimulus |
| GO:0009653 |  | anatomical structure morphogenesis |
| GO:0009719 |  | response to endogenous stimulus |
| GO:0009790 |  | embryonic development |
| GO:0015031 |  | protein transport |
| GO:0016043 |  | cellular component organization and biogenesis |
| GO:0016049 |  | cell growth |
| GO:0016265 |  | death |
| GO:0019538 |  | protein metabolic process |
| GO:0019725 |  | cellular homeostasis |
| GO:0019748 |  | secondary metabolic process |
| GO:0030154 |  | cell differentiation |
| GO:0040007 |  | growth |
| GO:0040029 |  | regulation of gene expression, epigenetic |
| GO:0044238 |  | primary metabolic process |
| GO:0050789 |  | regulation of biological process |
