# Supplementary material for: Learning a Prior on Regulatory Potential from eQTL Data
Source: PLoS Genet. 2009 Jan 30;5(1):e1000358. doi: 10.1371/journal.pgen.1000358 (PMC2627940; doi:10.1371/journal.pgen.1000358)
Supplement: Table S13 — Functional interactions between the PTR module members and related genes. Data and references were obtained from the Saccharomyces Genome Database (SGD). (0.09 MB DOC) [file pgen.1000358.s026.doc]

| **PTR member** | **Interaction** | **Interaction Type** | **Reference** |
| --- | --- | --- | --- |
| **DHH1** | **KEM1** | co-immunoprecipitation | [26] |
| **KEM1** | **DHH1** | co-immunoprecipitation | [26] |
| **KEM1** | **TUB1** | synthetic slow growth | [27] |
| **KEM1** | **PDS5** | large scale mass spectrometry | [28] |
| **GCN20** | **GCN1** | co-immunoprecipitation | [29] |
| **GCN1** | **GCN20** | co-immunoprecipitation | [29] |
| **GCN1** | **TUB1** | large scale mass spectrometry | [30] |
| **BLM3 (BLM10)** | **ECM29** | synthetic slow growth | [31] |
| **SIM1** | **PAB1** | 2-hybrid (not high-throughput) | [32] |
| **ECM29** | **BLM10** | synthetic slow growth | [31] |
| **PDS5** | **KEM1** | large scale mass spectrometry | [28] |
| **PDS5** | **PBP1** | large scale mass spectrometry | [28] |
| **PDS5** | **TOP2** | genetic suppression (not large scale) | [33] |
| **POL2** | **PUB1** | large scale mass spectrometry | [30] |
| **TUB1** | **GCN1** | large scale mass spectrometry | [30] |
| **TUB1** | **KEM1** | synthetic slow growth | [27] |
| **TUB1** | **P-bodies** | co-localization | [34] |
| **TOP2** | **PAT1** | 2-hybrid (not high-throughput) | [35] |
| **TOP2** | **PDS5** | genetic suppression (not large scale) | [33] |
