# Supplementary material for: Learning a Prior on Regulatory Potential from eQTL Data
Source: PLoS Genet. 2009 Jan 30;5(1):e1000358. doi: 10.1371/journal.pgen.1000358 (PMC2627940; doi:10.1371/journal.pgen.1000358)
Supplement: Table S14 — Genetic Interactions in the EMAP data. (0.7 MB DOC) [file pgen.1000358.s027.doc]

| **Array genes** | **PUF3** | **PUF4** | **GCN1** | **EDC3** | **LSM1** | **PAT1** |
| --- | --- | --- | --- | --- | --- | --- |
| **EFB1 - DAMP** | 0.527842 | -1.525518 | 0.558663 | 0.766653 | -10.137873 | -8.412695 |
| **DBP5 - DAMP** | 0.95759 | -3.881367 | 0.072888 | 0.898365 | -13.896591 | -11.76757 |
| **PUF4** |  |  | -0.264768 | 0.61837 | -10.671212 |  |
| **GLC7** | -0.627505 | -0.529125 | 0.78706 | 0.463418 | -8.456575 | -9.880297 |
| **TOP1** | -0.768764 | -1.290286 | -1.041427 | -0.80786 | -7.976772 | -12.409548 |
| **SRC1** | -1.517608 | 0.928093 | -2.197493 | -0.014577 | -4.049004 | -6.435484 |
| **NPL3** |  |  | -4.284749 | 0.133655 | -8.66096 |  |
| **NPL3 - NEW** |  | -2.443076 | -0.249044 | -4.901109 | -2.909646 | -1.390657 |
| **SAC3** | 1.08436 | -9.56301 | -1.171936 | 1.288001 | -5.875526 | -6.207295 |
| **THP2** | 0.173568 | -10.480937 | 1.109516 | -0.506279 | -7.737638 | -8.163097 |
| **SEM1** | -0.039785 | -3.692459 | -7.752256 | 0.115517 | -12.423284 | -9.907219 |
| **DOA1** |  |  | -11.206227 | -0.31501 | -3.448572 | -10.599689 |
| **SAC3 - NEW** | -2.916968 |  | -2.165074 | 1.266229 | -4.648087 |  |
| **NRP1** | -0.203194 | -1.455799 | -2.468161 | -0.6096 | -6.19067 | -3.980315 |
| **EFT2** | -0.165052 | 0.282484 | -0.604997 | -0.339453 | -8.932875 | -9.931866 |
| **SKI8** | 0.227656 | 0.295821 |  | 0.020892 | -5.910802 | -8.43947 |
| **SKI2** | 0.4642 | 0.820344 | 0.116765 | 0.121901 | -9.464448 | -6.9275 |
| **SKI3** | 0.52074 | 0.129311 | -0.502945 | 0.92179 | -5.480771 | -7.608786 |
| **SKI7** | 0.375749 | 0.298508 | 0.140654 | 0.780566 | -8.206538 | -8.363469 |
| **DST1** | -1.032796 |  | 0.139621 | -0.62289 | -13.193367 | -13.96604 |
| **HIR2** | 1.203299 | -2.223504 | -1.835969 | 0.664976 | -12.238746 | -16.145064 |
| **SEH1** | 0.618376 | -0.371333 | -0.457788 | -0.696761 | -5.075332 | -6.161869 |
| **TEF2** | 2.583578 | -0.347855 | 0.458814 | 1.808599 | -1.477255 | 0.056638 |
| **KAP123** |  |  | 0.759939 | 0.184934 | -4.287457 |  |
| **DEG1** | -2.237472 | -0.900912 | 2.112759 | 0.410428 | -6.470328 | -6.394973 |
| **DEG1 - NEW** | -0.434685 | 1.927892 | 0.534196 | -1.778898 | -0.01649 | -0.835063 |
| **TEF1** | -0.835076 | 0.257011 | 0.421706 | -0.194487 | -2.619198 | -2.413591 |
| **ARC1** | -1.849756 | -1.951828 | -0.791543 | -0.159625 | -11.654254 | -7.724732 |
| **HPR1** |  |  | -0.40023 | 0.068306 | -7.655553 |  |
| **RXT2** |  |  | -0.541963 | 0.921871 | -1.362065 | -2.099109 |
| **PHO23** |  |  | 1.211754 | -0.191975 | -0.854083 | -2.30267 |
| **SAP30** | 1.928838 | -3.349711 | -0.785815 | 1.13056 | -4.633641 | -2.044378 |
| **APQ12** | 0.452428 | -2.098777 | 0.858529 | 1.375068 | 0.325385 | 1.045203 |
| **MEF1** |  |  | 1.699887 | -4.783876 | 3.68021 |  |
| **NGR1** | -2.120474 | 0.677809 | 2.256895 | -0.317108 | 0.651618 | 1.804936 |
| **SIF2** | 0.314959 | 0.524583 | 3.395538 | 0.274481 | 0.981654 | 1.996271 |
| **HOS2** | 0.467473 | 0.40534 |  | -1.274238 | -0.301266 | 0.990564 |
| **SET3** |  |  | 1.983104 | -0.70682 | 0.28889 | 0.136759 |
| **CDC73** | -0.73292 | -3.862769 | 0.627201 | -0.149241 | -3.616735 | -2.357137 |
| **LEO1** |  |  | 2.95937 | 0.363053 | -1.602394 | -3.112266 |
| **PRP4 - DAMP** |  | -1.751173 | -1.092471 | 0.113019 | -1.320961 | -1.092702 |
| **GBP2** |  |  | 1.156263 | 0.629673 | -1.682713 | -1.063313 |
| **RTT103** |  |  | 1.928587 | 0.20368 | 0.965256 | 2.380969 |
| **SSD1** | 0.300704 | -1.223122 | -0.689197 | -0.14446 | -2.059779 | -1.847826 |
| **SWR1** |  |  | -3.194152 | -0.082437 | -0.024622 | -3.871545 |
| **VPS71** |  |  | -4.023843 | -0.053956 | -2.357226 | -5.016098 |
| **HTZ1** | -1.694585 | -1.929488 | -2.901216 | -1.081533 | -4.891693 | -6.683124 |
| **HTZ1 - NEW** | 0.287909 | 2.051369 | -1.879446 |  | -2.609278 |  |
| **EAF5** | -0.173046 | 0.127232 | -1.684111 |  | -3.734801 | -2.563881 |
| **EAF6** | -0.143796 | -0.142011 | -0.54372 | -0.24266 | -0.446307 | -1.499051 |
| **EAF7** |  |  | -0.246368 | -0.117842 | -0.468312 | 0.137165 |
| **EAF3** | -0.562822 | 0.013167 | -0.639882 | -1.991561 | -0.510128 | -0.688769 |
| **KAP122** |  |  | -1.511083 | 0.247606 | -5.859591 |  |
| **SOH1** |  |  | -4.38654 | -0.962009 | -1.727718 | -7.073886 |
| **CSE2** |  |  | -1.569066 | -0.086809 | -8.790267 |  |
| **MED1** |  |  | 0.137261 | 0.285008 | -6.299786 | -7.367731 |
| **TAD3 - DAMP** | -0.798668 | -0.59751 | -2.720886 | -1.292505 | -8.005021 | -5.85832 |
| **SWD3** |  |  | -2.570944 | 0.230329 | 1.113053 | 0.533832 |
| **BRE1** | -1.095084 | 1.269719 | 0.611906 | -0.618244 | 1.170896 | 1.870087 |
| **GIM4** | -0.532634 | 1.679931 | 1.951811 |  | 3.173112 | 2.511278 |
| **YKE2** | -0.626331 | 0.113529 | 1.345652 | -1.522802 | 2.359838 | -0.495904 |
| **GIM3** |  |  | 0.321578 | 0.475477 | 2.066732 | 0.368296 |
| **GIM5** |  |  | -0.176182 | 0.518497 | 1.095709 | 2.2873 |
| **GIM5 - NEW** | -2.665248 | 3.047457 | 3.178449 | 0.449735 | 1.332314 | 4.535017 |
| **RIC1** |  |  | -4.333267 | -0.698312 | 4.95114 | 5.873447 |
| **YPT6** |  |  | -2.333466 | -0.144313 | 3.818723 | 5.073647 |
| **ECM1** | 0.177018 | 0.004011 | 0.171218 | -0.03962 | 1.701936 | 1.290432 |
| **SLX9** |  |  | -0.593841 | -0.71765 | 2.57729 |  |
| **ASM4** | 0.153473 | 0.560163 | 0.698287 | 1.0332 | 0.317508 | 0.890567 |
| **NUP53** | 0.280031 | 0.233993 | 1.42099 | 1.357151 | -1.420117 | -0.833804 |
| **NUP2** | 1.069168 | -0.606389 | 0.875084 | 1.946716 | -0.758433 | -1.649031 |
| **NUP188** |  |  | -0.619648 | 0.565196 | -5.803912 |  |
| **SRP1 - DAMP** | -0.879458 | -2.690977 | -0.81001 | -1.311364 | 0.174952 | -1.216121 |
| **NUP57 - DAMP** | -1.38291 | -1.83895 | -0.455171 | -0.397721 | -4.959945 | -3.339274 |
| **MEX67** | -0.980518 | -0.372644 | 0.988929 | -0.581262 | -3.526332 | -3.848623 |
| **POM34** | -0.825597 | 0.627802 | 1.166931 | 0.29422 | 0.730983 | 0.730148 |
| **POM152** |  |  | 0.38078 | 0.682143 | 0.392564 |  |
| **MOG1** | 0.260432 | -0.645623 | 2.091733 | -0.463494 | 0.183317 | -0.380961 |
| **NUP120** | 0.179595 |  | 3.579235 | -1.144177 | -6.278388 | 2.37032 |
| **NUP120 - NEW** | -0.364507 | -3.751476 | -0.589994 | -0.565121 | -3.2876 | -2.948544 |
| **NUP170** |  |  | 0.098358 | 0.984095 | -1.598956 |  |
| **CSE1** | -0.956086 | 0.523264 | 0.136983 | 0.037416 | 0.021867 | 0.195892 |
| **NUP42** | -0.382884 | -0.002442 | 1.061451 | -0.433111 | -0.40328 | -0.915141 |
| **REF2** | 0.110819 | 0.45332 | 0.44804 | 0.639808 | -0.206509 | 0.699684 |
| **ARX1** |  | 0.348318 | 1.460426 | 1.383363 | 0.896789 | 1.090429 |
| **RPB4** |  |  | 1.969125 | -1.048702 |  | -1.055183 |
| **CAK1 - NEW** | -0.921986 |  | -0.165746 | 2.715714 | 0.023405 | -0.690744 |
| **HMT1** | 0.073302 | 0.47761 | -0.616235 | 0.597211 | -2.491098 | -1.157115 |
| **SNU56 - DAMP** | -0.569432 | 0.287109 | -0.737121 | 0.368328 | -0.976454 | -0.343161 |
| **KRS1 - DAMP** | 0.171363 | 0.482682 | -0.117567 | 0.343785 | -0.059757 | -0.077057 |
| **TIF35 - DAMP** | -0.258471 | -0.518737 | -0.661308 | 0.391323 | 0.201043 | -0.23241 |
| **YHC1 - DAMP** | 0.616444 | 0.583514 | 0.695731 | 0.864086 | 0.771887 | 0.616862 |
| **ECM2** |  |  | 0.522646 | -0.029943 | -0.034749 |  |
| **YPR152C** |  |  | 1.125857 | 0.116695 | -0.286315 |  |
| **CWC21** | 0.371437 | 0.633417 | -1.908503 | 1.406471 | -1.275095 | 0.223832 |
| **ISY1** | 0.693594 | 0.334785 | -0.327226 | 0.053041 | -3.282277 | -0.936937 |
| **SYF2** | 0.118245 | 0.029611 | -0.452221 | 0.366984 | 0.78162 | -0.170383 |
| **NTC20** |  |  | 0.095752 | -0.177969 | 0.946655 |  |
| **MLP2** |  |  | 0.245375 | 0.374839 | -0.0964 |  |
| **CSN12** |  |  | 0.657874 | 1.18579 | 0.327985 |  |
| **MUD1** |  |  | -0.541323 | 0.436765 | -0.205855 |  |
| **NAM8** | 0.183207 | 0.071256 | -0.625751 | 1.45417 | -2.187895 | -0.395761 |
| **YNR004W** |  |  | -0.587028 | -1.075838 | -1.184768 |  |
| **PRP11 - DAMP** | 0.737429 | 0.072385 | -0.713435 | 0.957469 | -6.814921 | -5.506216 |
| **SNU66** | 0.560014 | -0.167944 | -2.488508 | 0.728695 | -1.798826 | -1.336698 |
| **MUD2** | 0.12932 | -2.297206 | 0.079311 | -0.06729 | -5.966446 | -2.389482 |
| **TGS1** | 0.339149 | -0.994894 | -2.95903 | 0.260627 | -8.192292 | -7.29975 |
| **CBC2** | 1.532496 | -6.086238 | -2.508231 | 0.848799 | -2.333018 | -7.371856 |
| **BRR1** | -0.333035 | 1.199011 | -3.16917 | -2.023493 | 3.421831 | 3.849955 |
| **BUD13** |  |  |  | -0.516804 | -4.976274 | -2.907655 |
| **IST3** | -1.058478 | -0.20722 | -1.839788 | -1.070941 | -6.200557 | -7.454786 |
| **LEA1** |  |  | 1.808087 | -0.499393 | 0.872725 |  |
| **SNU66 - NEW** | 1.824479 | 0.774623 | 0.734044 | 2.316023 | 1.786144 | 2.124467 |
| **LIN1** | 0.481768 | -1.295096 | -0.13391 | -0.016899 | -4.893495 | -4.786913 |
| **PML1** | -1.052911 | 0.128747 | 0.455823 | 0.460355 | 0.470787 | 1.431279 |
| **PAT1** |  |  | 1.318115 | -0.153478 | 4.226803 |  |
| **LSM1** | -0.197288 | -10.671212 | 1.036188 | 0.835875 |  | 4.226803 |
| **SNT309** |  |  | 0.185271 | -0.143612 | -11.588963 |  |
| **LSM3 - DAMP** | 1.143884 | -1.483656 | -2.312203 | 0.827997 | 2.565712 | 1.986188 |
| **LSM7** | -0.253388 |  | -3.997775 | 1.915465 | -0.371439 | 2.800429 |
| **LHP1** | 0.47525 | 0.567531 | 1.691578 | 1.140973 | 1.72552 | 2.144815 |
| **LOC1** | -1.444203 | -0.030907 | 0.233812 | -0.281771 | 0.059493 | -0.395375 |
| **RNH70** |  |  | 0.626161 | -0.034931 | -0.468807 |  |
| **SXM1** | -0.509969 | -0.194449 | 0.975151 | 0.212577 | -0.032904 | -0.552076 |
| **PRP18** | 0.309025 |  | -0.96564 | 0.469297 | -0.774178 | -0.618054 |
| **MSS18** | 0.178141 | 0.458663 | 1.3027 | 0.618235 | 0.434385 | -0.514323 |
| **SKY1** | -0.673724 | -0.710885 | 1.39123 | 0.532291 | -0.05868 | 1.132754 |
| **TRM7** |  |  | 0.46345 | -0.689305 | -0.892232 |  |
| **TAN1** | 0.322495 | 1.457473 | -0.811274 | 1.207572 | -0.59604 | 0.088024 |
| **PUS4** | -0.471317 | 0.669034 | -0.315053 | 0.872993 | 0.317501 | 0.082971 |
| **SMM1** | 0.561808 | 0.706071 | -1.043432 | 1.4816 | -1.152559 | -0.488237 |
| **YGR126W** |  |  | 0.809202 | -0.356368 | 0.96042 |  |
| **DUS1** | -0.383857 | 0.754411 | 0.361046 | 0.999074 | 0.036566 | 0.285755 |
| **YPR174C** |  |  | 1.063774 | 0.050946 | 0.339422 |  |
| **EDC3** | -0.19891 | 0.61837 | -0.355315 |  | 0.835875 | -0.153478 |
| **GSP2** |  |  | 0.31809 | -0.17046 | 0.255517 |  |
| **NNT1** | 0.679901 | 0.051005 | 0.449056 | 0.237316 | -0.466261 | -0.102192 |
| **YLR287C** | 0.513572 | 0.569097 | 0.071211 | 0.558758 | 1.142682 | 0.589141 |
| **STP2** | 0.180148 | 0.319455 | -0.740231 | 0.046865 | 0.084206 | -0.310341 |
| **GCN3** | 0.40568 | 0.240935 | 1.47066 | 1.117965 | 1.045365 | 0.586559 |
| **PUF3** |  |  | 1.034226 | -0.19891 | -0.197288 |  |
| **CLU1** |  |  | -0.292568 | 0.224604 | 0.822806 |  |
| **REX3** | 1.143763 | -0.102057 | -0.262787 | 0.087924 | 1.14333 | 0.09821 |
| **GIS2** | 0.41923 | 0.585761 | 0.477739 | -1.239189 | 1.356814 | -0.572171 |
| **GFD1** |  |  | 0.395192 | -0.508471 | 1.683574 |  |
| **ZDS1** | 1.035892 | 0.413037 | 0.143507 | 0.619186 | -0.033154 | 0.622667 |
| **CUS2** | 1.145347 | -0.471771 | 0.191392 | 0.331608 | 0.105973 | -0.370594 |
| **DUS3** | -0.246885 | 0.040685 | 1.140347 | -0.461649 | 1.142104 | 0.542944 |
| **DUS4** | -0.632637 | 0.249215 | 0.565383 | -0.790589 | -1.10743 | -0.640266 |
| **REX4** | 0.588578 | 0.396375 | 2.036191 | 0.366389 | 1.071234 | 0.85567 |
| **RNY1** | -1.372279 | 0.682138 | 1.311635 | 1.146497 | 0.86111 | 0.396257 |
| **KAP114** | 0.081718 | 0.141185 | -1.948743 | 0.171942 | -0.278608 | -0.137185 |
| **SHE2** | 0.242616 | 0.748844 | -0.414933 | 0.368425 | -0.730066 | 0.630042 |
| **SBP1** | -0.168974 | 0.8477 | 0.665806 | -1.157683 | -2.664032 | -1.046507 |
| **YOR006C** |  |  | 0.715783 | 0.123601 | -0.636091 |  |
| **CKA2** | 0.5864 | 0.184338 | 1.534891 | -0.788152 | 2.048936 | 2.670316 |
| **MYO4** | 0.308124 | 0.296544 | -0.263706 | -0.529898 | 0.237148 | 0.270992 |
| **MAK32** | -0.378088 | 0.28566 | 0.618726 | -0.397434 | 2.807816 | 0.528202 |
| **UTP13 - DAMP** | 0.485821 | -0.20842 | 0.245626 | 0.288168 | 0.450479 | 0.240144 |
| **UTP21 - DAMP** | -0.275604 | -0.974152 | -0.519502 | -1.362536 | -0.52909 | 0.25084 |
| **YBL028C** | -0.222997 | 0.43045 | 1.014503 | -1.227059 | 0.73111 | 0.936038 |
| **MAK11** | 1.283194 | 0.826212 | 3.032554 | 0.133363 | 0.128671 | 0.927527 |
| **GRC3** | 0.084233 | 0.932376 | 1.016426 | -0.751577 | 0.531598 | 0.821039 |
| **URB1** | 0.946341 | 0.504067 | -0.227756 | 0.513883 | 0.140384 | 0.245004 |
| **GRS1** | -0.792444 | 0.537677 | 0.81572 | -0.078128 | -0.096873 | -0.675185 |
| **UTP4** | 0.936626 | 0.29636 | 0.537251 | 0.921997 | -3.510061 | -4.165585 |
| **RIA1 - DAMP** | 0.562747 | 0.056001 | 0.569664 | -0.108605 | 0.130619 | -0.589816 |
| **CWC25 - DAMP** | -0.14805 | 0.788805 | 0.501625 | -0.857318 | 0.693599 | -0.193245 |
| **TIF6 - DAMP** | 0.523839 | -0.520047 | 0.354826 | -0.178333 | -0.587161 | 0.024972 |
| **MAK5** | 0.813733 | 0.989645 | 0.308886 | 0.584526 | 0.85903 |  |
| **PRP3** | -1.07739 | 0.625609 | 0.311381 | 0.32548 | 0.762541 | 0.973507 |
| **SAD1** | -0.489947 | 0.298016 | 0.446359 | 0.239692 | 0.279714 | 1.271852 |
| **PRP22** | -0.222815 | 0.255583 | -0.231557 | -3.500667 | 0.074637 | 0.54295 |
| **SNM1** | -1.664762 | 1.992064 | 0.424571 | 0.535817 | -0.058692 | 0.400211 |
| **PRP31** | 0.24208 | 0.021227 | 0.326961 | 0.622786 | 0.119024 | 0.124739 |
| **SPB4** | -0.061504 | -0.073361 | 0.460149 | -0.226535 | 0.484294 | -0.319212 |
| **NUP49** | 0.438159 | -0.632486 |  | -0.289928 | 0.580539 | 0.293513 |
| **ROK1** | -0.57507 | -1.037471 |  | -0.489225 | 0.00372 | -0.399111 |
| **CFT2** | -0.566357 | -0.050451 | -0.507553 | -0.657256 | -0.020886 | 0.360131 |
| **DIP2** | -0.749182 | -1.002885 | -0.753673 | -0.564592 | 0.180465 | 0.460199 |
| **SRN2** | -0.27034 | -1.327272 | -0.779663 | -0.042352 | 0.165854 | 0.378705 |
| **SNU71** | 0.611692 |  | 0.635891 | 0.084266 | 0.020126 | 0.582688 |
| **RRP3** | 0.918151 | -0.531166 | -0.687163 | -0.62189 | -0.277791 | 0.0098 |
| **NUP82** | 0.537579 | -0.314165 | 0.994117 | -0.001868 | 0.142813 | 1.209845 |
| **PRP21** | 0.266312 | -0.041754 | 0.354159 | -1.092728 | 0.356134 | 1.197643 |
| **NAB2** | -0.90612 | -0.448579 | -0.054257 | -0.317543 | 0.445827 | 0.83208 |
| **RRP4** | -0.375503 | -1.056625 | -1.918714 | -1.223198 | -1.596323 | -1.168384 |
| **NUP159** | -0.566048 | -0.700008 | -1.249061 | -0.500151 | -0.555442 | -0.374249 |
| **SOL1** | -0.441092 | 0.204759 | -0.125969 | -0.562311 | 0.08498 | 0.052822 |
| **LSM4** | -2.888505 | -1.12194 | -0.228004 | -4.457187 | 0.436213 | 1.214827 |
| **EFT1** | -2.02735 | 0.974848 | 0.954508 | 0.014376 | 0.183094 | 0.489319 |
| **RLI1** | -1.436463 | -1.000784 | 0.774616 | -1.684859 | -0.995211 | -0.014443 |
| **PRP42 - DAMP** | -1.185338 | 0.25266 | -0.405665 | -0.148323 | 0.370046 | 0.016301 |
| **DCS1** | -0.258709 | -0.226435 | -0.028301 | -0.276308 | -0.334887 | 0.097811 |
| **YGR251W** | 0.213814 | -0.291271 | -0.383976 | 0.608329 | -1.741236 | -0.740285 |
| **NTR2** | 0.581749 | -0.31897 | 0.540517 | 0.869512 | 0.454635 | -0.112874 |
| **PXR1** | 0.827133 | -0.688975 | -0.957767 | -0.562944 | 0.321115 | -0.960316 |
| **YJU2** | 1.060489 | -0.471545 | -0.945985 | -0.072446 | -0.4706 | -1.002374 |
| **YJR141W** | 0.15605 | -0.464883 | -0.855224 | 0.129497 | -0.811044 | -1.099071 |
| **UTP9** | 0.16656 | -0.575893 | -0.488853 | -0.221713 | 0.403782 | -0.860921 |
| **UTP18** | 0.911347 | -0.099827 | -0.757106 | -0.140478 | -1.518455 | -0.16531 |
| **NOP9** | 0.770388 | -1.6963 | -0.878186 | 0.121538 | -1.741306 | -2.528605 |
| **UTP11** | 0.077652 | -0.46009 | -3.159979 | -0.137328 | -0.592646 | -1.336074 |
| **YRB2** | -0.139927 | -2.62128 | -1.152297 | -0.403447 | -1.482762 | -0.190955 |
| **YHR020W** | 1.082328 | 0.262163 | 0.066702 | 0.204501 | 0.623124 | -0.134311 |
| **IPI1** | 1.34172 | 0.681549 | 0.036762 | 1.019253 | 0.114375 | -0.075203 |
| **NUP157** |  |  | 0.879465 | 0.888981 | 0.339744 |  |
| **IMD2** | -0.970323 | 0.881239 | 0.702311 | 0.594502 | 0.473856 | 0.028643 |
| **PAN3** | 0.54363 | 0.753207 | 2.185582 | 0.380896 | 0.143354 | -0.386497 |
| **IMD4** | -1.227717 | -0.580284 | -0.100914 | 0.061353 | 0.153933 | 0.306781 |
| **DMA2** | -0.383911 | 0.254115 | 0.018895 | 0.324717 | 1.10197 | 0.623748 |
| **RPG1** | 0.414291 | 0.858737 | 0.483643 | 0.30452 | -0.818527 | -0.552871 |
| **GCD6** | 0.29343 | -0.640023 | 1.43007 | 0.516174 | -2.314229 | -2.424385 |
| **PAP2** | -0.336231 | -0.754325 | 0.668459 | -0.085176 | 0.748801 | -0.085389 |
| **TSR1** | -0.078263 | -0.016478 | -0.084074 |  | -0.980876 | 0.057253 |
| **CDC36** | 0.180591 | 0.598457 | -2.976234 | 0.924792 | -1.295527 | -0.820899 |
| **RIO2 - DAMP** | 0.078433 | -1.695468 | -0.000164 | -0.501563 | -0.755249 | -1.105896 |
| **NOC4 - DAMP** | 0.765894 | 0.434201 | 0.687911 | -0.682837 | -0.083398 | -0.881474 |
| **ABD1** | 0.168687 | 0.303311 | -0.331313 | -0.214715 | 0.789047 | 0.969911 |
| **PAB1** | -0.161386 | -1.060381 | -1.941885 | -2.808808 | 2.565614 | 3.246447 |
| **PRP6** | 0.304726 | 0.362696 | 0.231624 | 0.26555 | 0.847684 | 0.185953 |
| **PWP2** | 0.774104 | 0.417616 | 0.794582 | 1.128442 | 0.693557 |  |
| **DBP10** | 0.107757 | 0.3411 | 0.874512 | 0.619874 | 0.011949 | 0.464167 |
| **LUC7** | 0.905436 | 0.267952 | 0.529703 | 0.692986 | 0.813224 | 0.925759 |
| **SNU23** | 0.709846 | 0.392708 | 0.590675 | 0.452098 | 0.9844 | 0.628461 |
| **REX2** | 1.825998 | 1.127921 | 0.63906 | 1.677571 | 1.154896 | 0.35355 |
| **GCD14 - DAMP** | 0.370704 | -1.155862 | 0.443539 | -5.873741 |  | -3.957955 |
| **PRP19** | 0.77399 | 1.152309 | 0.070475 |  | -0.218568 | 0.297743 |
| **PRP40** | -0.188776 | 0.672019 | 0.787892 | 1.115005 | -0.732415 | -0.510606 |
| **DPS1** |  | 1.097144 | -2.021662 | 0.136851 | -4.856959 | -2.4174 |
| **CLF1** | 1.125677 | 0.595498 | 0.928656 | 1.600164 | 0.016781 | -0.13387 |
| **NOT3** | 1.805281 | 2.845927 | -3.476759 | 0.358267 | -0.97746 | -3.976729 |
| **PRP5** | 0.443869 | 0.321439 | 0.187375 | 0.000629 | 0.484217 | 0.456342 |
| **GAL3** | 0.599376 | 0.855748 | 1.812849 | 1.998922 | -0.220928 | 0.567872 |
| **GCN20** | 1.137622 | 0.007793 | 2.768463 | 1.052228 | 0.61729 | 1.537346 |
| **GCN1** | 1.034226 | -0.264768 |  | -0.355315 | 1.036188 | 1.318115 |
| **CTL1** | 0.362323 | 0.583136 | 0.762759 | 0.14534 | 1.454234 | 0.183102 |
| **NGL2** | 1.673567 | 0.81347 | 0.74379 | 1.036595 | 1.778148 | 0.92422 |
| **CAF120** | 0.761977 | 0.433368 | 0.541696 | 1.662596 | 0.949863 | 1.458795 |
| **CAF40** | -0.630155 | 0.053873 | -0.32992 | -0.17247 | 0.558013 | -1.153772 |
| **RPA49** | 0.065635 | -1.036534 | -0.152555 | -0.501248 | 0.983659 | 0.739868 |
| **SYC1** | 0.238627 | -0.837567 | 0.345432 | 0.557563 | -0.301941 | 0.936238 |
| **DBP1** | 0.281969 | -0.475962 | 0.01694 | 0.387609 | 0.474798 | 0.149881 |
| **SCD6** | 0.119237 | -0.274256 | 0.773757 | -8.921277 | 0.325234 | -1.394574 |
| **HHO1** | -0.508455 | -1.309438 | -0.525691 | 0.98261 | 0.048674 | 0.052483 |
| **RPA14** | -1.546571 | -3.703826 | -3.125282 | -0.129316 | -2.204074 | -5.672925 |
| **RPA34** | -2.494099 | -8.95169 | -6.635742 | -0.029883 |  | -5.106008 |
| **TRM8** | 0.368155 | 0.508986 | 0.488981 | 0.606935 | 0.526253 | 1.616196 |
| **UGA4** | 0.051655 | 0.953016 | -0.340443 | 0.366301 | 0.56838 | 0.648343 |
| **SEN34** | -0.924678 | 0.016304 | -1.021312 | -0.027601 | 0.039352 | 0.301934 |
| **PRP9** | 0.542659 | 0.228355 | -0.328396 | 1.013106 | -0.345661 | -0.142467 |
| **RRP42** | 0.526616 | -0.120433 | 0.371021 | 0.359765 | -0.447486 | 0.223314 |
| **GLE1** | 0.349914 | 0.28862 | -0.986013 | 0.069869 | 0.398988 | -0.218533 |
| **SAS10** | 0.179006 | -0.129271 | 0.075914 | 0.090076 | 0.141394 | 0.072147 |
| **FAL1** | 0.199184 | 0.361847 | -0.860036 | -0.81023 | 0.16541 | 0.706142 |
| **RSE1** | -0.399854 | 0.147558 | -0.211426 | -0.001208 | 0.420325 | 0.315152 |
| **RNT1** | -0.633981 | -0.81976 | -0.812401 | 0.236256 | 0.114963 | -0.169928 |
| **CFT1** | -0.135993 | -0.056525 | 0.094022 | -0.174751 | 0.139567 | 0.122771 |
| **ENP1** | -0.189545 | -0.674203 | -1.247999 | -0.33435 | -0.591899 | -0.535392 |
| **DBR1** | -0.188452 | 0.155669 | 0.01269 | -0.185781 | -0.186411 | -0.551296 |
| **ECM32** | -0.401709 | -0.167363 | -1.148334 | -0.052557 | -1.480414 | -0.854297 |
| **CWC27** | -1.51949 | -1.595474 | -1.387101 | -1.074595 | -1.331082 | 0.87873 |
| **CWC22** | -0.496601 | -0.385014 | -0.883281 | -0.523296 | 0.054974 | -1.625795 |
| **SHQ1** | -0.202503 | -0.61712 | -1.146971 | -0.68244 | 0.170507 | -0.596821 |
| **DHR2** | 0.057128 | -1.824366 | -0.988281 | -0.342914 | 0.038228 | -1.768119 |
| **RIX1** | 0.221878 | -1.695479 | -2.331292 | -0.587857 | -0.830985 | -4.165675 |
| **RPC17** | -0.220515 | -1.462303 | -1.161628 | -1.216731 | -0.456339 | -0.041535 |
| **FAF1** | -0.157447 | -0.976671 | -0.588467 | -0.721929 | -0.405231 | -0.890741 |
| **TRZ1** | -0.83882 | -0.533282 | -1.40424 | -0.957174 | -0.205414 | -1.060733 |
| **BDP1 - DAMP** | -1.998162 | -0.144825 | -0.08138 | -0.838314 | -0.271471 | -0.190076 |
| **SEN2** | -0.375944 | -0.323422 | -0.20977 | -0.295085 | -0.633901 | -0.960616 |
| **SEN15 - DAMP** | -0.590733 | -0.998922 | 0.336395 | 0.139693 | -0.772935 | -1.036345 |
| **CUS1 - DAMP** | 0.213802 | 0.303583 | 0.545615 | 0.20631 | -0.0277 | -1.286935 |
| **SMD2 - DAMP** | 0.345271 | -0.865259 | -0.705177 | -0.380297 | -0.287638 | -1.825083 |
| **NUP116 - DAMP** | 0.4571 | -1.47625 | -0.771315 | -0.943573 | -0.940509 | -2.636088 |
| **CEF1 - DAMP** | 0.207107 | -2.738013 | 0.511579 | -0.676744 | -0.533452 | -1.394734 |
| **ECM16 - DAMP** | 0.341204 | -1.517389 | 0.879859 | 0.22856 | -1.453358 | -1.84675 |
| **PWP1 - DAMP** | -0.748372 | -0.585694 | 0.694283 | 0.501406 | -0.397241 | -0.627454 |
| **PRP24 - DAMP** | -0.063321 | -1.739408 | -0.337032 | 0.742309 | -0.618882 | -1.595016 |
| **SEN1 - DAMP** | 0.381599 | 0.32075 | 0.453655 | 0.695532 | 0.119012 | -1.052366 |
| **KAP95 - DAMP** | 1.270449 | -0.172079 | 0.241453 | -0.949809 | -0.203352 | -1.095315 |
| **RRP5 - DAMP** | 0.137245 | -0.151521 | 0.145455 | 0.519744 | -1.932585 | -2.520351 |
| **DBP9 - DAMP** | 0.318893 | -0.912236 | -0.675191 | 0.010173 | -1.175924 | -1.290667 |
| **IFH1 - DAMP** | 1.176147 | -0.176205 | 0.730972 | 0.827701 | 0.184131 | -0.461304 |
| **NOP14** | 0.383357 | 0.283116 | -0.218809 | -0.686162 | -0.946672 | -1.444164 |
| **MAK21** | -0.083973 | 0.967781 | 1.127234 | -0.090783 | -0.138862 | -0.735698 |
| **FHL1 - DAMP** | -0.229235 | -0.093853 | 0.499975 | -0.868876 | -0.112994 | -1.734748 |
| **DBP2 - DAMP** | 1.636722 | 0.481124 | 0.94851 | 0.453624 | -0.596379 | -0.932132 |
| **NOP8 - DAMP** | -0.396005 | 0.351829 | -0.508874 | 1.08576 | -1.101696 | -0.565705 |
| **AIR2** | 0.534138 | 1.18024 | 0.341297 | 0.48537 | 1.294855 | 0.744526 |
| **HRB1** | 1.329192 | -0.441867 | 0.047952 | 0.323847 | -0.012743 | -0.583632 |
| **TRM1** | -0.508628 | -0.441774 | 1.8711 | -0.143983 | -0.641401 | -0.615188 |
| **TRM1 - NEW** | 2.333467 | 1.226237 | 2.705756 | 2.648666 | 3.900072 | 3.89922 |
| **SNU114** | 0.151149 | 0.761149 | 0.063582 | -1.076998 | 0.283198 | 1.06332 |
| **TRM82** | -1.087069 | 0.930054 | -0.579737 | 0.76686 | 0.012079 | -0.157103 |
| **TAD1** | 0.375518 | 0.701275 | -0.190964 | 0.522111 | -0.155203 | 0.288198 |
| **SOL3** | -0.280173 | 0.888905 | 0.610085 | 0.559907 | 0.900257 | 0.676363 |
| **ANB1** | -0.281627 | 0.642082 | 0.000972 | 0.599101 | -1.003905 | 0.13523 |
| **TEF4** | -0.396685 | -0.006808 | -1.503648 | 0.68523 | -1.348798 | -0.02678 |
| **TRM2** | -0.80366 | 0.264111 | -2.091517 | -0.13058 | 1.010132 | -0.234737 |
| **SSF2** | -0.039468 | -0.703055 | 0.535335 | -0.158528 | 0.453111 | 0.507468 |
| **GRS2** | 0.309781 | -0.055374 | -0.502147 | 0.147795 | -0.406104 | -0.648083 |
| **TIF4632** | -0.00659 | -1.950532 | 0.410782 | 0.657959 | 0.261222 | 0.193638 |
| **YBL104C** |  |  | 1.040852 | -0.505713 | 0.022703 |  |
| **MOT2** |  |  | 0.157193 | -0.056673 | 1.194129 |  |
| **RNH201** | 0.835095 | 0.456527 | -0.114963 | -0.124042 | 0.97654 | -0.098083 |
| **MSN5** |  |  | -1.532847 | -0.809979 | 0.908355 | -1.000752 |
| **YBR094W** |  |  | -1.143223 | -1.593284 | 0.061945 | 0.569107 |
| **PML39** |  |  |  | -0.258802 | -0.994379 |  |
| **SKI2 - NEW** | 1.008835 | -0.408745 | -1.046017 | 0.306376 | -2.32636 | -4.788298 |
| **SHE3** |  |  | 0.518314 | 0.81129 | -0.533814 |  |
| **YDL089W** |  |  | 0.185138 | 0.174153 | 0.193032 |  |
| **YDR458C** |  |  | 0.25195 | 0.446649 | -0.733629 |  |
| **MLP1** | 1.595731 | 0.113948 | -0.027901 | -0.186938 | -2.196815 | -0.444923 |
| **DTD1** | 0.07411 | 0.795238 | 0.153476 | 0.71718 | -0.027684 | 0.213416 |
| **WHI4** | 0.960011 | 0.25112 | -0.569025 | 0.937054 | 0.810561 | 0.95402 |
| **SNL1** |  |  | 0.316394 | -0.086919 | 0.009079 |  |
| **UBS1** | 0.475404 | 0.520793 | -0.508875 | 0.259533 | -0.657665 | 0.669197 |
| **DDI1** |  |  | -0.523618 | 0.762734 | -0.448013 |  |
| **SLH1** | -0.626758 | -0.093784 | -1.713669 | -1.201671 | 1.955852 | 1.262393 |
| **CAF4** | 0.083655 | 0.073749 | 0.458726 | -0.085118 | 1.36622 | 0.729271 |
| **EAP1** |  |  | 1.145427 | -0.194093 | 0.064031 |  |
| **SSM4** | -0.403604 | 0.057905 | -1.299989 | -0.707573 | 0.015501 | -0.874256 |
| **STP1** | 0.041289 | 0.431983 | -5.427363 | 0.714515 | -0.1701 | 0.514536 |
| **MRM2** | -0.488389 | 0.060366 | -1.050714 | 0.771682 | 1.691145 | 1.624541 |
| **ALB1** |  |  | -1.111917 | 0.394073 |  |  |
| **YNR024W** |  |  | 0.018119 | -0.247616 | -0.057076 |  |
| **SPB1** | 0.244315 | -0.721928 | -0.753597 | -0.865118 | -0.172548 | 0.527361 |
| **YCR087C-A** | -0.221717 | -0.23033 | -1.563999 | 0.540037 | 0.443105 |  |
| **STP4** | 1.008099 | -0.087717 | -0.244467 | 0.037408 | -0.426664 | 0.916308 |
| **SOL2** | -0.195426 | 0.130985 | 0.953198 | -0.095608 | -0.718488 |  |
| **RAD51** |  |  | 0.389609 | 0.832353 | 0.670602 | 0.366765 |
| **RTT101** | -2.206075 | 0.354818 | 0.236135 | -0.656301 | -1.756694 | -1.944582 |
| **RAD52** | -2.127836 | -0.199564 | -1.180028 | 0.266119 | -1.708411 | -2.160132 |
| **CKB1** |  |  | -5.413928 | 0.316197 | 1.279008 | -0.047223 |
| **CKB2** |  |  | -7.262539 | -1.015786 | -1.252799 | -2.103584 |
| **WHI3** | 1.024323 | 0.060236 | -2.304044 | 0.348586 | 0.320266 | 0.466281 |
| **CKA1** |  |  | 1.104598 | 0.460246 | -0.015302 | -0.471832 |
| **CAF130** | -0.192622 | 0.347489 | -2.182148 | -0.138453 | 0.790145 | 1.142374 |
| **YDR026C** | -1.535234 | 0.140443 | -0.365197 | -1.892242 | -0.963169 | -0.084265 |
| **PES4** | 0.756787 | -0.053861 | -0.510081 | -0.466506 | 0.138425 | -0.323878 |
| **IMD3** |  |  | 0.035275 | 0.752954 | 0.634126 |  |
| **EDC1** |  |  | -0.607176 | -0.434719 | 1.220052 |  |
| **YGR283C** |  |  | -1.66598 | -0.503287 | 0.558632 |  |
| **NOP13** | -0.111526 | 0.513779 | 0.877001 | 0.92901 | 0.241354 | -1.088496 |
| **FIR1** |  |  | 0.211793 | -0.571991 | 0.984876 |  |
| **NMD4** |  |  | 0.150592 | 0.525863 | 0.450879 |  |
| **PUS5** | 0.814177 | -0.318032 | 0.440053 | -0.573182 | 1.660916 | 1.62063 |
| **YRA2** | 0.56065 | -0.119115 | 1.038791 | -0.099978 | 1.528651 | 0.502117 |
| **EDC2** |  |  | 1.714585 | -0.109718 | 1.261393 |  |
| **PRP12** | 1.21305 | -0.044632 | 1.289 | 0.757897 | 0.485212 | 0.232299 |
| **STP3** | -0.073822 | 0.077003 | 0.875536 | 1.400187 | 0.239159 | 0.657814 |
| **YPL009C** |  |  | 0.941552 | 0.057384 | -0.614688 |  |
| **TIS11** | -0.591776 | -0.336938 | 0.509618 | -0.266489 | 0.362552 | 0.377235 |
| **PUS2** |  |  | 0.498058 | -0.17851 | -0.348352 |  |
| **MIP6** | 0.098208 | 0.269829 | 1.356846 | 0.134966 | 0.135943 | 0.564825 |
| **PUS6** | 0.414239 | 0.414829 | 0.231581 | 1.375689 | 1.627869 | 0.034812 |
| **RBG2** | 0.771486 | 1.130861 | 0.817485 | 0.133602 | 0.568347 | 0.709671 |
| **RIT1** | 1.44221 | 0.672679 | 3.936149 | 1.26673 | 1.479921 | 0.591626 |
| **JSN1** | 0.021618 | -0.34761 | 1.169654 | 0.403892 | 0.251434 | 1.027044 |
| **NUP100** | -0.352569 | -0.142065 | 1.324963 | 0.016476 | -0.239074 | -0.926803 |
| **LOS1** | 0.233007 | 0.562879 | 2.484513 | 0.012443 | -2.217068 | -0.411934 |
| **HEF3** | 1.521189 | 0.397927 | -0.490324 | 1.79142 | -0.073321 | 0.189563 |
| **MOD5** | 0.460212 | 1.043287 | -1.341868 | 0.536423 | -0.212417 | -0.777689 |
| **CAF20** | 2.122855 | 0.588209 | 0.549324 | 1.433893 | 1.169283 | 1.363676 |
| **PUS7** | 0.581548 | 0.30426 | -2.505231 | 1.102398 | -1.394599 | -1.995962 |
| **PUS1** | -0.384865 | 0.002455 | -5.516834 | 0.404183 | -0.059965 | -0.050751 |
| **USA1** | -0.270571 | 0.409699 | -0.565622 | -0.951155 | 2.089746 | -0.022872 |
| **CNM67** | 0.372415 | 0.736318 | -0.089516 | 0.380134 | 0.417816 | 0.427292 |
| **PUF2** | 0.637805 | -0.114716 | 0.326456 | 1.071641 | 0.943574 | -0.11311 |
| **DRS2** | 0.70257 | 0.291762 | 0.541165 | 1.342287 | -0.08363 | 1.25268 |
| **TRM11** | 0.240885 | -0.230744 | 0.340393 | 0.012433 | 2.56983 | 1.613571 |
| **YBR141C** | 1.253567 | -0.01779 | 0.622599 | 0.784795 | 1.999584 | 2.219807 |
| **YKR096W** | -0.307232 | 0.419193 | -0.965108 | 1.005908 | 1.964189 | 0.956471 |
| **TRM3** | 2.666753 | 0.682563 | -0.885098 | 2.397239 | 1.272565 | 1.891278 |
| **RNP1** | 0.318124 | 0.37332 | 0.278932 | 0.153486 | 0.540421 | 0.804089 |
| **ITT1** | -0.211457 | 0.171103 | -0.190014 | -0.003558 | 1.302722 | 1.714225 |
| **GIR2** | 0.346112 | 0.26054 | -0.487689 | 0.968281 | 1.195517 | 0.846639 |
| **YHR087W** | 0.682156 | 0.523055 | 0.667116 | 1.187041 | -0.128594 | 1.184145 |
| **YGR250C** | 0.475863 | -0.054513 | -0.188033 | 0.629891 | 2.654556 | 2.28893 |
| **SRO9** | 1.363175 | -0.193548 | -0.842973 | 0.641913 | 2.32433 | 4.126431 |
| **FMT1** | 0.86578 | 0.420651 | 1.226091 | -1.020296 | 0.903961 | 1.875851 |
| **NCL1** | 0.130817 | 1.557893 | 2.834318 | 0.043568 | 0.806767 | 1.893793 |
| **PTH2** | -0.281802 | 0.343974 | -0.15341 | 0.474304 | 1.879659 | 0.7949 |
| **POA1** | -0.085409 | -0.25534 | -0.710851 | 0.589838 | 0.79965 | 0.630196 |
| **YIL096C** | 0.852225 | 0.246642 | -0.752795 | 0.248827 | 1.819814 | 0.29507 |
| **YNL022C** | -0.643799 | 0.379985 | 0.19451 | 0.167878 | 0.368406 | 1.770459 |
| **YMR087W** | 0.814697 | -0.001541 | -0.5229 | 0.734368 | 1.037096 | 0.629559 |
| **YDR198C** | 0.505184 | -0.166313 | 1.013227 | 0.583341 | 1.288635 | 1.450458 |
| **LOT5** | 0.858426 | -0.306879 | 1.509375 | 0.597645 | 1.580323 | 1.293982 |
| **YGR093W** | 1.367239 | 0.404092 | -0.421772 | 0.740826 | 0.548805 | 1.144517 |
| **CWC15** |  |  | -0.814902 | -0.018087 | -0.159882 |  |
| **RNA14** | 0.236705 | -3.422406 | 0.540009 | -0.041627 | 1.032033 | 1.183606 |
| **POP1** | 0.694269 | 0.635646 | -0.385616 | -0.120642 | 1.27958 | 0.830223 |
| **SYF1** | 0.129496 | -0.201865 | -0.750318 | -0.465433 | -0.189445 | -0.353259 |
| **DRS1** |  | 0.471734 | -0.571402 | -0.812312 | 0.994336 | 0.00039 |
| **MSL5** | -0.354357 | 0.86754 | 0.875012 | 0.248901 | 0.258716 | -0.018468 |
| **DBP6 - DAMP** | -0.4542 | 0.293874 | 0.84655 | -0.697976 | 0.287532 | -0.019902 |
| **DCP2 - DAMP** | -0.680006 | 0.901016 | 1.518252 | 0.648974 | 1.082691 | -0.235299 |
| **CSL4 - DAMP** | -0.398132 | -0.256522 | -0.11915 | 0.613016 | 1.457554 | 0.185072 |
| **SUI3 - DAMP** | -0.824188 | 0.327687 | 0.427107 | -0.446621 | 0.851627 | 0.434808 |
| **TAD2** | -0.757729 | 0.270053 | 0.375326 | 0.320543 | 0.576339 | 0.545335 |
| **RPO21** | 1.287299 | 3.01651 |  | -0.006634 |  | 0.866372 |
| **NMD5** | -0.56741 | 2.740937 | -2.140341 | 1.377298 | 2.996838 | 1.858756 |
| **PRE9** |  |  | -12.166564 | 0.096171 | 0.883198 | -0.228441 |
| **NAM2** | -0.475895 | -0.493009 | -4.288756 | 0.353702 | -0.253972 | 0.934473 |
| **SUI2 - DAMP** | 2.951837 | 1.299271 | 1.986602 | 1.058679 | 1.995611 | 1.99346 |
| **KAP120** | 0.067906 | 1.875749 | 2.306636 | -0.202151 | 1.677746 | 1.055336 |
| **LTV1 - NEW** | -0.812766 |  |  | -0.210344 | 2.106987 | 4.719839 |
| **SLU7** | 1.108467 | 1.593781 | 1.35957 | 0.97454 | 1.030535 | 1.430181 |
| **TRL1** | 0.190671 | 0.938384 | 0.929108 | 0.398429 | 1.352939 | 0.82631 |
| **CCA1** | 0.300006 | -0.277531 | -0.373781 | -0.08789 | 0.369256 | 0.053719 |
| **BRR2** | 0.028979 | 0.260529 | -0.088005 | 0.122361 | 0.462866 | 0.268965 |
| **SQT1** | -0.273742 | 0.492048 | 0.769451 | 0.227453 | 0.75596 | 0.642745 |
| **YTH1 - DAMP** | 0.574239 | -0.045523 | 0.391688 | 0.525365 | 0.586995 | 0.749729 |
| **EBP2** | 0.515702 | 0.542139 | 0.554319 | 0.460753 | -0.057335 | -0.400615 |
| **NUP192** | 0.698439 | 0.928273 | 0.580548 | 0.596358 | -0.406511 | -0.527063 |
| **SKI6** | -0.272195 | 1.002589 | 0.62355 | -0.459762 | -1.553674 | -5.805027 |
| **YEF3 - DAMP** | 0.299224 | 0.424744 | 0.544657 | -0.289443 | -4.374715 | -4.887391 |
| **CAF16** | 0.88957 | 0.711071 | 1.34674 | 0.775227 | 1.126203 | 0.053685 |
| **RNA15** | 0.826837 |  | -0.057183 | 0.339514 | 1.113164 | 0.944393 |
| **GCD10** | 0.193765 | 0.924063 | -0.092476 | 0.27002 | 0.383051 | 0.634394 |
| **RRP9 - DAMP** | 0.352254 | 0.952027 | 0.486678 | 1.038066 | 0.775206 | 0.818857 |
| **POP3 - DAMP** | 0.302894 | 0.821927 | 0.172189 | 0.433715 | 0.791804 | 0.910231 |
| **MRD1 - DAMP** | 0.154766 | 1.024396 | -0.374468 | 0.113815 | 1.142037 | 1.360929 |
| **DIS3 - DAMP** | 1.016511 | 0.560936 | 0.343552 | 1.208919 | 1.261611 | 0.756625 |
| **HRP1** | 1.184203 | 0.369013 | 0.029859 | 0.361252 | -0.041579 | 0.361736 |
| **ACC1** | 0.380655 | 0.380448 | 0.418036 | 0.760625 | 0.700863 | 0.281661 |
| **IMD1** | -0.744945 | -0.095862 | 0.349104 | -0.348195 | 1.073798 | 0.137564 |
| **ELP2** | 0.73785 | -1.702301 | -0.740406 | 0.415009 | 0.276842 | 0.125418 |
| **ELP6** | -0.854527 | 0.152727 | -3.241594 | 0.573098 | 0.215296 | 0.009983 |
| **ELP3** | -1.038533 | -0.89133 | -1.770148 | -0.985222 | 0.400647 | 0.69422 |
| **ELP4** | -1.157237 | -1.443315 | -0.6248 | -0.160293 | 0.608623 | 2.141906 |
| **IKI1** | -0.404827 | -0.261654 | -2.076202 | 0.808328 | 1.410913 | 1.22547 |
| **IKI3** | -0.984707 | -0.332277 | -0.940463 | -0.364943 | 0.852893 | 0.326983 |
| **TRM10** | -0.11418 | 0.006908 | -0.617689 | 0.075912 | -1.238269 | -0.062416 |
| **IKI3 - NEW** | 0.258834 | -0.733062 | 0.834397 | -1.902257 | 0.458458 | 1.274582 |
| **POP8 - DAMP** | 0.705169 | -6.916415 | 2.483404 | 1.045006 | -8.944744 | -11.131738 |
| **POP8 - NEW** | 0.085909 | -1.317075 | 1.03989 | 0.422697 |  | -1.98342 |
| **POP7 - DAMP** | 1.031208 | -2.106181 | 2.216051 | -0.836414 | 0.446613 | 1.020561 |
| **GCD11** | -1.322397 | -0.905332 | 1.44784 | -3.669471 | -1.657442 | -1.050586 |
| **LCP5 - DAMP** | -0.006376 | -0.226453 | -4.35669 | -4.794821 | -5.369609 | -2.834694 |
| **DED81** | -2.22224 | -0.155672 | -2.127936 | -7.198128 | -6.252619 | -2.703277 |
| **POP6 - DAMP** | 0.241935 | -2.883391 | 0.238851 | -0.006152 | -0.900783 | -1.288971 |
| **RRP7** | -0.056893 | -0.831402 | -1.552196 | 0.02028 | -0.382607 | 0.20971 |
| **NOP1 - DAMP** | 1.111569 | -4.588007 | -1.855086 | -0.764498 | -8.277746 | -3.433946 |
| **SIK1 - DAMP** | 0.600811 | -5.684044 | -2.308398 | -1.509375 | -9.06772 | -9.079261 |
| **KRR1** | 0.752434 | -2.502273 | -1.70196 | -0.83197 | 1.21021 | 1.61204 |
| **SUP35 - DAMP** | -0.087487 | -0.475196 | 1.190256 | -0.165042 | -1.25685 | -2.492372 |
| **LRS4** | -2.126789 | -7.196944 | -0.514463 | -0.230939 | -14.521589 | -11.411203 |
| **NOP15 - DAMP** | -0.100785 |  | 2.860014 | -1.583909 | -0.327817 | -0.027721 |
| **BRX1 - DAMP** | -0.25938 | 0.499991 | 1.982684 | -0.076119 | -0.700721 | -1.309804 |
| **NOP6** | 1.187142 | 0.569242 | -1.994361 | 0.582144 | 1.698292 | 0.565594 |
| **UTP30** | 0.203914 | -1.244491 | -2.815129 | 0.360052 | 1.1467 | 0.963923 |
| **YLR003C** |  |  |  | 0.74063 | -0.451461 |  |
| **TIF3** |  |  | -0.226031 | -0.1934 | -0.655829 |  |
| **RPS8A** | 1.597863 | 2.147639 | 0.577525 | 0.679809 | -0.010391 | 1.744443 |
| **LTV1** | -0.868631 | -0.555804 | 1.001428 | 1.550922 | -0.964964 | 4.263027 |
| **HCR1** | -0.646266 | -0.04188 | 0.009146 | -2.921653 | 2.027809 | 3.048203 |
| **FPR1** | -0.160841 | 0.651638 | -3.318178 | -0.512359 | -0.707821 | 0.882383 |
| **TSR2** |  |  | -1.048887 | 0.496539 | -3.868094 |  |
| **PUF6** |  |  | 1.952601 | -0.386433 | 0.36688 |  |
| **CGR1** |  |  | 1.693326 | 0.475022 | -4.256277 |  |
| **TIF4631** |  |  | 1.540205 | -0.045636 | 0.246095 |  |
| **ASC1** |  |  | 1.536836 | -0.340932 | -4.574991 |  |
| **STO1** |  |  | -3.229778 | 0.487007 |  |  |
| **YCR016W** | -0.23191 | -0.154304 | 0.801545 | 0.128312 | 1.846459 | 1.448335 |
| **RSA3** | 0.446932 | 0.132643 | 2.739939 | -0.619914 | 1.054309 | 3.409763 |
| **RRP8** | 0.340892 |  | 0.007029 | 0.71939 | 1.546447 | 0.65453 |
| **MRT4** |  |  | 3.126721 | 0.067055 | -1.252863 |  |
| **BUD21** | 0.013625 | -0.174324 | 0.051218 | -0.302609 | 1.208999 | 0.800051 |
| **UAF30** |  |  | 1.706894 | -0.199057 | -0.809381 |  |
| **LRP1** | 0.819985 | -0.498387 | -1.188997 | -1.151064 | 1.328193 | 0.351131 |
| **RRP6** |  |  | -3.031556 | -0.051295 | 2.883774 | -1.596903 |
| **NOP16** | 0.803419 | 0.518595 | 1.111264 |  | 0.31845 | 0.418128 |
| **NOP12** | 0.315742 | 0.369361 | 2.567009 | -1.378109 | 2.041455 | 1.132434 |
| **SSF1** |  |  | 2.417516 | 0.196547 | 2.255822 | 4.854772 |
| **PIH1** | 0.225553 | 0.523933 | 0.036743 | 0.565751 | 2.917414 | 1.481667 |
| **MRH4** |  |  | 1.269417 | -0.372381 | 0.823997 |  |
| **DBP3** | 0.747521 | -0.288332 | 1.427326 | -0.452704 | 0.377375 | 0.647858 |
| **SRP40** | 0.747815 | 0.948663 | 0.273606 | -0.744815 | 1.887626 | -0.734 |
| **YMR269W** |  |  | 0.29743 | 0.373017 | 1.216683 |  |
| **AIR1** | 0.318147 | -0.081909 | 0.322835 | -0.048522 | 0.733449 | 0.451577 |
| **MSM1** |  |  | -8.905072 | 0.629015 | 0.428655 |  |
| **MSK1** |  |  | -8.741377 | -0.964688 | 0.905899 |  |
| **SUV3** |  |  | -6.538657 | -0.621033 | -0.193171 |  |
| **MSF1** |  |  | -10.641745 | 0.346789 | 0.391601 |  |
| **MSU1** |  |  | -5.798475 | -0.660813 | 2.840111 |  |
| **CBT1** | 0.13033 | -0.30929 | 1.589834 | -0.304478 | 0.555208 | 1.194857 |
| **TUF1** |  |  | -4.276501 | 0.567545 | -0.292623 |  |
| **MRM1** |  |  | -4.261516 | -0.139746 | -1.159757 |  |
| **MSD1** |  |  | -6.244587 | 0.473273 | 1.838443 |  |
| **SLS1** | 0.222252 | 1.089373 | -3.85819 | 0.429569 | 1.154843 | 0.940155 |
| **PET309** |  |  | -2.114186 | 0.707629 | 1.366222 |  |
| **AEP3** | 0.584095 | 0.324184 | 0.444305 | 0.823854 | -0.94487 | 0.511786 |
| **IFM1** | -0.597494 | -0.654155 | -0.731008 | 0.83377 | -0.166724 | 0.261604 |
| **CAM1** | 1.07212 | 0.889727 | 0.174958 | -1.078488 | 0.605201 | 1.281498 |
| **KEM1** | 0.808819 | 1.089209 |  | 0.536634 | 2.534738 | 0.793799 |
| **PUB1** | 0.142893 | -0.398345 | -1.497265 | -0.536247 | -2.26633 | -1.404756 |
| **YGR054W** | 0.535544 | -0.49605 | 0.345559 | -0.592169 | 0.649982 | 0.374865 |
| **TIF2** | -0.633245 | -0.014702 | -0.331916 | -1.027731 |  | 1.069206 |
| **TIF1** | -0.942674 | 2.737455 | 1.183541 | 1.075521 | 3.736012 | 2.579352 |
| **UPF3** | 0.031873 | 0.09148 | 1.372469 | -0.471275 | -2.780583 | -0.991724 |
| **NAM7** | 0.079798 | -0.109527 | 2.843151 | -0.737683 | -1.882223 | 0.154845 |
| **NMD2** | -0.262482 | 0.679505 | 1.40949 | 0.622414 | -0.608035 | -1.47561 |
| **RMP1** | -0.236384 | -0.158779 | -0.203473 | -0.527433 | -0.462753 | 0.015225 |
| **PBP1** | 0.092819 | -0.143432 | -1.160262 | 0.73269 | -4.170074 | -0.732481 |
| **LSM12** | 0.689023 | 0.281658 | -0.873244 | 0.982556 | -0.55562 | -0.871687 |
| **UBI4** |  |  | -0.686454 | 0.58725 | -1.40878 | -0.320033 |
| **TAH1** |  |  | 0.385903 | -0.29013 | -0.332702 |  |
| **SAP190** | 0.233849 | -0.750147 | -0.72873 | -0.813136 | 0.961777 | 0.216216 |
| **RNH1** | 0.358938 | -0.13916 | 0.018558 | 0.20049 | -0.53823 | -0.090422 |
| **CBP2** | 0.150327 | 0.467395 | 0.13996 | 1.026577 | 0.319812 | 0.811168 |
| **SGN1** | -0.576563 | 0.35617 | 0.586627 | 0.500699 | 0.47729 | -0.739191 |
