# Supplementary material for: Learning a Prior on Regulatory Potential from eQTL Data
Source: PLoS Genet. 2009 Jan 30;5(1):e1000358. doi: 10.1371/journal.pgen.1000358 (PMC2627940; doi:10.1371/journal.pgen.1000358)
Supplement: Table S15 — Highly correlated expression profiles in the Rosetta yeast deletion compendium. We show the significance of the correlation between the genomic expression levels of puf3Δ and gcn20Δ mutants (Pearson's correlation coefficient = 0.65; Figure S12) by comparing it with those of the pairs of arrays from Rosetta deletion mutant dataset [21]. For every pair from 300 arrays consisting of diverse mutations and chemical treatment in S. cerevisiae, we calculated Pearson's correlation coefficients, and present the pairs whose correlation coefficients are higher than 0.65. (0.05 MB DOC) [file pgen.1000358.s028.doc]

| **MUTANT1** | **MUTANT2** | **PEARSON’S CORRELATION** |
| --- | --- | --- |
| **AEP2** | **YMR293C** | 0.863 |
| **ERG28** | **AEP2** | 0.821 |
| **YMR293C** | **MSU1** | 0.793 |
| **ERG28** | **YMR293C** | 0.792 |
| **AEP2** | **MSU1** | 0.789 |
| **ERG28** | **MSU1** | 0.783 |
| **ERG2** | **ERG11 (TET PROMOTER)** | 0.773 |
| **ERG11 (TET PROMOTER)** | **ITRACONAZOLE** | 0.772 |
| **ERG28** | **TUNICAMYCIN** | 0.764 |
| **YMR014W** | **YOR078W** | 0.762 |
| **ERG28 (HAPLOID)** | **ERG2** | 0.756 |
| **FKS1 (HAPLOID)** | **FKS1 (TET PROMOTER)** | 0.744 |
| **ASE1 (**12)** | **YMR031W-A** | 0.74 |
| **ERG28 (HAPLOID)** | **ERG11 (TET PROMOTER)** | 0.735 |
| **ERG2** | **ITRACONAZOLE** | 0.731 |
| **MSU1** | **RML2 (**13)** | 0.727 |
| **ERG28 (HAPLOID)** | **ITRACONAZOLE** | 0.716 |
| **ERG28 (HAPLOID)** | **ERG3 (HAPLOID)** | 0.715 |
| **ERG2** | **ERG3 (HAPLOID)** | 0.711 |
| **VMA8** | **CUP5** | 0.702 |
| **ERG28** | **ITRACONAZOLE** | 0.688 |
| **CDC42 (TET PROMOTER)** | **KAR2 (TET PROMOTER)** | 0.679 |
| **2-DEOXY-D-GLUCOSE** | **GLUCOSAMINE** | 0.676 |
| **ERG2** | **TUNICAMYCIN** | 0.67 |
| **ERG3 (HAPLOID)** | **ITRACONAZOLE** | 0.665 |
| **ERG28** | **RML2 (**13)** | 0.657 |
| **ERG28** | **ERG2** | 0.657 |
| **ERG3 (HAPLOID)** | **ERG11 (TET PROMOTER)C-5** | 0.656 |
