# Supplementary material for: Learning a Prior on Regulatory Potential from eQTL Data
Source: PLoS Genet. 2009 Jan 30;5(1):e1000358. doi: 10.1371/journal.pgen.1000358 (PMC2627940; doi:10.1371/journal.pgen.1000358)
Supplement: Text S1 — Supplementary Methods. (0.08 MB DOC) [file pgen.1000358.s029.doc]

# Supporting Information

**Tiling array analysis.** The *mkt1-by* gene expression levels were measured using a custom tiling array designed by modifying of the sequences on the stock yeast 244k tiling array (Agilent-014741Yeast Oligo Microarray G4491A), which contains ~85% of the non-repetitive portion of the yeast genome (~12 MB) at an average probe spatial resolution of ~50 nucleotides (Agilent Technologies). Because all sequences on that probe correspond to the same DNA strand, we designed a custom array (Agilent-016248 Custom Array G4496A) in which every other probe (sequentially along each chromosome) was the reverse complement of the original sequence. Sample preparation, cDNA labeling, hybridization, washing, scanning, and image analysis were as described above. Following MASLINER adjustment, we calculated the log_2_ ratio of intensities of the signal and applied the global normalization and the intensity dependent normalization by using LOWESS as we did for the standard microarray data set (above). Then, we mapped the probes on our tiling arrays to the transcripts identified by David et al. [1] and computed the expression of each transcript as the average of the probes assigned to it.

# Supplementary Note: Puf3 Localization. Our results differ from those of other studies conducted in different strain backgrounds or under different environmental conditions. Experiments published by Sheth and Parker [2], which showed only diffuse cytoplasmic staining of Puf3-GFP, were carried out in a strain background [3] that differs from the BY background in aspects relevant to P-body composition and formation (R. Parker, personal communication). More recently a series of experiments have demonstrated the localization of Puf3 and several of its associated transcripts to the mitochondrial outer membrane [4,5] under environmental conditions in which we do not observe P-body formation (Dudley, Garmendia-Torres, and Drubin, unpublished results). It therefore seems likely that this dynamic co-localization is sensitive to environmental conditions and genetic background.

###### Measuring the significance of the differential expression of two gene groups. For each of the microarray experiments on deletion mutants, four repeats (2 technical replicates and 2 biological replicates) were generated, and the mean of the log_2_ ratios from the repeats for each spot was used for the analysis. The significance of the difference of expression levels between two gene groups was measured using the ‘non-parametric’ two-sample Kolmogorov-Smirnov test (KS-test) [6]. The *P*-value from KS-test corresponds to the probability of the null hypothesis – that two groups of continuous-valued numbers have the same distributions. In our setting, we considered comparing the expression values of (i) a gene group of interest and (ii) that of the rest of the genes. The significance of up-regulation (down-regulation) of group (i) can be quantified by a *P*-value from KS-test with an alternative hypothesis that the values from group (i) are larger (smaller) than those from group (ii).

**Measuring the significance of Puf3 motif scores for Dhh1 module genes.** We compared the Puf3 motif scores of (i) Puf3 targets within 153 Dhh1 module genes (108 genes) with those of (ii) the rest of the Puf3 target genes (39 genes) identified by the protein-RNA binding assay of Gerber et al. [7] who used the motif finding tool MEME (Multiple EM for Motif Elicitation) [8] to search for the Puf3 motif within the targets identified by their assay. The distributions of the Puf3 motif scores for two groups (i) and (ii) are shown in **Figure S3c**. To measure the significance of the difference of motif scores between groups (i) and (ii), we used the two-sample non-parametric Kolmogorov-Smirnov test (hereafter, KS-test) [6] with the alternative hypothesis that the motif scores of (i) are higher than those of (ii). The resulting P-value is < 2.10x10^-96^, showing that the Puf3 motif scores within the Dhh1 module genes is significantly higher than those of other Puf3 targets.

**E-MAP Analysis.** Synthetic genetic array (SGA) technology [9] was used in a high-density E-MAP format [10,11] on a set of approximately 500 essential and non-essential genes involved in various aspects of RNA metabolism (Wilmes et al., submitted). Images were processed and analyzed using recently developed software developed for E-MAP analysis [12].

**References**

1. David L, Huber W, Granovskaia M, Toedling J, Palm CJ, et al. (2006) A high-resolution map of transcription in the yeast genome. Proc Natl Acad Sci U S A 103: 5320-5325.

2. Sheth U, Parker R (2003) Decapping and decay of messenger RNA occur in cytoplasmic processing bodies. Science 300: 805-808.

3. Hatfield L, Beelman CA, Stevens A, Parker R (1996) Mutations in trans-acting factors affecting mRNA decapping in Saccharomyces cerevisiae. Mol Cell Biol 16: 5830-5838.

4. Garcia-Rodriguez LJ, Gay AC, Pon LA (2007) Puf3p, a Pumilio family RNA binding protein, localizes to mitochondria and regulates mitochondrial biogenesis and motility in budding yeast. J Cell Biol 176: 197-207.

5. Saint-Georges Y, Garcia M, Delaveau T, Jourdren L, Le Crom S, et al. (2008) Yeast mitochondrial biogenesis: a role for the PUF RNA-binding protein Puf3p in mRNA localization. PLoS ONE 3: e2293.

6. Laha R, Chakravarti, I. & Roy, J. (1967) Handbook of Methods of Applied Statistics: John Wiley and Sons.

7. Gerber AP, Herschlag D, Brown PO (2004) Extensive association of functionally and cytotopically related mRNAs with Puf family RNA-binding proteins in yeast. PLoS Biol 2: E79.

8. Bailey TL, Elkan C (1995) The value of prior knowledge in discovering motifs with MEME. Proc Int Conf Intell Syst Mol Biol 3: 21-29.

9. Tong AH, Lesage G, Bader GD, Ding H, Xu H, et al. (2004) Global mapping of the yeast genetic interaction network. Science 303: 808-813.

10. Schuldiner M, Collins SR, Thompson NJ, Denic V, Bhamidipati A, et al. (2005) Exploration of the function and organization of the yeast early secretory pathway through an epistatic miniarray profile. Cell 123: 507-519.

11. Schuldiner M, Collins SR, Weissman JS, Krogan NJ (2006) Quantitative genetic analysis in Saccharomyces cerevisiae using epistatic miniarray profiles (E-MAPs) and its application to chromatin functions. Methods 40: 344-352.

12. Collins SR, Schuldiner M, Krogan NJ, Weissman JS (2006) A strategy for extracting and analyzing large-scale quantitative epistatic interaction data. Genome Biol 7: R63.
